# Species-energy relationships of indigenous and invasive species may arise in different ways – a demonstration using springtails

**DOI:** 10.1038/s41598-019-48871-1

**Published:** 2019-09-24

**Authors:** Anne M. Treasure, Peter C. le Roux, Mashudu H. Mashau, Steven L. Chown

**Affiliations:** 1 Centre for Invasion Biology, Department of Botany and Zoology, Stellenbosch University, Private Bag X1, Matieland, 7602 South Africa; 2 Department of Plant and Soil Sciences, University of Pretoria, Private Bag X20, Hatfield, 0028 South Africa; 3 School of Biological Sciences, Monash University, Victoria, 3800 Australia; 4 Present Address: South African Environmental Observation Network (SAEON), 8th Floor, The Towers South, Hertzog Blvd, Foreshore, Cape Town, 8001 South Africa

**Keywords:** Climate-change ecology, Evolutionary ecology, Invasive species, Animal physiology, Entomology

## Abstract

Although the relationship between species richness and available energy is well established for a range of spatial scales, exploration of the plausible underlying explanations for this relationship is less common. Speciation, extinction, dispersal and environmental filters all play a role. Here we make use of replicated elevational transects and the insights offered by comparing indigenous and invasive species to test four proximal mechanisms that have been offered to explain relationships between energy availability, abundance and species richness: the sampling mechanism (a null expectation), and the more individuals, dynamic equilibrium and range limitation mechanisms. We also briefly consider the time for speciation mechanism. We do so for springtails on sub-Antarctic Marion Island. Relationships between energy availability and species richness are stronger for invasive than indigenous species, with geometric constraints and area variation playing minor roles. We reject the sampling and more individuals mechanisms, but show that dynamic equilibrium and range limitation are plausible mechanisms underlying these gradients, especially for invasive species. Time for speciation cannot be ruled out as contributing to richness variation in the indigenous species. Differences between the indigenous and invasive species highlight the ways in which deconstruction of richness gradients may usefully inform investigations of the mechanisms underlying them. They also point to the importance of population size-related mechanisms in accounting for such variation. In the context of the sub-Antarctic our findings suggest that warming climates may favour invasive over indigenous species in the context of changes to elevational distributions, a situation found for vascular plants, and predicted for springtails on the basis of smaller-scale manipulative field experiments.

## Introduction

Identifying the factors which explain spatial variation in the species richness, abundance and biomass of organisms is a primary goal of ecology. Ultimately, these community attributes depend on variation in diversification rates (the sum of speciation and extinction), dispersal, and environmental filtering^1–5^. Much attention has been given to the relationships among these processes, with a large and growing variety of studies documenting relationships between richness and environmental variables, such as various forms of energy availability, at many different spatial scales^6,7^. Nonetheless, exploration of the explicit mechanisms underlying these relationships is less common. Moreover, in the case of environmental effects, such explorations are often restricted to consideration of single mechanisms, and frequently what has come to be known as the increased population size hypothesis (or, alternatively, the more individuals hypothesis), which is concerned with the way increasing community abundance lowers extinction risk of species with the smallest populations^8–11^.

In consequence, much focus has been given to the need for investigations which consider simultaneously the multiple mechanisms that may underlie richness variation across spatial extents (Table 1), and several authors have provided proposals for how this can be done^4,11–13^. Among these proposals lies the idea that the investigation of patterns in alien species may be insightful, because biological invasions offer natural experiments that can further inform theory^14,15^. Although caution has to be exercised in such analyses, because components of the alien species patterns may be explained by the history of human introduction^16^ and by factors such as residence time, which may account for much of the occupancy of these species^17^, they can offer significant insight into the mechanisms that underlie several macroecological patterns e.g.^14,18,19^.

**Table 1:** Mechanisms underlying relationships between energy and species richness based on two recent theoretical treatments^12,20^.

Mechanism	Synopsis
Time for speciation	Longer time periods provide more opportunity for speciation.
Diversification rate	Increased energy produces faster speciation or slower extinction rates.
Niche breadth	Higher energy results in greater abundance of preferred resources, a switch away from non-preferred ones, reduction in niche overlap, lower competition, and thus greater richness.
Niche position	Higher energy increases the abundance of rare resources and niche position resource specialists, leading to higher richness.
More trophic levels	Increased energy enables additional trophic levels to occur that are occupied by new consumer species so increasing richness.
Consumer pressure	As a consequence of other mechanisms, consumers are more abundant or diverse, so reducing prey populations and promoting co-existence, resulting in higher richness.
Sampling	Higher energy results in greater numbers of individuals, and random selection from a regional species pool with larger numbers of individuals results in an increased number of novel species in a focal assemblage.
Increased population size/more individuals	Higher energy areas support more individuals, leading to lower extinction rates, and thus greater numbers of species.
Dynamic equilibrium	Increased energy enables faster recovery rates from disturbance, reducing the time during which small population size-associated stochastic extinction is likely to occur, hence elevating richness.
Range limitation	As solar energy increases, climatic conditions are within the physiological tolerance range of more species.

In the case of richness gradients of invasive species, diversification-related mechanisms, such as diversification rate variation and time for speciation (i.e. variation among habitats in the time occupied and hence speciation opportunity in that habitat)^4,20,21^, are unlikely to be important because of the relatively short time, in evolutionary terms, for speciation to have taken place^22^. While ecological speciation is possible over several hundred generations^23^, typically, speciation takes place over longer periods e.g^.^^24^. Thus, in the case where invasive species are not still expanding their range see^17^, community attributes should be dominated by ecological processes operating at the population level, such as the effects of energy on abundance and in turn the way this translates to higher richness^12^. By contrast, for indigenous species, which have the potential to have occupied an area for a much longer period, speciation-related mechanisms are necessarily important, though influenced by spatial scale^4,5^.

Among the explanations for spatial variation in richness, eight proximal mechanisms have been proposed to explain increasing richness with increasing energy availability through space^12^ (Table 1). The sampling mechanism proposes that higher energy results in greater numbers of individuals, and random selection from a regional species pool with larger numbers of individuals results in an increased number of novel species in a focal assemblage. In essence, this is a null expectation. In the more individuals or population size mechanism, higher energy areas support more individuals, leading to lower extinction rates, and thus greater numbers of species^8^. The niche breadth mechanism proposes that higher energy results in greater abundance of preferred resources, a switch in a given group away from non-preferred ones, reduction in niche overlap, lower competition, and thus greater richness. The niche position mechanism posits that higher energy increases the abundance of rare resources and thus resource specialists, leading to higher richness^25^. In the case of the more trophic levels mechanism, increased energy enables additional trophic levels to occur that are occupied by new consumer species, so increasing richness. The main prediction of the dynamic equilibrium mechanism is that increased energy enables faster recovery rates from disturbance, reducing the time during which small population size-associated stochastic extinction is likely to occur, hence elevating richness. The range limitation mechanism posits that as energy increases, climatic conditions are within the physiological tolerance range of more species. Finally, the consumer pressure mechanism proposes that as a consequence of higher energy, consumers are more abundant or diverse, so reducing prey populations and promoting co-existence, resulting in higher richness.

These proximal mechanisms apply both to indigenous and invasive species, though differences in their relative importance may be expected. For example, enemy release, one hypothesis for the success of invasive species^26^, would preclude the importance of the consumer pressure mechanism. Nonetheless, how the mechanisms differ among these two groups has not been explicitly examined, at least over relatively small spatial scales (there are some examples of recent work over large spatial extents^19,27^), as is the case with simultaneous examinations of the multiple proximal mechanisms underlying spatial variation in abundance and richness more generally^28^.

Here we therefore examine simultaneously multiple likely mechanisms for spatial species richness variation among a group of co-occurring indigenous and invasive alien species. We do so for a group rarely considered in such work, the springtails, but one which is highly diverse and important in soil ecosystems globally, making it a useful model for investigating macroecological questions^29–31^. We undertake the investigation using a replicated 1000 m elevational gradient on sub-Antarctic Marion Island, recognizing the utility of elevational gradients for examining macroecological questions^4^.

We assume that owing to the short history of human occupation of the island (sporadically since the 1800s and consistently only since 1947)^32^, and the absence of any endemic multi-species springtail clades^33^ on this geologically young island^32^, diversification rate variation^20,21^ has not played a role in species richness patterns for either group. Given the small spatial extent of the island (300 km^2^), we assume that time for speciation may have played a role^4,21^. If such a mechanism contributes differentially to indigenous and invasive species, it should be manifest as a steeper slope in richness gradients for the invasive than the indigenous species given substantially different histories of these two groups (indigenous species with a *ca*. 500 000 year history; invasive species with maximally 200 years on the island^34^).

Thus, we first examine whether differences in the relationship between species richness and energy availability exists between these two groups. We use energy availability because elevation itself is not the environmental gradient which affects richness^4^. Energy availability can be measured in a variety of ways^35^. Although temperature is not a measure of energy availability, it has an influence on energy availability especially in environments, such as Marion Island, which are not water limited. Here, direct measures of energy availability, such as Net Primary Productivity, are not available because of the difficulty of estimates in the higher elevation polar desert areas where typically bryophyte or vascular vegetation is absent. Solar radiation is also sometimes used as a proxy for available energy, but again for the soil fauna it is difficult to obtain reasonable estimates, and for the island, even surface estimates are unavailable at the resolution required, especially for understanding seasonal variation. Hence, we have used temperature as a proxy for energy availability. Because both geometric constraints^36^ and the species-area relationship^37^ should be considered *a priori* in the context of elevational variation in richness, we also test for these effects, examining the latter together with temperature variation as an explanation for variation in richness. We then test explicitly four of the eight proximal explanations for richness-energy relationships set out by Evans *et al*.^12^ and specifically the sampling, more individuals, dynamic equilibrium, and range limitation hypotheses. The niche breadth, niche position, more trophic levels, and consumer pressure explanations were not considered. No evidence for interspecific competition exists for the group on the island^38^, and none for specialization – reflected by the ease with which both indigenous and invasive species can be reared using food sources from elsewhere than on the island^39^ and by the broad habitats of the invasive species in their home ranges^33^, thus excluding the niche position and breadth mechanisms. The more trophic levels mechanism was excluded because all of the species belong to the same trophic level^39^. The consumer pressure hypothesis was not considered because spiders are the only major predators of springtails on the island, and the density of spiders is higher in areas with greater thermal energy availability^40,41^ - areas where springtail abundance is also higher^38^, contrary to the mechanism.

## Methods

### Study area, species and sampling approach

Sub-Antarctic Marion Island (MI) (46°54′S, 37°45′E) is the larger (300 km^2^) of two biologically very similar islands in the Prince Edward Island group. This sub-Antarctic, Indian Ocean island has a cool, wet, windy climate, which varies considerably with elevation (see Chown and Froneman^32^ for review). Two major biomes are present: sub-Antarctic tundra, which predominates in lowland areas, and sub-Antarctic polar desert, restricted to high elevations. Sixteen species of springtails have been recorded from the island, of which six are introduced and invasive^33,38^. Here, invasive species mean those alien species which have colonised the entire lowland extent of the island, and thus are not dispersal limited (see evidence from Gabriel *et al*.^38^; Hugo^42^). The invasive species are European and in their native ranges occur across the full range of temperatures found on the island^32,43,44^.

**Figure 1:**
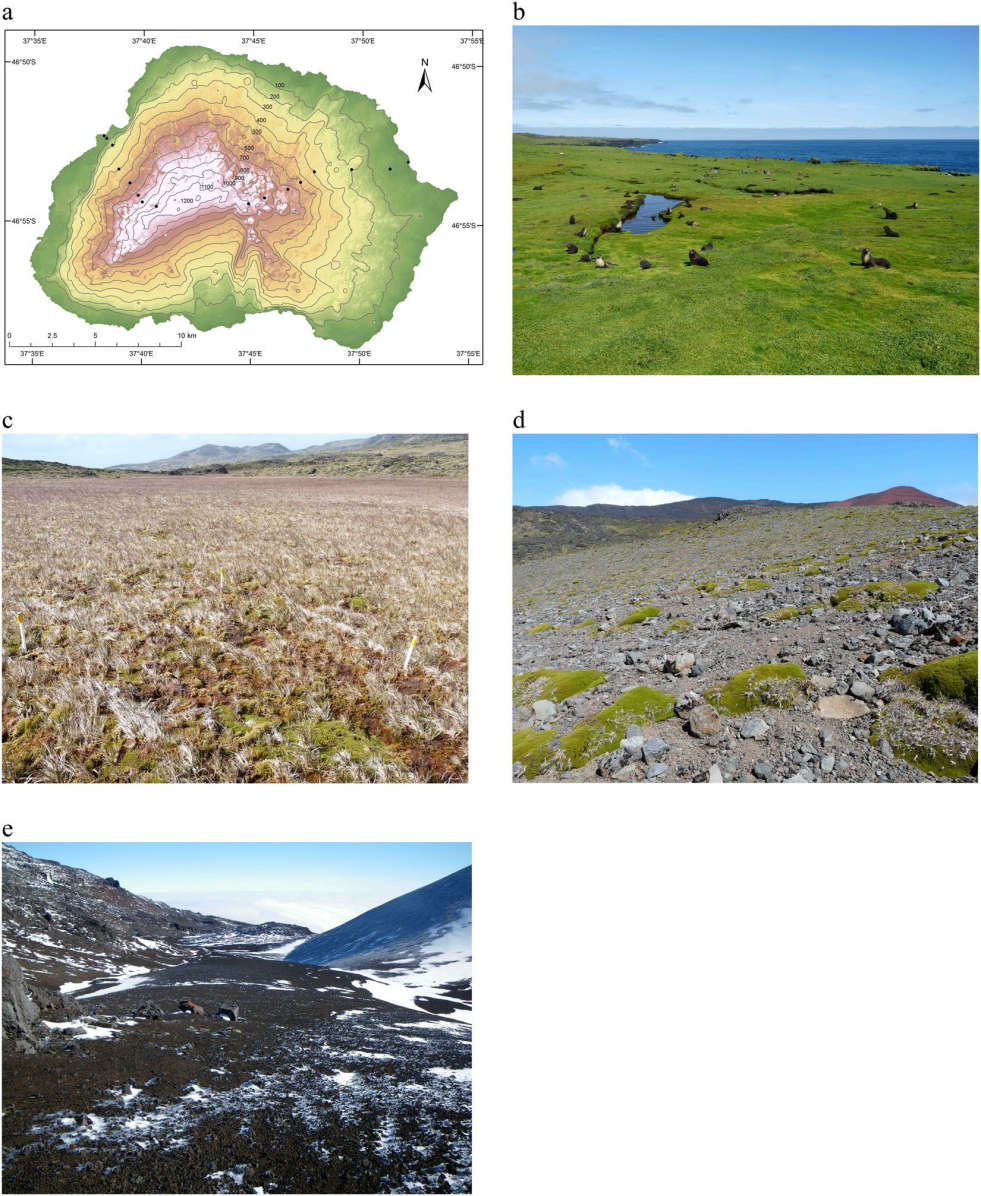
(**a**) Position of each of the sites sampled along two altitudinal transects on Marion Island, one on the eastern side of the island (east transect) and one on the western side (west transect), from the coast to 1000 m a.s.l. The research station is located close (<1 km) to the east transect coastal site. (Digital Elevation Model and image courtesy of David Hedding). The four habitat complexes sampled in the study include (**b**) biotic, (**c**) mire, (**d**) fellfield, and (**e**) polar desert.

Species composition and abundance were investigated along two altitudinal transects, one on the eastern side of the island and one on the west (Fig. 1). Because some springtail species on Marion Island show pronounced seasonality in abundance^45^, both transects were sampled twice: once in winter (June-July 2008) and once in summer (February 2009). The habitat complexes see^32^ sampled are representative of those found at the specific altitudes: biotically influenced vegetation (10 m a.s.l.), mires (50 m a.s.l. and 200 m a.s.l.), fellfield (400 m a.s.l. and 600 m a.s.l.) and polar desert (750 m a.s.l., 850 m a.s.l. and 1000 m a.s.l.), resulting in eight sites per transect (Fig. 1). At each site, the GPS coordinates and the habitat type and/or plant species composition were recorded.

At each site, 20 samples were taken either using a 34 mm diameter soil corer or, for the polar desert, by removing the top 5 cm of soil and stones from a 10 cm × 10 cm area. The sampling design varied between the lower five sites and the upper three sites because in the upper sites no vegetation is present and the scoria (loose gravel) substrate cannot be sampled using a standard soil corer (full sampling descriptions are provided in the Supporting Information Appendix S1). Previous work has found comparable results using these approaches^38^.

For each of the samples taken at the five lower elevation sites, springtails were extracted from the top 10 cm of cores using a Macfadyen high-gradient extractor into 40% ethanol (extraction protocol: 2 days at 25 °C followed by 2 days at 30 °C^45^) for the east transect. Samples from the west transect were extracted using Tullgren funnels in a field hut. The difference in extraction method was not considered a source of bias because densities from the western samples were well within those found by Hugo *et al*.^46^ and Hugo^42^ for comparable sites after high gradient extraction. For each of the samples from the three higher elevation sites, the protocol followed Barendse and Chown^45^. Material was washed (using water previously sieved through 125 µm mesh) and sieved through 125 µm mesh three times. A separate mesh was used for each sample and after sample washing each mesh was placed in a separate 35 ml plastic container with 40% ethanol. All samples were further sorted at the research station where all individuals were identified to species level using a Leica MZ75 microscope (identification keys^33^; Janion-Scheepers *et al*. unpublished manuscript). All samples are currently stored in 99% ethanol at Stellenbosch University.

### Energy availability

We use temperature as a metric of energy availability. In systems which are not water limited, as is the case for this cool island which typically receives in excess of 1900 mm of precipitation per year^32^, plant productivity is constrained by the influence of temperature on both growing season length and on rates of leaf metabolism^35,47^. Thus, we consider temperature a reasonable proxy for productivity in such systems^35^. While there are other proxies for energy availability, such as solar radiation or potential evapotranspiration^35^, these have not been measured across the island. This proxy approach should be kept in mind in considering the outcomes of the tests we present here. Moreover, variation in temperature also plays a direct physiological role in ectotherm physiology^48^, making it applicable to both tests of the purely productive energy forms of species-energy mechanisms, and those that have to do with temperature variation directly^12,35^.

Soil temperature, appropriate for springtails, was measured using Thermochron iButton temperature dataloggers (Model DS1921, accurate to ±0.5 °C, Dallas Semiconductors, Dallas, TX, USA) placed ca. 2 cm below the ground surface at each site. Hourly temperatures were recorded for three winter months (mid-May 2008 to mid-August 2008) and three summer months (mid-November 2008 to mid-February 2009). Temperature data were processed to obtain mean daily minimum, maximum, mean and temperature range for each site in R (version 2.12.0)^49^ (hereafter: short-term data).

A longer-term data series (hereafter: long-term data), processed for the same variables described above, was obtained by recording soil temperatures on an hourly basis at *c*. 100 m intervals from sea level to 750 m, across the eastern slope of Marion Island between 2002 and 2009 using iButton temperature dataloggers^50^. Missing data (due to datalogger loss, exposure, or damage) were interpolated using a sinusoidal function (i.e. to approximate daily temperature cycles, written in R2.12.0), with the initial and final interpolated values (as well as the amplitude of the sinusoidal curve) calculated from the temperature records of the 48 hours before and after the missing data.

### Sampling adequacy and species richness

Raw abundance data for indigenous and invasive species were converted to density (individuals.m^−2^) to enable comparison of data from the two different sampling approaches (though we use the term abundance hereafter). To determine sampling adequacy for each site, sample-based (i.e. per core or 10 cm × 10 cm sample) rarefaction curves were calculated using the Mao Tau moment-based interpolation method in EstimateS^51^.

### Richness-energy relationships, geometric constraints and area effects

Because the level of interest here is variation among sites with different energy availability, and because multiple samples were taken to ensure adequate site sampling^52^, the site level was used as the level for investigation.

To test for geometric constraints, species richness data were compared with null model predictions using a Monte Carlo simulation procedure, Mid-Domain Null, in Visual Basic for Excel^53^. Randomisation techniques were used, based on 50 000 simulations sampled without replacement from empirical range sizes, and a regression of the empirical values on predicted values provided *r*^2^, slope and intercept as estimates of the fit of the null model^53^. We used the coastal and high elevation end-points for each transect as the limits to the domains, to avoid biasing assessments against a mid-domain effect, which would have been the case had we used a single domain (i.e. a coast to coast approach). Springtails have not been found above 1000 m, largely because higher elevations (the island rises to 1230 m) are represented by just a few isolated peaks^32^.

To examine the influence of surface area on richness, we used generalised linear models (GLMs, assuming a Quasipoisson distribution to correct for overdispersion, and with a log link function, implemented using the glm function in R2.12.0^54^) with species richness as the response variable, and surface area and mean temperature (from the short-term data) as the predictor variables to establish the role of surface area. Sites were allocated to altitudinal bands and the surface area of each of these bands extracted from Meiklejohn and Smith^55^. Full models included energy and log transformed surface area as variables, and single term models included either temperature or log(area) individually as variables. We re-assessed the relationships using generalised estimating equation models to take the potential effects of spatial autocorrelation into account^56^.

Following this assessment, which revealed relationships between temperature and richness, we compared the estimates for the richness-mean temperature relationship for the indigenous and invasive species to determine whether they differ. We used GLMs (Quasipoisson distribution, log link function) with species richness as the response variable, and mean temperature (from the short-term data) and species category (indigenous/invasive) as the predictor variables, specifically focussing on whether the interaction term is significant, indicating different forms of the relationships for indigenous and invasive species.

### Tests of the potential mechanisms

Following Evans *et al*.’s^12^ recommendations, to distinguish the sampling and increased population size hypotheses, the relationships between species abundance and richness (using S_obs_ for the sampling explanation and the Jacknife2 estimator^57^ for the increased population size explanation, calculated for each of the sites using the vegan package in R2.12.0), species richness and mean temperature, and abundance and mean temperature were investigated for both indigenous and invasive species as well as the combined assemblages using GLMs (Quasipoisson distribution, log link function). To test for decelerating relationships, the GLMs were rerun using a squared term for the predictors. In all cases the units were sites on each transect, resulting in n = 16. If the relationships are not positive and decelerating using the estimator, but are for the raw data, the increased population size explanation in this specific form can be rejected^12^.

The dynamic equilibrium explanation posits that elevated energy enables populations to recover faster from disturbance. On Marion Island, large-scale disturbances are mostly from low temperature events^32,58^ (Supporting Information Appendix S2). Temperature disturbances in the form of two thresholds were considered. These are 0 °C (the freezing point of water), and the mean lower development threshold (LDT) of the springtail eggs (the most sensitive developmental stage^39^). Both the number of times the thresholds were crossed and the maximum duration spent below the thresholds at any one time (hereafter – longest duration) were assessed as disturbance events for each site using the short-term and long-term temperature data and empirical values of the springtail egg LDTs (Table S1)^39^. The effects of thresholds (number of events and longest duration of sub-zero events and those below the LDT) on the abundance (summed density at each site) of indigenous and invasive species were investigated using generalised linear models (in R3.1.0, Quasipoisson distribution, log link function; Deviance Explained (DE) was calculated using the BiodiversityR package). Minimum adequate model selection was adopted^54^.

The range limitation explanation proposes that species occur in areas where they can meet their physiological requirements^12^, and assumes that more species can tolerate warmer than cooler conditions^9^. To test this idea and to distinguish possible outcomes from the dynamic equilibrium explanation, the summed abundances of indigenous and invasive species groups were compared to the minimum and maximum temperatures recorded at each site (i.e. winter mean daily minimum and summer mean daily maximum), and to the number of generations possible at each elevation. Conditions that do not exceed springtail tolerances and enable multiple generations are more likely to result in positive population growth, than those that limit populations either via development or thermal tolerances^59,60^. Both the short-term and long-term temperature datasets were used (see above). The number of generations possible was calculated using the sum of effective temperatures (SET)^61^. Mean SET values were calculated for the indigenous and invasive groups of species at each site (detailed information in Appendix S2). The effects of minimum and maximum temperatures and generations possible on the abundances of indigenous and invasive species were investigated using generalised linear models (as above). Minimum adequate model selection was adopted^54^.

## Results

**Figure 2:**
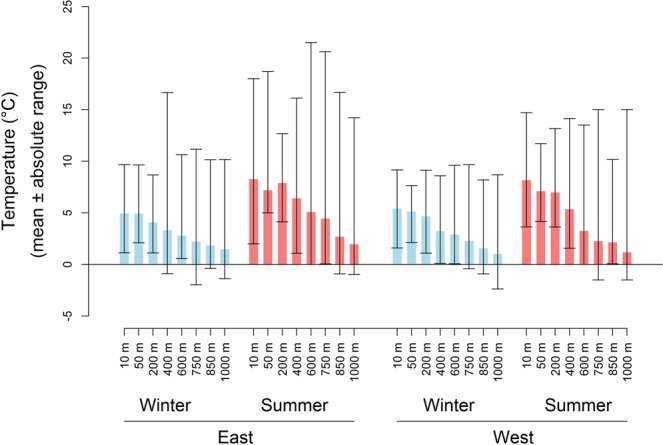
Mean and absolute temperatures for the short-term data set (2008–2009) during winter and summer along the east and west altitudinal transects on sub-Antarctic Marion Island.

Strong elevational declines in temperature were found (Fig. 2; Table S2). The low temperature values, together with site-specific rainfall records indicating rainfall above 1000 mm over the year (Fig. S1), substantiated the assumption that the soil ecosystem is not water limited. Eight indigenous and four invasive springtail species were sampled on the west transect, and seven indigenous and four invasive species were found on the east. When summer and winter data were pooled, rarefaction curves indicated that sampling had reached an asymptote for most sites (Fig. S2), and was therefore considered adequate for the summed data, which was used for all further analyses (Table S3).

Fit to the geometric constraints null model was poor for observed species richness for both transects (east: *r*^2^ = 0.003; west: *r*^2^ = 0.064) and the relationships were not significant (east: *p* = 0.66, slope = 0.11, intercept = 6.92; west: *p* = 0.05, slope = 0.42, intercept = 5.36). Similar outcomes were found for estimated richness (east: *r*^2^ = 0.466, *p* = 0.06, slope = 0.93, intercept = −0.18; west: *r*^2^ = 0.237, *p* = 0.22, slope = 1.20, intercept = −3.22).

**Figure 3:**
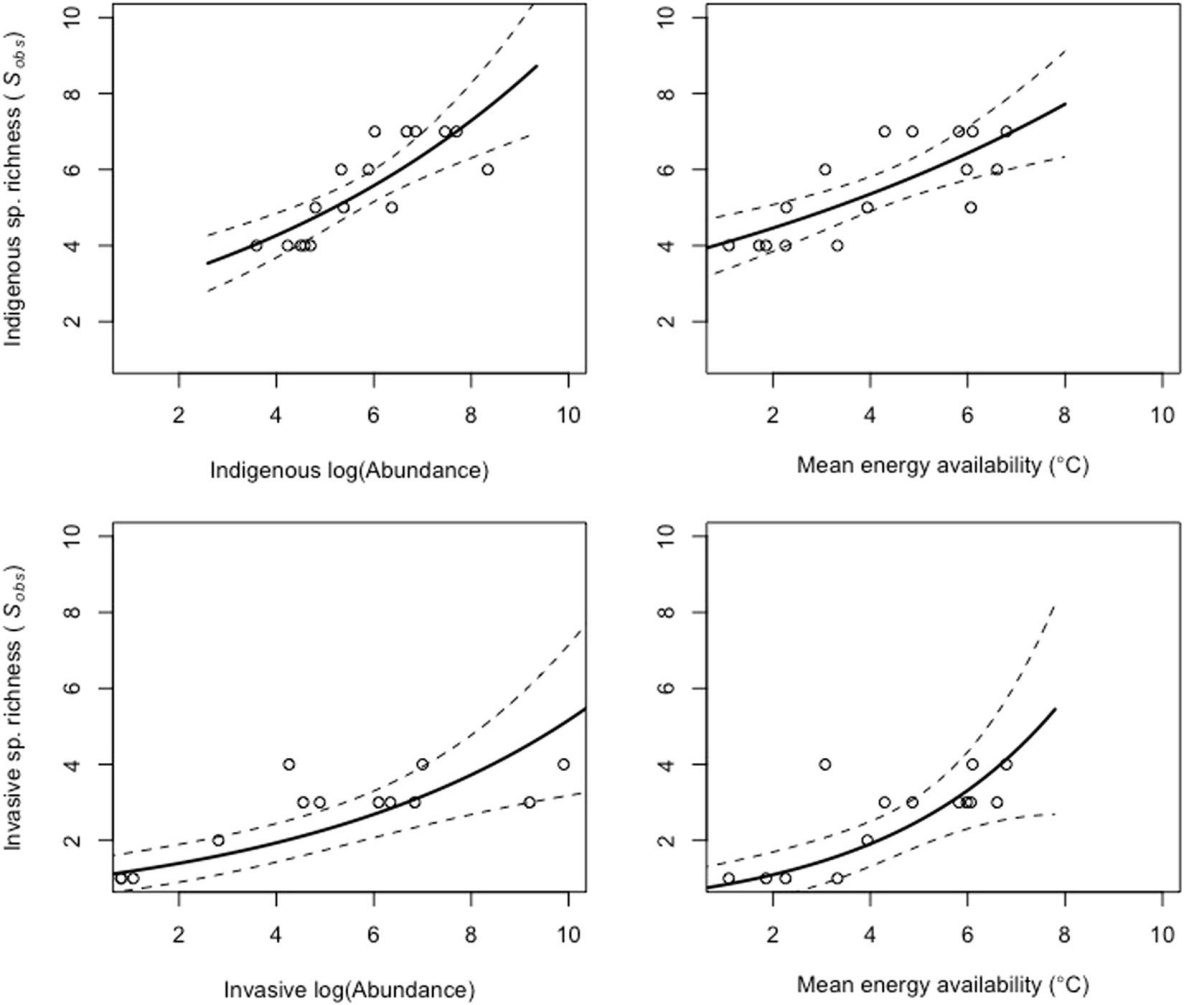
Predictions under the Poisson GLZ models for the sampling mechanism, of species richness (*S*_*obs*_) versus abundance, and mean thermal energy availability for (**a**) indigenous and (**b**) invasive springtail species on Marion Island. 95% confidence intervals are shown using dashed lines; open circles are observed values.

Although both surface area and mean temperature were significant predictor variables for indigenous and invasive species richness in the single term GLMs, surface area was not significant in the full models (Table 2) and was therefore not considered any further (though bearing in mind that these variables are collinear, *r*^2^ = 0.63, *p* < 0.001). Similar outcomes were found using the GEE models, which explicitly consider spatial relationships (Table S4), though in this case significance values could not be fully relied on because Poisson rather than Quasipoisson distributions were required in the models. Positive species-energy relationships were found for the indigenous and invasive species (Table 3; Fig. 3), with a significant difference in the form of the relationship (Table 4).

**Table 2:** Outcomes of the generalised linear models examining the relationships between either indigenous or invasive springtail species richness, and mean temperature and log transformed surface area of altitudinal bands.

(A) Full models	df	Estimate	s.e.	*X* ^2^	*p*
**Indigenous species**					
(DE = 63.52%; df = 15)					
mean energy	1	0.056	0.032	2.986	0.084
log(area)	1	0.309	0.219	2.015	0.156
**Invasive species**					
(DE = 51.74%; df = 15)					
mean energy	1	0.228	0.110	4.136	0.042
log(area)	1	0.454	0.718	0.405	0.525
**(B) Single term models**	**df**	**Estimate**	**s.e**.	***X*** ^**2**^	***p***
**Indigenous species**					
mean energy (DE = 57.93%; df = 15)	1	0.092	0.021	19.138	<0.0001
log(area) (DE = 55.23%; df = 15)	1	0.607	0.145	17.537	<0.0001
**Invasive species**					
mean energy (DE = 50.37%; df = 15)	1	0.277	0.074	15.666	<0.0001
log(area) (DE = 37.66%; df = 15)	1	1.578	0.516	9.875	0.002

s.e. = standard error, DE = % deviance explained.

**Table 3:** Outcomes of the generalised linear models examining the relationships between species richness (using S_*obs*_ and the estimator Jacknife2), abundance, and mean temperature for the indigenous, invasive and combined springtail species.

	Single term models			Squared term models		
	Estimate	s.e.	*p*	Estimate	s.e.	*p*
**Species richness vs. temperature**						
*S*_*obs*_						
Indigenous (DE = 57.93%; df = 15)	0.092	0.021	0.001	−0.021	0.015	0.178
Invasive (DE = 50.37%; df = 15)	0.277	0.074	0.002	−0.076	0.055	0.189
Combined (DE = 65.13%; df = 15)	0.142	0.029	<0.001	−0.031	0.020	0.152
**Jacknife2**						
Indigenous (DE = 5.56%; df = 15)	0.035	0.038	0.378	−0.042	0.027	0.139
Invasive (DE = 24.26%; df = 15)	0.227	0.113	0.065	−0.231	0.079	0.012
Combined (DE = 24.47%; df = 15)	0.098	0.049	0.063	−0.088	0.029	0.009
**Species richness vs. abundance**						
*S*_*obs*_						
Indigenous (DE = 64.54%; df = 15)	0.308	0.061	<0.001	−0.245	0.093	0.021
Invasive (DE = 57.29%; df = 15)	0.376	0.079	<0.001	−0.188	0.047	0.002
Combined (DE = 71.18%; df = 15)	0.374	0.064	<0.001	−0.203	0.080	0.025
**Jacknife2**						
Indigenous (DE = 35.79%; df = 15)	0.284	0.102	0.015	0.169	0.177	0.355
Invasive(DE = 22.28%; df = 15)	0.279	0.136	0.060	−0.250	0.108	0.037
Combined (DE = 46.97%; df = 15)	0.345	0.095	0.003	0.200	0.138	0.172
**Abundance vs. temperature**						
Indigenous (DE = 22.87%; df = 15)	0.059	0.029	0.067	−0.028	0.021	0.212
Invasive (DE = 76.40%; df = 15)	0.465	0.074	<0.001	−0.068	0.054	0.231
Combined (DE = 49.52%; df = 15)	0.075	0.021	0.003	−0.004	0.016	0.778

Models including single and squared term predictor variables are shown. Deviance explained (DE) values are shown for models with single term predictor variables. s.e. = standard error.

**Table 4:** Outcomes of the generalised linear model examining the relationship between species richness, and mean temperature (from the short-term data) and species category (indigenous or invasive).

	df	Estimate ± s.e.	*X* ^2^	*p*
Response: *S*_*obs*_ (DE = 76.43%; df = 31)				
temperature	1	0.092 ± 0.034	24.771	<0.0001
sp. category: invasive	1	−1.774 ± 0.349	65.499	<0.0001
interaction	1	0.185 ± 0.067	7.912	0.005

s.e. = standard error, DE = deviance explained.

The strong positive relationships between energy and richness tended not to be present with the richness estimator and a similar effect was found for the relationship between richness and abundance, although the effect for richness was larger for the indigenous than the invasive species (Table 3). Overall, the variable positive and decelerating relationships between richness and energy, richness and abundance, and between abundance and temperature suggested that neither the sample size nor sampling explanations could account for the energy-richness relationship.

Significant relationships were found between species abundance and disturbance thresholds for the long-term, but not for the short-term datasets (Table 5). In the case of the latter, abundance of indigenous species tended to decline with the number of events below 0 °C, but weakly so, and a weak positive relationship between duration below LDT and abundance was found, with less than 50% of the deviance being explained by the model overall. For the invasive species 93% of the deviance was explained by a single negative relationship between the number of events below the LDT and abundance.

**Table 5:** Outcomes of the generalised linear models examining the relationships between either indigenous or invasive springtail abundance, and events below 0 °C, longest duration below 0 °C, events below LDT and longest duration below LDT, for the short-term temperature data set (A) and the long-term data set (B).

(A) Short-term temperature data set	df	Estimate	s.e.	*X* ^2^	*p*
**Indigenous species**					
(DE = 38%; df = 15)					
Longest duration below 0 °C	1	−0.039	0.033	0.096	0.052
Events below LDT	1	0.004	0.028	1.356	0.880
Longest duration below LDT	1	0.002	0.003	0.793	0.392
**Invasive species**					
(DE = 65%; df = 13)					
Events below LDT	1	0.018	0.146	0.015	0.905
Longest duration below LDT	1	−0.016	0.035	0.834	0.361
**(B) Long-term temperature data set**	**df**	**Estimate**	**s.e**.	***X*** ^**2**^	***p***
**Indigenous species**					
(DE = 48%; df = 15)					
Events below 0 °C	1	−0.003	0.002	6.91	0.008
Longest duration below LDT	1	0.002	0.001	6.83	0.009
**Invasive species**					
(DE = 93%; df = 13)					
Events below LDT	1	−0.010	0.001	118.14	<0.001

s.e. = standard error, DE = % deviance explained.

In the context of potential range limitation, no variation in abundance was explained for the indigenous species (Table 6), for either the short-term or long-term datasets, although for invasive species abundance increased with minimum temperature and accounted for much of the deviance in the data (DE = 64 to 92%) (Table 6).

**Table 6:** Outcomes of the generalised linear models examining the relationships between either indigenous or invasive springtail abundance, and minimum temperature, maximum temperature and possible generations, for the short-term temperature data set (A) and the long-term data set (B).

(A) Short-term temperature data set	df	Estimate	s.e.	*X* ^2^	*p*
**Indigenous species**					
(DE = 15%; df = 15)					
Minimum temperature	1	0.410	0.334	1.521	0.217
Possible generations	1	−0.855	1.072	0.937	0.333
**Invasive species**					
(DE = 64%; df = 15)					
Minimum temperature	1	1.799	0.672	25.75	<0.0001
**(B) Long-term temperature data set**	**df**	**Estimate**	**s.e**.	***X*** ^***2***^	***p***
**Indigenous species**					
(DE = 12%; df = 15)					
Minimum temperature	1	0.238	0.511	1.192	0.275
**Invasive species**					
(DE = 92%; df = 15)					
Minimum temperature	1	2.759	0.579	90.93	<0.0001

s.e. = standard error, DE = % deviance explained.

## Discussion

Species richness declined monotonically in both transects, in keeping with patterns found for some, but not all elevational gradients in richness^4,37^. No evidence was present for the influence of geometric constraints, in keeping with the relatively small scale of the work^62^. Although richness was positively related both to energy and area, area tended not to be significant in the models including both variables (bearing in mind the variables are collinear). Thus, positive species-energy relationships exist for this system. Moreover, the variation in richness accounted for by mean temperature (*ca*. 50%) was in keeping with (or perhaps slightly higher than) that found for other organisms at this spatial extent^6,63^.

Differences between the indigenous and invasive species in the form of the species-energy relationship were significant, with a steeper relationship for the invasive species. The outcome, and especially the shallower slope of the richness-energy relationship for the indigenous species suggests that a time for speciation mechanism^4,21^ might be important. In other words, occupation of indigenous springtails of multiple habitats over a prolonged period of the island’s history would have enabled speciation in all of them (see discussion in Myburgh *et al*.^64^), thus reducing the slope of the species-energy relationship. The lack of springtail clades which have diversified on the island suggests, however, that the mechanism is unlikely to have played a large role. Nonetheless, at least two species are endemic or suspected to be so^33,65^, indicating that local speciation has taken place.

By contrast, much of the evidence points to variation in population size as an important explanation for the richness-energy relationships, but in a manner different to that proposed directly by the more individuals hypothesis. Indeed, of the mechanisms for the species-energy relationship compiled by Evans *et al*.^12^ that we examined, neither the more individuals hypothesis, nor some form of sampling artefact were supported. By contrast, the generalised linear models suggested that both the range limitation and dynamic equilibrium mechanisms are plausible for the richness-energy relationships, especially in the case of the invasive species. Although Evans *et al*.^12^ rightly considered the dynamic equilibrium and range limitation mechanisms distinct from the more individuals hypothesis, given that the former have to do with productive energy and the latter may incorporate direct effects of temperature too, it is clear that they constitute a subset of a broader population size-related set of mechanisms. Indeed, they both invoke variation in population size or abundance associated with an environmental filter^4^, supporting a more general emphasis on population size variation as a factor explaining energy-richness relationships^13^.

In the case of the dynamic equilibrium mechanism, small populations are unable to recover from disturbance, and are more prone to negative effects of environmental stochasticity^66,67^ which ultimately leads to local extirpation. How this might play out for springtails on the island is clear. Invasive species typically have higher lower developmental thresholds for development than do their indigenous counterparts^39^, which would limit population growth at low temperature. Similarly, differences in the ability of the two groups of species to cope with temperatures below freezing have been recorded^68^, with the indigenous species capable of dealing with the most extreme events found on the island. Moreover, variation in temperatures along the altitudinal gradient shows that low temperature disturbance events are more common at higher rather than lower altitudes (see also discussion in Lee *et al*.^58^).

In the case of range limitation, lack of physiological tolerance accounts for either an inability to exist at a site, or low abundances^9,15^, which in turn result in higher extinction probability. These processes mean that even if a site is within the dispersal range of a species, extinction-related processes preclude viable populations^58,69,70^. For the springtails considered here, the indigenous species show no limitation by low or high temperatures, perhaps unsurprising given their tolerance limits and the relationship between egg development and temperature^39,68^. By contrast the strong relationship between minimum temperature and invasive abundance suggests that their low temperature sensitivity^39,68^ may be limiting.

Differences in the likely mechanisms underlying species-energy relationships among the indigenous and invasive species illustrate that while the patterns for these two groups of species appear similar in broad terms (richness declines with decreasing energy), the explanations for them may be quite different. In this case, dynamic equilibrium mechanisms apply to both groups, while range limitation plays a larger role for the invasive than the indigenous species. Moreover, the lower variation explained in the models for the indigenous species, yet the significant, though shallow, species-energy relationship suggests that speciation-associated mechanisms may also be important for them, in keeping with studies suggesting that time for speciation effects are relevant over small spatial extents^21^. Importantly, a strong relationship between abundance and temperature for the invasive species across the gradient, but little effect on the indigenous species, adds support to the idea that environmental constraints are more important for the former group^71,72^, despite their origins in cold-temperate Europe.

While these differences might at first appear to constrain lessons from comparisons among the two groups of species, they may prove insightful by enabling various likely mechanisms in an area to be disentangled by relying on such *a priori* expectations of differences (see also Marquet *et al*.^73^; Hawkins *et al*.^74^). In the context of the specific systems of the sub-Antarctic islands, the strong influence of temperature on richness and on abundance patterns of the invasive compared with the indigenous species suggests also that rising temperatures and declining rainfall on many of the islands e.g.^75–78^ will enable the spread of invasive species to higher elevations, as has already been documented for plants^79^. Field manipulations have supported an assumption of greater population-level success for invasive species at local scales under warming and drying^80^, with this broader scale analysis providing further substantiation for this idea. Nonetheless, differentiation of temperature effects from energy availability *per se* is still required, given that we used temperature as a proxy for the latter^35^. Only with well-developed, fine resolution data on either net primary productivity or proxies such as potential evapotranspiration or solar radiation will this be possible. While estimates of primary productivity have been made for Marion Island^81,82^, these have not been spatially explicit and remain challenging for polar desert environments that either have an interstitial flora, or high cloud cover(precluding remote-sensing estimates), or both.

## Supplementary information

Supplementary information

## Data Availability

The core level site data collected in this study are publicly available from the Monash Figshare repository (10.26180/5cf4f64629f6a).

## References

[1] Hubbell, S. P. *The Unified Theory of Biodiversity and Biogeography* (Princeton University Press 2001).

[2] Hawkins BA (2003). Energy, water, and broad-scale geographic patterns of species richness. Ecology.

[3] Wiens JJ, Graham CH, Moen DS, Smith SA, Reeder TW (2006). Evolutionary and ecological causes of the latitudinal diversity gradient in hylid frogs: treefrog trees unearth the roots of high tropical diversity. Am Nat.

[4] Graham CH (2014). The origin and maintenance of montane diversity: integrating evolutionary and ecological processes. Ecography.

[5] Vellend, M. *The theory of ecological communities* (Princeton University Press 2016).

[6] Belmaker J, Jetz W (2011). Cross-scale variation in species richness–environment associations. Global Ecol Biogeogr.

[7] Belmaker J, Jetz W (2015). Relative roles of ecological and energetic constraints, diversification rates and region history on global species richness gradients. Ecol Lett.

[8] Srivastava DS, Lawton JH (1998). Why more productive sites have more species: an experimental test of theory using tree-hole communities. Am Nat.

[9] Currie DJ (2004). Predictions and tests of climate-based hypotheses of broad-scale variation in taxonomic richness. Ecol Lett.

[10] Hurlbert AH, Jetz W (2010). More than “more individuals”: the nonequivalence of area and energy in the scaling of species richness. Am Nat.

[11] Pigot AL, Tobias JA, Jetz W (2016). Energetic constraints on species coexistence in birds. PLoS Biol.

[12] Evans KL, Warren PH, Gaston KJ (2005). Species-energy relationships at the macroecological scale: a review of the mechanisms. Biol Rev.

[13] Hurlbert AH, Stegen JC (2014). When should species richness be energy limited, and how would we know?. Ecol Lett.

[14] Sax, D. F. & Gaines, S. D. The biogeography of naturalized species and the species-area relationship in *Conceptual ecology and invasion biology: reciprocal approaches to nature* (eds Cadotte, M. W., McMahon, S. M. & Fukami, T.) 449–480 (Springer, 2006).

[15] Brown JH (2014). Why are there so many species in the tropics?. J Biogeogr.

[16] Blackburn, T. M., Lockwood, J. L. & Cassey, P. *Avian invasions*. *The ecology and evolution of exotic birds* (Oxford University Press, 2009).

[17] Wilson JRU (2007). Residence time and potential range: crucial considerations in modelling plant invasions. Divers Distrib.

[18] Wiens JJ, Graham CH (2005). Niche conservatism: integrating evolution, ecology, and conservation biology. *Ann Rev*. Ecol Evol S.

[19] Moser D (2018). Remoteness promotes biological invasions on islands worldwide. PNAS.

[20] Wiens JJ (2011). The causes of species richness patterns across space, time, and clades and the role of ecological limits. Q Rev Biol.

[21] Pontarp M, Wiens JJ (2017). The origin of species richness patterns along environmental gradients: uniting explanations based on time, diversification rate and carrying capacity. J Biogeogr.

[22] Chown SL (2015). Biological invasions, climate change and genomics. Evol Appl.

[23] Hendry AP, Nosil P, Reiseberg LH (2007). The speed of ecological speciation. Func Ecol.

[24] Coyne JA, Orr HA (1989). Patterns of speciation in Drosophila. Evolution.

[25] Passy SI (2012). A hierarchical theory of macroecology. Ecol Lett.

[26] Heger T, Jeschke JM (2014). The enemy release hypothesis as a hierarchy of hypotheses. Oikos.

[27] Leihy RI, Duffy GA, Chown SL (2018). Species richness and turnover among indigenous and introduced plants and insects of the Southern Ocean Islands. Ecosphere.

[28] McGill BJ (2010). Towards a unification of unified theories of biodiversity. Ecol Lett.

[29] Rusek J (1998). Biodiversity of Collembola and their functional role in the ecosystem. Biodivers Conserv.

[30] Ulrich W, Fiera C (2009). Environmental correlates of species richness of European springtails (Hexapoda: Collembola). Acta Oecol.

[31] Janion-Scheepers C (2018). Basal resistance enhances warming tolerance of alien over indigenous species across latitude. PNAS.

[32] Chown, S. L. & Froneman, P. W. *The Prince Edward Islands*. *Land-sea interactions in a changing ecosystem* (Sun Press, 2008).

[33] Deharveng L (1981). Collemboles des iles subantarctiques de l’Océan Indien Mission J. Travé 1972–1973. Comité National Française des Recherches Antarctiques.

[34] Moon Katherine L., Chown Steven L., Fraser Ceridwen I. (2017). Reconsidering connectivity in the sub-Antarctic. Biological Reviews.

[35] Clarke A, Gaston KJ (2006). Climate, energy and diversity. P R Soc B.

[36] Colwell RK, Rahbek C, Gotelli NJ (2004). The mid-domain effect and species richness patterns: What have we learned so far?. Am Nat.

[37] Rahbek C (1997). The relationship among area, elevation, and regional species richness in Neotropical birds. Am Nat.

[38] Gabriel AGA (2001). Biological invasions on Southern Ocean islands: the Collembola of Marion Island as a test of generalities. Ecography.

[39] Janion C, Leinaas HP, Terblanche JS, Chown SL (2010). Trait means and reaction norms: the consequences of climate change/invasion interactions at the organism level. Evol Ecol.

[40] Burger AE (1978). Terrestrial invertebrates: a food resource for birds at Marion Island. S Afr J Antarct Res.

[41] Lee JE, Somers MJ, Chown SL (2012). Density, body size and sex ratio of an indigenous spider along an altitudinal gradient in the sub-Antarctic. Antarct Sci.

[42] Hugo, E. A. *Spatial patterns in the microarthropod community associated with Azorella selago (Apiaceae) on the sub-Antarctic Prince Edward Islands*. PhD Thesis, University of Stellenbosch, http://scholar.sun.ac.za/handle/10019.1/21743 (2006).

[43] Fjellberg, A. *Fauna Entomologica Scandinavica Volume 35. The Collembola of Fennoscandia and Denmark. Part I: Poduromorpha* (Brill, 1998).

[44] Potapov, M. Synopses on Palaearctic Collembola, volume 3, Isotomidae in *Staatliches Museum für* Naturkunde (ed. Dunger, W.) (Görlitz. 2001).

[45] Barendse J, Chown SL (2001). Abundance and seasonality of mid-altitude fellfield arthropods from Marion Island. Polar Biol.

[46] Hugo EA, McGeoch MA, Marshall DJ, Chown SL (2004). Fine scale variation in microarthropod communities inhabiting the keystone species *Azorella selago* on Marion Island. Polar Biol.

[47] Atkin OK (2015). Global variability in leaf respiration in relation to climate, plant functional types and leaf traits. New Phytol.

[48] Chown SL, Terblanche JS (2007). Physiological diversity in insects: ecological and evolutionary contexts. Adv Insect Physiol.

[49] R Development Core Team R Development Core Team. *R: A language and environment for statistical computing*. R Foundation for Statistical Computing, Vienna, Austria. ISBN 3-900051-07-0, http://www.R-project.org (2010).

[50] Deere JA, Sinclair BJ, Marshall DJ, Chown SL (2006). Phenotypic plasticity of thermal tolerances in five oribatid mite species from sub-Antarctic Marion Island. J Insect Physiol.

[51] Colwell, R. K. *EstimateS: statistical estimation of species richness and shared species from samples*, *Version 7.5*. Available at, http://viceroy.eeb.uconn.edu/EstimateS (accessed 23 February 2011) (2005).

[52] Magurran, A. E. & McGill, B. J. eds. *Biological diversity: frontiers in measurement and assessment* (Oxford University Press, 2011).

[53] McCain CM (2004). The mid-domain effect applied to elevational gradients: species richness of small mammals in Costa Rica. J Biogeogr.

[54] Crawley, M. J. *The R book*, 2^*nd*^*ed*. (John Wiley and Sons, 2013).

[55] Meiklejohn KI, Smith VR (2008). Surface areas of altitudinal zones on sub-Antarctic Marion Island. Polar Biol.

[56] le Roux PC (2013). Human activities, propagule pressure and alien plants in the sub-Antarctic: tests of generalities and evidence in support of management. Biol Conserv.

[57] Hortal J, Borges PAV, Gaspar C (2006). Evaluating the performance of species richness estimators: sensitivity to sample grain size. J Anim Ecol.

[58] Lee JE, Janion C, Marais E, Jansen van Vuuren B, Chown SL (2009). Physiological tolerances account for range limits and abundance structure in an invasive slug. P R Soc B.

[59] Overgaard J, Kearney MR, Hoffmann AA (2014). Sensitivity to thermal extremes in Australian Drosophilaimplies similar impacts of climate change on the distribution of widespread and tropical species. Glob Change Biol.

[60] Catullo RA, Ferrier S, Hoffmann AA (2015). Extending spatial modelling of climate change responses beyond the realized niche: estimating, and accommodating, physiological limits and adaptive evolution. Global Ecol Biogeogr.

[61] Honěk A (1996). Geographical variation in thermal requirements for insect development. Eur J Entomol.

[62] Dunn RR, McCain CM, Sanders NJ (2007). When does diversity fit null model predictions? Scale and range size mediate the mid-domain effect. Global Ecol Biogeogr.

[63] Field R (2009). Spatial species-richness gradients across scales: a meta-analysis. J Biogeogr.

[64] Myburgh M, Chown SL, Daniels SR, Jansen van Vuuren B (2007). Population structure, propagule pressure, and conservation biogeography in the sub-Antarctic: lessons from indigenous and invasive springtails. Divers Distrib.

[65] Stevens MI, Greenslade P, Hogg ID, Sunnucks P (2006). Southern Hemisphere springtails: could any have survived glaciation of Antarctica?. Mol Biol Evol.

[66] Lande R (1993). Risks of population extinction from demographic and environmental stochasticity and random catastrophes. Am Nat.

[67] Chown SL, Gaston KJ (2000). Areas, cradles and museums: the latitudinal gradient in species richness. Trends Ecol Evol.

[68] Slabber S, Worland MR, Leinaas HP, Chown SL (2007). Acclimation effects on thermal tolerances of springtails from sub-Antarctic Marion Island: Indigenous and invasive species. J Insect Physiol.

[69] Gaston, K. J. *The structure and dynamics of geographic ranges* (Oxford University Press, 2003).

[70] Soberón J (2007). Grinnellian and Eltonian niches and geographic distributions of species. Ecol Lett.

[71] Frenot Y (2005). Biological invasions in the Antarctic: extent, impacts and implications. Biol Rev.

[72] Hughes KA, Greenslade P, Convey P (2017). The fate of the non-native Collembolon, *Hypogastrura viatica*, at the southern extent of its introduced range in Antarctica. Polar Biol.

[73] Marquet, P. A., Fernández, M., Navarette, S. A. & Valdovinos, C. Diversity emerging: toward a deconstruction of biodiversity patterns in *Frontiers of biogeography. New directions in the geography of nature* (eds Lomolino, M. & Heaney, L.) 191–291 (Cambridge University Press, 2004).

[74] Hawkins BA (2012). Different evolutionary histories underlie congruent species richness gradients of birds and mammals. J Biogeogr.

[75] Frenot Y, Gloaguen JC, Masse L, Lebouvier M (2001). Human activities, ecosystem disturbance and plant invasions in subantarctic Crozet, Kerguelen and Amsterdam Islands. Biol Conserv.

[76] le Roux PC, McGeoch MA (2008). Changes in climate extremes, variability and signature on sub-Antarctic Marion Island. Climatic Change.

[77] Davies K, Melbourne B, McClenahan J, Tuff T (2011). Statistical models for monitoring and predicting effects of climate change and invasion on the free-living insects and a spider from sub-Antarctic Heard Island. Polar Biol.

[78] McClelland GTW (2018). Climate change leads to increasing population density and impacts of a key island invader. Ecol Appl.

[79] Chown SL (2013). Climate change and elevational diversity capacity: do weedy species take up the slack?. Biol Letters.

[80] Chown SL, Slabber S, McGeoch MA, Janion C, Leinaas HP (2007). Phenotypic plasticity mediates climate change responses among invasive and indigenous arthropods. P R Soc B.

[81] Smith VR (1987). Production and nutrient dynamics of plant communities on a sub-Antarctic Island. 1.Standing crop and primary production of Mire-grasslands. Polar Biol.

[82] Smith VR (1987). Production and nutrient dynamics of plant communities on a sub-Antarctic Island. 2.Standing crop and primary production of Fjaeldmark and Fernbrakes. Polar Biol.

