# Supplementary material for: Species-energy relationships of indigenous and invasive species may arise in different ways – a demonstration using springtails
Source: Sci Rep. 2019 Sep 24;9:13799. doi: 10.1038/s41598-019-48871-1 (PMC6760167; doi:10.1038/s41598-019-48871-1)
Supplement: Supplementary file 1 — Supplementary information [file 41598_2019_48871_MOESM1_ESM.pdf]

**Species-energy relationships of indigenous and invasive species may arise in different ways – a demonstration using springtails**

Anne M. Treasure[1*], Peter C. le Roux[1,2], Mashudu H. Mashau[1] and Steven L. Chown[3]

*[1]Centre for Invasion Biology, Department of Botany and Zoology, Stellenbosch University, Private Bag X1, Matieland 7602, South Africa*

*[2]Department of Plant and Soil Sciences, University of Pretoria, Private Bag X20, Hatfield, 0028, South Africa*

*[3]School of Biological Sciences, Monash University, Victoria 3800, Australia*

*E-mail: anne.m.treasure@gmail.com. Current address: South African Environmental Observation Network (SAEON), 8th Floor, The Towers South, Hertzog Blvd, Foreshore, Cape Town, 8001, South Africa.

## SUPPORTING INFORMATION

**Appendix S1.** Supplementary methods outlining investigations of water availability and sampling protocols for each elevational band.

**Appendix S2.** Supplementary methods for testing the dynamic equilibrium and range limitation hypotheses.

**Appendix S3.** Supplementary Results

**Online Supplement.** R script to calculate summary parameters from a time series.

## APPENDIX S1

## SUPPLEMENTARY METHODS

### Water availability

To confirm high water availability at areas other than the meteorological station where precipitation has been measured with a *ca*. 1900 mm per annum average[1], a rain gauge was installed at each of the sites at which temperature was measured on the east and west altitudinal transects on sub-Antarctic Marion Island (MI) (Fig. 1). The gauges consisted of 300 $mm^3$ PVC pipes surmounted by standard graduated funnels. They were checked and emptied at least once a month. However, the higher altitude gauges were damaged by repeated freeze-thaw cycles and, after several unsuccessful attempts to repair them, were discontinued.

### Sampling protocols for each elevational band

For each of the low elevation (10 m a.s.l., 50 m a.s.l., 200 m a.s.l.) sites, four quadrats of 2 m x 2 m each were randomly placed on average 100 m apart at the same altitude. Global Positioning System (GPS) coordinates of and plant species present in quadrats were recorded. Each quadrat was divided into 10 cm x 10 cm blocks and a core sample was collected in the centre of each of five randomly selected blocks for each quadrat using a 34 mm (diameter) stainless steel soil corer. The mid-altitude sites at 400 m a.s.l. and 600 m a.s.l. consisted of fellfield habitat, which is characterised by individuals of the cushion-forming *Azorella selago* Hook. f. separated by rocky areas[2]. At each mid-altitude site, an 80 m x 80 m quadrat was established and one core sample collected from each of 20 randomly selected *A. selago* cushions using a 34 mm stainless steel corer. Cores were taken from the southern sides of the plants (< 20 cm diameter) as higher abundances of microarthropods have been found on these sides as opposed to other areas of *A. selago* cushions[3]. Cores were taken midway between the

centre of the cushion and the cushion edge. For all of these sites each core sample was placed in an individually labelled plastic bag and processed on the same day of collection.

For each of the high altitude polar desert sites (750 m a.s.l., 850 m a.s.l., 1000 m a.s.l.), soil samples at each site were collected along four 25 m transects, which were demarcated horizontally across the slope at least 50 m apart. Samples were collected at 5 m intervals along each transect, resulting in a total of 20 samples per site. The same area was never sampled twice, with samples from the second sampling period being interspersed between those from the first sampling period. For each sample, the top 5 cm of soil and stones was removed from a 10 cm x 10 cm area because core sampling is not effective and the two methods used this way yield comparable results[4].

## REFERENCES

1. Chown, S. L. & Froneman, P. W. *The Prince Edward Islands. Land-sea interactions in a changing ecosystem* (Sun Press, 2008).
2. Gremmen, N. J. M. *The Vegetation of the sub-Antarctic Islands Marion and Prince Edward* (Junk, 1981).
3. Hugo, E.A., McGeoch, M.A., Marshall, D.J. & Chown, S.L. 2004. Fine scale variation in microarthropod communities inhabiting the keystone species *Azorella selago* on Marion Island. *Polar Biol* **27**, 466-473.
4. Gabriel, A.G.A. *et al*. Biological invasions on Southern Ocean islands: the Collembola of Marion Island as a test of generalities. *Ecography* **24**, 421-430 (2001).

**APPENDIX S2**

*Dynamic Equilibrium Explanation*

The dynamic equilibrium explanation posits that elevated energy enables populations to recover faster from disturbance[1]. The explanation thus requires demographic information about populations such as growth rates or abundance, which can be compared to disturbance events. On MI, springtails are found island-wide and large-scale disturbances are mostly in the form of occasional low temperature events[2]. Landslides are uncommon, the island is largely volcanically inactive (recent activity has all been very localized[3]), and indigenous land mammals and birds (apart from the Lesser Sheathbill (*Chionis minor marionensis* Reichenow), which does not feed on springtails[4]) are absent (see Chown and Froneman[3] for a review). Therefore, in this study, we hypothesised that if this explanation was appropriate, the abundance of the springtail species would be correlated with disturbances that occur when soil temperatures cross two different thresholds, namely 0°C (the freezing point of water), and the mean lower development threshold (LDT) of the springtails, a temperature at which development ceases. We did not consider the supercooling point (SCP) of the springtails (i.e. the temperature at which they freeze and die, see Slabber et al.[5]) because environmental temperatures did not drop below mean SCP values recorded for any of the species[5,6]. The rationale for our approach is that if a springtail population is not resilient to the crossing of such thresholds, its abundance would be lower in areas where more of these disturbances occur compared with those areas with fewer disturbances. It was hypothesised that both the number of times the thresholds were crossed and the maximum amount of time spent below the thresholds at any one time (hereafter called longest duration) are important disturbance events. Substantial information exists on the thermal biology of the springtail populations on Marion Island, which was used to obtain SCP and LDT values[6,7,C. Janion unpublished data]. The number of times that temperatures dropped below mean SCP and LDT, and the longest

duration that temperatures remained below mean SCP and LDT were calculated for the indigenous, invasive and combined groups of springtails present at each site. Mean values were calculated for these springtail groups[6].

The short-term and long-term temperature datasets were used for calculating the number of times that temperature dropped below, or the longest duration below, these thresholds. To account for differences in the elevation of the dataloggers in the short- and long-term soil temperature data, the adiabatic lapse rate in soil temperature was calculated from the long-term soil temperature data (-5.83°C per 1000 m). Temperature data at the nearest comparable (long-term) location were then adjusted by adding (or subtracting) a constant value, calculated as the product of the lapse rate coefficient and the altitudinal difference between the long- and short-term data logging sites. Analysis of the short-term eastern versus western temperature data indicated only slight differences between the two sides of the island at the elevations sampled (see below) and therefore the long-term data from the eastern side of the island was considered sufficient to use for the western side of the island.

The relationships between raw transformed abundance of indigenous and invasive springtails, and events below 0ºC, longest duration below 0ºC, events below LDT and longest duration below LDT, were investigated using generalised linear models (in R3.1.0, Quasipoisson distribution, log link function) for the short-term and the long-term temperature data set.

*Range Limitation*

This explanation predicts that species occur in areas where they can meet their physiological requirements[1], and assumes that more species can tolerate warmer climatic conditions[8]. To examine this explanation and to distinguish possible outcomes from the dynamic equilibrium

explanation, abundance of indigenous, invasive and combined species groups were compared to the minimum and maximum temperatures recorded at each site (i.e. winter mean daily minimum and summer mean daily maximum), as well as the number of generations possible at each elevation. Much evidence exists that ectotherm distributions can be limited either directly by minimum or maximum temperatures, or indirectly as a consequence of inadequate growing season length[9,10]. Both the short-term and long-term temperature datasets were used (see above). The number of generations possible was calculated using the sum of effective temperatures (SET), which is the number of day degrees (dd) heat units that are required above the LDT for an individual to complete development[7].

Mean SET values were calculated for the indigenous, invasive and combined groups of species present at each site. When soil temperature exceeded LDT thresholds the difference was calculated and divided by the time interval between subsequent records (usually 1 hour) to estimate the degree hours accumulated by individuals experiencing those conditions. Mean developmental rate was then calculated from the mean proportion of the SET that each temperature recording contributed. Multiplying this rate by the number of days within each time period estimated the number of generations possible. This was calculated for the indigenous, invasive and combined groups of species at each site.

The effects of minimum and maximum temperatures and generations possible on the raw abundance of indigenous and invasive species were investigated using generalised linear models (as above).

## REFERENCES

1. Evans, K.L., Warren, P.H. & Gaston, K.J. Species-energy relationships at the macroecological scale: a review of the mechanisms. *Biol Rev* **80**, 1-25 (2005).

2. Lee, J.E., Janion, C., Marais, E., Jansen van Vuuren, B. & Chown, S.L. Physiological tolerances account for range limits and abundance structure in an invasive slug. *P R Soc B* **276**, 1459-1468 (2009).
3. Chown, S.L. & Froneman, P.W. *The Prince Edward Islands. Land-sea interactions in a changing ecosystem* (Sun Press, 2008).
4. Burger, A.E. Terrestrial invertebrates: a food resource for birds at Marion Island. *S Afr J Antarct Res* **8**, 87-99 (1978).
5. Slabber, S., Worland, M.R., Leinaas, H.P. & Chown, S.L. Acclimation effects on thermal tolerances of springtails from sub-Antarctic Marion Island: indigenous and invasive species. *J Insect Physiol* **53**, 113-125 (2007).
6. Janion, C., Worland, M.R. & Chown, S.L. Assemblage level variation in springtail lower lethal temperature: the role of invasive species on sub-Antarctic Marion Island. *Physiol Entomol* **34**, 284-291 (2009).
7. Janion, C., Leinaas, H.P., Terblanche, J.S. & Chown, S.L. Trait means and reaction norms: the consequences of climate change/invasion interactions at the organism level. *Evol Ecol* **24**, 1365-1380 (2010).
8. Kerr, J.T., Vincent, R. & Currie, D.J. Lepidopteran richness patterns in North America. *Ecoscience* **5**, 448-553 (1998).
9. Krushelnycky, P.D., Joe, S.M., Medeiros, A.C., Daehler, C.C. & Loope, L.L. The role of abiotic conditions in shaping the long-term patterns of high-elevation Argentine ant invasion. *Divers Distrib* **11**, 319-331 (2005).
10. Chown, S.L. & Gaston, K.J. Macrophysiology for a changing world. *P R Soc B* **275**: 1469-1478 (2008).

**Table S1** The mean lower development threshold (LDT) of indigenous and invasive springtail eggs calculated from egg development rates given in Janion et al. (2010). Empty cells represent the absence of invasive species at two higher elevation sites.

| Site & altitude (m a.s.l.) | Mean LDT Indigenous (°C) | Mean LDT Invasive (°C) |
|---|---|---|
| **East** | | |
| 10 | 2.24 | 2.61 |
| 50 | 0.18 | 2.69 |
| 200 | 0.18 | 2.69 |
| 400 | 1.20 | 2.38 |
| 600 | 0.18 | 2.45 |
| 750 | -0.87 | 2.31 |
| 850 | -0.87 | 2.31 |
| 1000 | -0.87 | |
| **West** | | |
| 10 | 1.20 | 2.61 |
| 50 | 1.20 | 2.61 |
| 200 | 0.18 | 2.76 |
| 400 | 1.20 | 2.69 |
| 600 | 0.18 | 2.61 |
| 750 | -0.87 | |
| 850 | -0.87 | 2.31 |
| 1000 | -0.87 | 2.31 |

## APPENDIX S3 Supplementary Results

**Table S2** Summary of soil temperatures recorded at the altitudinal sites of the present study for winter and summer months (m a.s.l. = metres above sea level).

| | Winter (mid May-08 to mid Aug-08) (°C) | | | | | | | Summer (mid Nov-08 to mid Feb-09) (°C) | | | | | | |
|---|---|---|---|---|---|---|---|---|---|---|---|---|---|---|
| Site & altitude (m a.s.l.) | *n* | Mean | Absolute min | Absolute max | Range | Mean daily min | Mean daily max | *n* | Mean | Absolute min | Absolute max | Range | Mean daily min | Mean daily max |
| **East** | | | | | | | | | | | | | | |
| 10 | 3561 | 4.94 | 1.12 | 9.66 | 8.54 | 4.04 | 5.74 | 2652 | 8.27 | 2.00 | 18.00 | 16.00 | 6.90 | 10.36 |
| 50 | 3559 | 4.93 | 2.09 | 9.64 | 7.55 | 4.39 | 5.28 | 2654 | 7.20 | 5.00 | 18.69 | 13.69 | 6.64 | 8.11 |
| 200 | 3558 | 4.07 | 1.11 | 8.67 | 7.56 | 3.50 | 4.39 | 4750 | 7.89 | 4.13 | 12.67 | 8.54 | 6.94 | 9.01 |
| 400 | 3557 | 3.33 | -0.89 | 16.66 | 17.55 | 2.11 | 4.32 | 4754 | 6.40 | 1.08 | 16.12 | 15.04 | 4.03 | 9.18 |
| 600 | 3557 | 2.80 | 0.57 | 10.63 | 10.06 | 1.97 | 3.38 | 3492 | 5.08 | 0.00 | 21.50 | 21.50 | 3.11 | 8.66 |
| 750 | 3530 | 2.21 | -1.97 | 11.16 | 13.13 | 1.15 | 2.94 | 4751 | 4.44 | 0.05 | 20.62 | 20.57 | 2.54 | 7.02 |
| 850 | 3526 | 1.83 | -0.39 | 10.15 | 10.54 | 0.99 | 2.31 | 3494 | 2.69 | -0.91 | 16.69 | 17.60 | 1.35 | 5.38 |
| 1000 | 3641 | 1.47 | -1.39 | 10.16 | 11.55 | 0.78 | 1.92 | 4745 | 1.96 | -0.97 | 14.22 | 15.19 | 1.09 | 3.04 |
| **West** | | | | | | | | | | | | | | |
| 10 | 3656 | 5.42 | 1.60 | 9.16 | 7.56 | 4.68 | 5.81 | 4751 | 8.17 | 3.64 | 14.70 | 11.06 | 7.28 | 9.28 |
| 50 | 3674 | 5.11 | 2.12 | 7.64 | 5.52 | 4.61 | 5.31 | 4751 | 7.09 | 4.17 | 11.71 | 7.54 | 6.44 | 7.86 |
| 200 | 3655 | 4.66 | 1.09 | 9.14 | 8.05 | 3.95 | 5.16 | 4750 | 6.98 | 3.63 | 13.17 | 9.54 | 6.30 | 7.92 |
| 400 | 3651 | 3.23 | 0.10 | 8.59 | 8.49 | 2.36 | 3.64 | 4751 | 5.36 | 1.58 | 14.14 | 12.56 | 4.24 | 6.56 |
| 600 | 3634 | 2.90 | 0.07 | 9.61 | 9.54 | 2.03 | 3.43 | 3923 | 3.25 | 0.00 | 13.50 | 13.50 | 2.43 | 4.73 |
| 750 | 3632 | 2.28 | -0.42 | 9.66 | 10.08 | 1.47 | 2.78 | 2374 | 2.27 | -1.50 | 15.00 | 16.50 | 1.06 | 3.74 |
| 850 | 3629 | 1.58 | -0.91 | 8.19 | 9.10 | 0.78 | 2.07 | 4750 | 2.14 | 0.07 | 10.17 | 10.10 | 1.46 | 2.97 |
| 1000 | 3625 | 1.01 | -2.38 | 8.69 | 11.07 | 0.24 | 1.32 | 3924 | 1.18 | -1.50 | 15.00 | 16.50 | 0.48 | 2.51 |

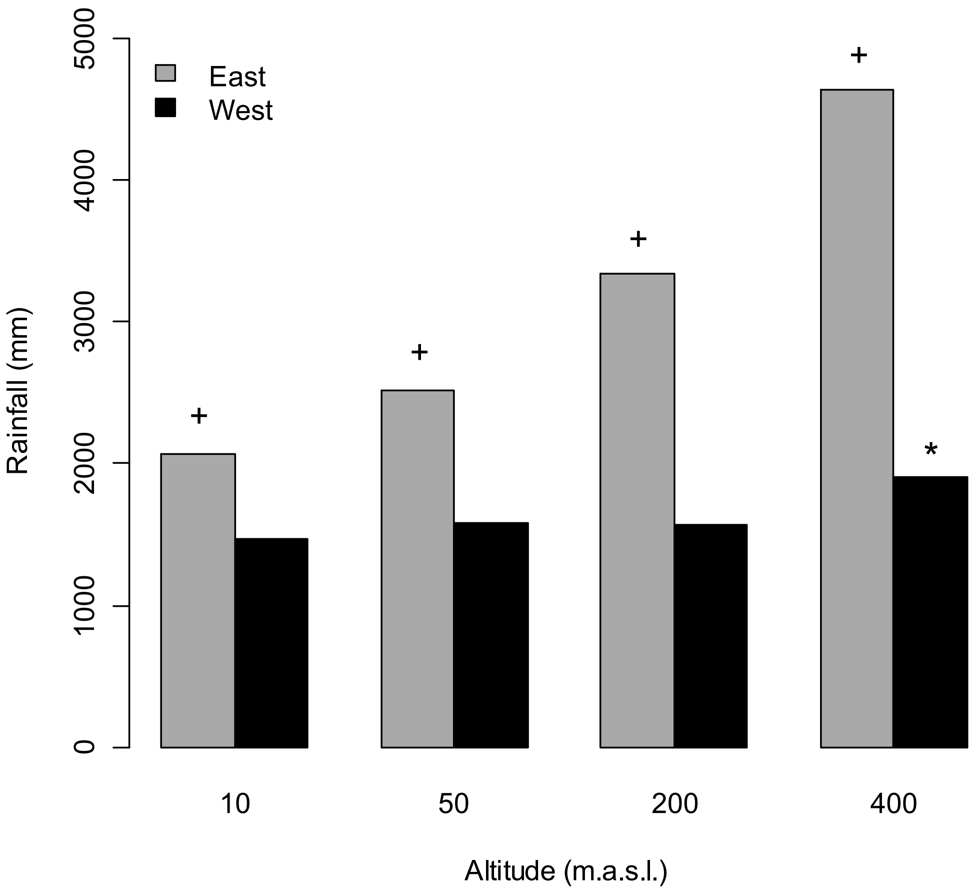


**Figure S1** Annual rainfall (May 2008 to April 2009) recorded at the four lower elevations along the east and west transects on Marion Island (+ = these gauges were found to be overflowing in at least one month and rainfall estimates are therefore conservative and minimum amounts; * = estimated rainfall as the west 400 m a.s.l. rain gauge was damaged by ice and discontinued; m a.s.l. = metres above sea level).

(a)

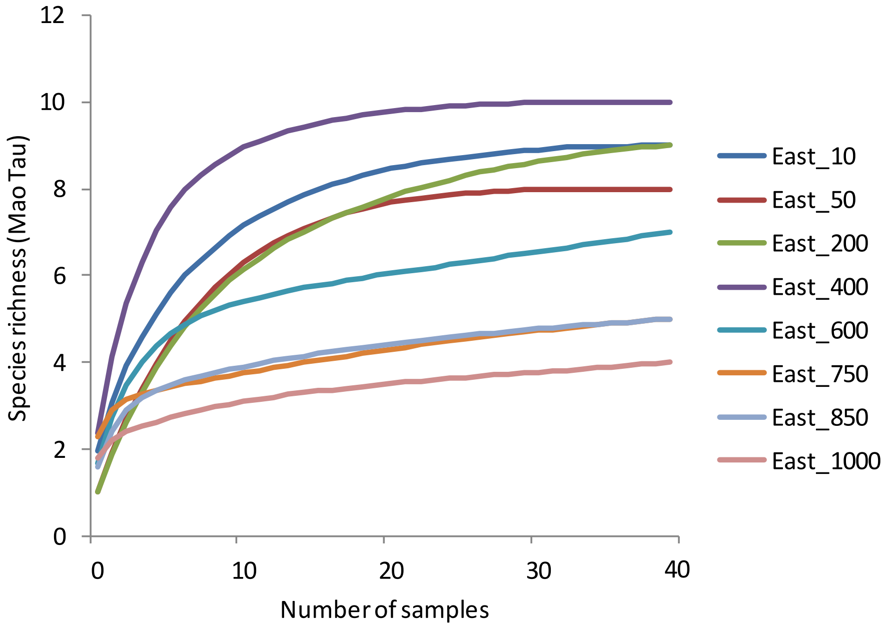


(b)

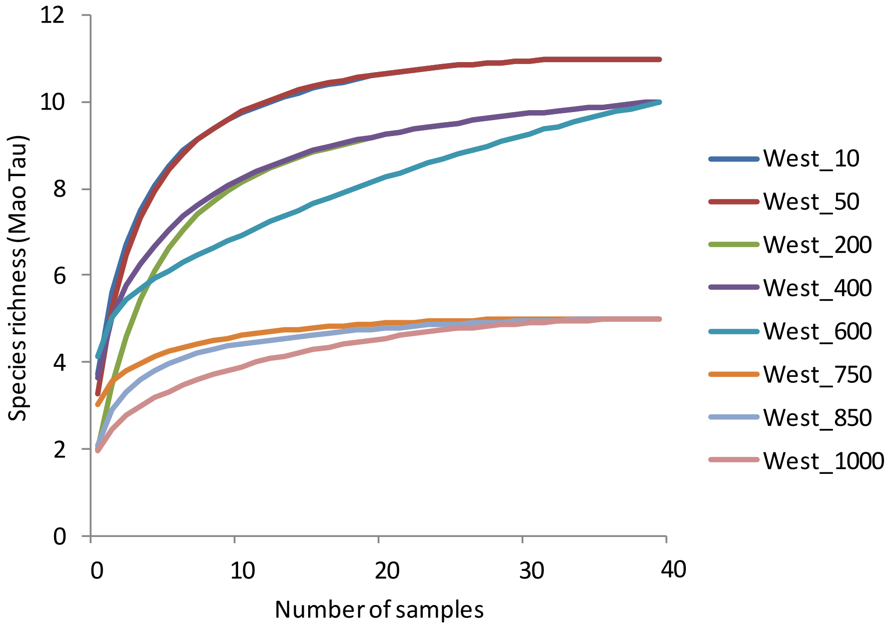


**Figure S2** Rarefaction curves for eight elevations on (a) the east transect and (b) the west transect on Marion Island standardised by sample effort of springtails.

**Table S3** Abundance, observed species richness ($S_{obs}$) and estimated species richness (Jacknife2) of indigenous and invasive springtail species along two altitudinal gradients on Marion Island. Altitude in metres above sea level.

| Transect | Altitude (m.a.s.l.) | Species group | Abundance (individuals $m^{-2}$) | $S_{obs}$ | Jacknife2 |
|---|---|---|---|---|---|
| East | 10 | Indigenous | 361.36 | 6 | 4.1 |
| East | 50 | Indigenous | 120.45 | 5 | 5.0 |
| East | 200 | Indigenous | 205.94 | 6 | 4.1 |
| East | 400 | Indigenous | 788.78 | 7 | 7.0 |
| East | 600 | Indigenous | 582.84 | 5 | 5.0 |
| East | 750 | Indigenous | 88.75 | 4 | 3.1 |
| East | 850 | Indigenous | 35.31 | 4 | 5.9 |
| East | 1000 | Indigenous | 67.81 | 4 | 5.9 |
| East | 10 | Invasive | 9916.10 | 3 | 3.0 |
| East | 50 | Invasive | 442.96 | 3 | 3.0 |
| East | 200 | Invasive | 93.25 | 3 | 4.0 |
| East | 400 | Invasive | 567.30 | 3 | 3.0 |
| East | 600 | Invasive | 15.54 | 2 | 5.9 |
| East | 750 | Invasive | 0.63 | 1 | 2.9 |
| East | 850 | Invasive | 1.88 | 1 | 1.0 |
| East | 1000 | Invasive | 0.00 | 0 | 0.0 |
| West | 10 | Indigenous | 2207.03 | 7 | 6.1 |
| West | 50 | Indigenous | 951.98 | 7 | 6.1 |
| West | 200 | Indigenous | 411.88 | 7 | 8.0 |
| West | 400 | Indigenous | 1729.10 | 7 | 7.0 |
| West | 600 | Indigenous | 4188.70 | 6 | 7.9 |
| West | 750 | Indigenous | 215.63 | 5 | 5.0 |
| West | 850 | Indigenous | 109.38 | 4 | 4.0 |
| West | 1000 | Indigenous | 96.25 | 4 | 3.1 |
| West | 10 | Invasive | 20026.48 | 4 | 4.0 |
| West | 50 | Invasive | 1095.74 | 4 | 4.0 |
| West | 200 | Invasive | 932.55 | 3 | 3.0 |
| West | 400 | Invasive | 132.11 | 3 | 4.0 |
| West | 600 | Invasive | 69.94 | 4 | 6.9 |
| West | 750 | Invasive | 0.00 | 0 | 0.0 |
| West | 850 | Invasive | 1.25 | 1 | 0.1 |
| West | 1000 | Invasive | 1.25 | 1 | 0.1 |

**Table S4** Results for the generalised estimating equation models (GEE) for the relationships between raw (Sobs) and estimated (Jacknife2) species richness, mean temperature and log transformed surface area of altitudinal bands.

| **GEE** | Residual df | DE | Adj. $R^2$ | Coefficient | *p* |
|---|---|---|---|---|---|
| Response: Indigenous $S_{obs}$ | | | | | |
| log(area) | 14 | 0.55 | 0.53 | 0.607 | < 0.001 |
| mean temperature | 14 | 0.58 | 0.56 | 0.091 | < 0.001 |
| log(area) + mean temperature | 13 | 0.64 | 0.61 | | |
| log(area) | | | | 0.309 | 0.157 |
| mean temperature | | | | 0.056 | 0.082 |
| Response: Invasive $S_{obs}$ | | | | | |
| log(area) | 14 | 0.38 | 0.45 | 1.489 | 0.004 |
| mean temperature | 14 | 0.50 | 0.59 | 0.277 | <0.001 |
| log(area) + mean temperature | 13 | 0.52 | 0.61 | | |
| log(area) | | | | 0.454 | 0.527 |
| mean temperature | | | | 0.228 | 0.038 |
| Response: Indigenous Jacknife2 | | | | | |
| log(area) | 14 | 0.17 | 0.12 | 0.351 | 0.163 |
| mean temperature | 14 | 0.11 | 0.05 | 0.035 | 0.362 |
| log(area) + mean temperature | 13 | 0.18 | 0.13 | | |
| log(area) | | | | 0.433 | 0.309 |
| mean temperature | | | | -0.015 | 0.807 |
| Response: Invasive Jacknife2 | | | | | |
| log(area) | 14 | 0.17 | 0.14 | 0.801 | 0.305 |
| mean temperature | 14 | 0.29 | 0.22 | 0.227 | 0.046 |
| log(area) + mean temperature | 13 | 0.29 | 0.22 | | |

| | | |
|---|---|---|
| log(area) | -0.176 | 0.876 |
| mean temperature | 0.246 | 0.146 |

DE = % deviance explained

# updated 6 Aug 2019

```
##################################################################
## R code to calculate summary parameters from a time series. ##
##################################################################
```

# This code is designed to calculate a variety of potentially biologically relevant parameters from a time series. While the code has been written with a temperature time series in mind, it could also be applied to other time series (with minor modifications required for figure axes, etc.).

# When using this code, please cite Treasure et al. (2019; DOI: 10.1038/s41598-019-48871-1).

# Further information about this code can be requested via email from Peter le Roux (peter.c.leroux@gmail.com). If you improve the performance of this code or extend its functionality, please consider sending us a copy at the above email address.

# This code was originally written in R 2.9 and R 2.10, and has been tested in R 3.1.0 (available from http://cran.r-project.org/). For users unfamiliar with R, please consult "An Introduction to R" (available from http://cran.r-project.org/doc/manuals/R-intro.pdf) for the basics.

# The code will calculate standard metrics (e.g. mean, SD, SE, minimum, maximum), but will also calculate threshold-related metrics (e.g. occurrences, total duration and maximum duration above or below a specified threshold, including degree days) and temporal autocorrelation. These calculations can be calculated for daily, monthly and/or annual periods, with the additional option of splitting calculations into day vs. night periods.

# Additionally, where response curves are known (e.g. relationship between activity and temperature), cumulative responses can be calculated (i.e. integrated) over the time series. This code currently allows three different response curves to be integrated in a single analysis.

# Further, where thermal requirements (degree day requirements / sum of effective temperatures) and developmental thresholds (upper and/or lower developmental thresholds) are provided, estimated generation times and potential numbers of generations per year will be calculated.

# Finally, a diversity of graphs are created to assist the user in examining the raw data and any interpolated data, as well as visualizing the summary metrics calculated.

# A variety of abreviations are used throughout the code - the most common abbreviation is "TTS" which stands for "temperature time series" and simply refers to the raw data provided by the user.

# Please note that when calculating parameters related to thresholds (e.g. duration below a threshold), if analyses are being repeated per year, there must

be more than one record per year. Similarly, when these calculations are being repeated per month, there must be more than one record per month. Having just a single data point per year or month will cause an error in the code and result in all calculations being aborted.

```
# To calculate spatial autocorrelation, the package spdep{} must be installed (available from http://cran.r-project.org/web/packages/spdep/index.html).
library(spdep)


## Data format
###############

# For simplicity the first six columns of data files should contain (separately) values for the year, month, day, hour, minute and second of each record (with the columns named "Year", "Month", "Day", "Houe", "Minute", "Second" for simplicity). However, an option exist for providing date-time information in one column and specifying its exact format.
# Further, since none of the columns should be named "Temperature" as this term is used as a variable in the code - any columns labled as "Temperature" will automatically be renamed as "Tmprtr".

# Data can be provided in tab-delimited text files or comma-separated value format.


###################################
# 1. Set input and output details #
###################################

# Set the directory that output files should be created in
setwd("C:\\Pete\\UP_research\\SANAP_2018\\Climate_analysis")

# Provide the number of each column(s) to be analysed. Usually starts from 7, since first six columns are typically date and time values. More than one column can be specified here if multiple variables are to be analysed sequentially (in such a case please use the format "c(7, 8, 9)").
TimeSeriesColumn <- c(7:9)

# Provide the prefix for all output files to be creates (all outputs are provided in comma separated values format, and therefore gain a ".csv" suffix). Even if multiple variables are being analysed sequentially, all results will be output into one file and therefore only one output file name is required.

OutputFileName <- "Prelim_5Aug"

# Number the different sites (or time series variables) in the dataset here. This provides a unique identifier for each time series variable. Values must be numeric, since text values will affect output matrices. Sites (or the other time series variables being analysed) must be numbered in the same order as their columns are selected.
Site <- c(1:3)
```

```
# Names for each site can be provided here(optional; default is NULL). If names
are provided, they are outputted as text file. If multiple sites (or other
variables) are being analysed, names should be provided for each, all within
quotation marks
SiteNames <- c("MaxTemp", "MinTemp", "Ppt")

# Provide the latitude of each site (south = negative values; provided in
decimal degrees), in the same order as the sites are numbered. This is used to
calculate sunrise & sunset times for splitting data into day and night
Lat <- rep(-46.88, 3)

# Provide the longitude of each site (east = negative values)
Long <- rep(-37.73, 3)

# Provide the time zone of each site (relative to GMT; given in hours)
TimeZone <- rep(-2, 3)

# Define the number of datafiles to be combined before analysis. If multiple
data files are to be loaded and merged, the number of data files should be
specified here. This option is provided for data outputted from old versions of
Excel (where there was a limit on the number of rows in the dataset). File need
to be loaded in the correct order (oldest records first, newest last).
NumberFiles <- 1



##########################################################
# 2. Choose outputs required and set analysis parameters #
# Indicate with a "y" for yes, "n" for no                #
##########################################################


# 2a. Define the characteristics of the input file
##################################################

# set the format of the input file - alternatives are text files ("txt") or
comma-separated values ("csv"). These parameters must always be specified in
quotation marks.
FileFormat = "csv"

# is the data arranged with date & time in six columns (i.e. Year, Month, Day,
Hour, Minute, Second)? If not "y", then date-time data must be in one column and
user must be sure to set the correct DateTime.format [below].
DateTime.in.six.columns <- "y"

# if the data's date and time stamps are not already split into six colulmns,
then you need to specify format of DateTime column (Enter "?strptime" in R for
full details about formatting codes)
DateTime.format <- "%Y/%m/%d %H:%M"
```

```
# 2b. Define constants relevant to missing data and interpolation of time series
data
#################################################################################
#####

# Here users can specify is interpolations should be performed for missing data,
and , if so, what specific method should be used. The options are for simple
linear interpolation or sinusoidal interpolation (where the daily temperature
cycle is estimated from the temperature trends before and after the missing
data). Further, the user can specify a threshold duration below which
interpolation should be avoided (termed "short gaps" here), but which will
trigger interpolation when exceeded ("long gaps").

# First, define the maximum duration of missing data which can be ignored (in
hours). Longer periods of missing data are either interpolated, or considered
true breaks in time series (see the comment for the next line of code). A
sensible guideline is to set this as at least 2 x sampling frequency (i.e. for
hourly recordings, gap must be > 2 hours), as this means that one missing data
point does not trigger interpolation.
ShortGap <- 25

# Next, define the minimum duration of missing data (in hours) which is
considered a "true break" in the time series (i.e. too long to interpolate
across = "long gap"). The option exists to calculate parameters for each segment
of a time series that contains such breaks separately.
LongGap <- (24 * 10)

# Thus interpolation will be performed for all sections of missing data where
the gap in the time series is longer than "ShortGap" and shorter than "Long Gap"

### Defining which interpolation method should be applied. Multiple
interpolation methods can be applied, with different parameters calculated from
the different interpolated time series.
# Should a linear interpolation be applied across all gaps (i.e. estimating
missing data for all periods exceeding ShortGap)?
Do.Interpolation <- "y"

# Should a linear interpolation be applied across gaps in time series that are
less than "LongGap" hours? (i.e. LongGaps are not interpolated)
Do.Interpolation.Across.Short.Gaps <- "y"

# Should a sinusoidal function (i.e. estimating daily thermal trends) be applied
to interpolate across all gaps in time series (i.e. estimating missing data for
all periods exceeding ShortGap)?
Do.Sinusoidal.Interpolation <- "n"

# Define the number of records [NOT hours] that should be averaged to determine
the start and end point for interpolation of data across missing data
RecordsForInterpolation <- (4)

### Defining if time series with missing data should be analysed as one time
series or as multiple time series (i.e. each segment of the time series analysed
```

```
separately)
# Should each segment be analysed separately? (segments = portions of time
series separated by missing data longer than "LongGap")?
Do.Analyses.Each.Segment <- "n"

# If "Do.Analyses.Each.Segment = TRUE", then should segments be split by times
exceeding "ShortGap"?
Do.Analyses.Each.Segment.No.Interpolation <- "n"

# If "Do.Analyses.Each.Segment = TRUE", then should segments be split by times
exceeding "LongGap" (short gaps filled by linear interpolation)?
Do.Analyses.Each.Segment.With.Interpolation <- "n"



# 2c. Define constants relevant to data summaries
##################################################

# Define the minimum number of records per 24 hours for that 24 hour period to
be included in analysis. This value may be set to zero if required.
MinDailyRecords <- 1

# Define the minimum number of records per day or night for day (or night) to be
included in analysis. This value may be set to zero if required.
MinDNRecords <- 4

# Define the percentile over which to calculate extreme values (i.e. extreme
events). Note that the value "1 - Quantile" will be also calculated for the
corresponding lower (or higher) threshold. For example, if set to 0.95, then the
highest and lowest 5% of values will be identified as extreme values.
Quantile <- 0.95

# Summary tables - these will be calculated, but the user should choose if an
output file is created
Do.DatasetOutput <- "n"      # summarizes the data contained in the entire
dataset
Do.YearlyOutput <- "y"       # summarizes the data per year
Do.MonthlyOutput <- "n"      # summarizes the data per month and year
Do.DailyOutput <- "n"        # summarizes the data per day, month and year
Do.MonthlySummary <- "y"     # summarizes the data per month (i.e. to examine
seasonal trends, irrespective of year)
Do.Plot <- "y"                  # plots four summary figures

# Should summary tables should also be created and outputted that split the time
series by day and night? This could be useful, for example, when comparing
conditions experienced by diurnal and nocturnal species.
Do.DayNightOutput <- "n"



# 2d. Define constants relevant to temporal autocorrelation analyses
####################################################################
```

```
# Define if temporal lags should be measured in "days" or "hours"
lag.interval <- "days"

# Define the maximum lag over which temporal autocorrelation should be measured
(units defined in previous line)
lag.max <- 14

# Should the temporal autocorrelation analyses use all readings per day?
UseAllReadingsForACF <- "y"

# If UseAllReadingsForACF = "n", then which hours should be used to calculate
temporal autocorrelation? Multiple values may be inputted here. This option
allows used to avoid measuring hour-to-hour autocorrelation, and to rather focus
on autocorrelation on the scale of days
HoursToUse <- c(12)

# if UseAllReadingsForACF = "n", then which minutes should be used to calculate
temporal autocorrelation? To account for datasets with more than one reading per
hour.
MinutesToUse <- c(0 : 59)   # the dafault "c(0 : 59)" assumes only one reading
her hour

# should temporal autocorrelation analyses be performed (across the whole
dataset, each year and each month, respectively), and should the resulting
autocorrelograms be plotted?
Do.DatasetAutocorrel = "n"
Do.YearlyAutocorrel = "y"
Do.MonthlyAutocorrel = "n"
Plot.DatasetAutocorrel = "n"
Plot.YearlyAutocorrel = "y"
Plot.MonthlyAutocorrel = "n"



# 2e. Define constants relevant to calculations of durations between thresholds
################################################################################

# Should analyses of durations above, below or between thresholds be run?
Do.Thresholds <- "y"

### Define if interpolated data should be used to calculate the duration between
thresholds. This is an important consideration in time series with much missing
data, as the interpolations are likely to underestimate the duration above or
below extreme thresholds.
# Should these calculations be performed on the unmodified time series (i.e.
before any interpolations are performed)?
Thresholds.Use.TTS <- "n"

# Should the calculations only be performed on the longest time series segment
without gaps in time series? This is one option for avoiding calculating the
duration above, below or between thresholds on interpolated data
```

```
Thresholds.Use.LongestDuration <- "n"

# Should calculations be performed on the time series after all missing data are
linearly interpolated?
Thresholds.Use.Interpolated.TTS <- "y"

# Should calculations be performed on the time series after all missing data are
interpolated using a sinusoidal function?
Thresholds.Use.Interpolated.Sinus.TTS <- "n"

# Should calculations be performed on the longest time series segment after all
short gaps of missing data are linearly interpolated (i.e. on the longest
segment that does not have missing data exceeding LongGap)?
Thresholds.Use.Interpolated.LongestDuration <- "n"

# Define pairs of threshold values here - first the lower threshold, then the
upper threshold for each pair of threshold values.
# setting the lower threshold to "-Inf" causes the calculation to determine the
duration below the upper threshold
# setting the upper threshold to "Inf" causes the calculation to determine the
duration above the lower threshold
Thresholds <- c(-Inf, 0, 10, Inf, -Inf, 0.001, -Inf, 1)       # This illustrates
how to enter data for two pairs of thresholds.

# Provide a name for each pair of threshold values (to be used in the output
files)
Threshold.names <- c("subzero", ">10C", "true drought", "drought<1mm")

# Across what periods should these calculations be reported? For the entire
dataset, or per year, or per month and year?
Do.DatasetBtwnOutput <- "n"       # summarizes the data contained in the entire
dataset
Do.YearlyBtwnOutput <- "y"        # summarizes the data per year
Do.MonthlyBtwnOutput <- "n"       # summarizes the data per month and year


# Define functions for temperature-response curves
##################################################

# Here users can specify up to three different response functions, which will be
integrated over the course of the time series. While stepped functions are
illustrated here, continuous functions could also be analysed when defined as a
function of the variable "temperature" (enter "help("function") into R for more
details).

# function 1 based on Witt & Giliomee (1999) Soil-surface temperatures at which
six species of ants (Hymenoptera: Formicidae) are active. – African Entomology
7: 161-164.
TemperatureResponse1 <- function(temperature) {
        if (temperature < 5) {response.factor = 0}
        if (temperature >= 5  & temperature < 10) {response.factor = 0.047}
        if (temperature >= 10 & temperature < 15) {response.factor = 0.307}
```

```
        if (temperature >= 15 & temperature < 20) {response.factor = 0.441}
        if (temperature >= 20 & temperature < 25) {response.factor = 0.093}
        if (temperature >= 25 & temperature < 30) {response.factor = 0.045}
        if (temperature >= 30 & temperature < 35) {response.factor = 0.016}
        if (temperature >= 35 & temperature < 40) {response.factor = 0.042}
        if (temperature >= 40 & temperature < 45) {response.factor = 0.009}
        if (temperature >= 45 ) {response.factor = 0}
        response.factor}

# function 2 based on results of Abril et al. (2007) Foraging activity and
dietary spectrum of the Argentine ant (Hymenoptera: Formicidae) in invaded
natural areas of the northeast Iberian Peninsula. – Environmental Entomology 36:
1166-1173.
TemperatureResponse2 <- function(temperature) {
        if (temperature < 5) {response.factor = 0}
        if (temperature >= 5  & temperature < 10) {response.factor = 0.017}
        if (temperature >= 10 & temperature < 15) {response.factor = 0.061}
        if (temperature >= 15 & temperature < 20) {response.factor = 0.278}
        if (temperature >= 20 & temperature < 25) {response.factor = 0.221}
        if (temperature >= 25 & temperature < 30) {response.factor = 0.220}
        if (temperature >= 30 & temperature < 35) {response.factor = 0.163}
        if (temperature >= 35 & temperature < 40) {response.factor = 0.040}
        if (temperature >= 40 & temperature < 45) {response.factor = 0}
        if (temperature >= 45 ) {response.factor = 0}
        response.factor}

# function 3 based on results of Markin (1970) Foraging behavior of the
Argentine ant in a California citrus grove. – Journal of Economic Entomology 63:
740–744.
TemperatureResponse3 <- function(temperature) {
        if (temperature < 5) {response.factor = 0}
        if (temperature >= 5  & temperature < 10) {response.factor = 0.063}
        if (temperature >= 10 & temperature < 15) {response.factor = 0.126}
        if (temperature >= 15 & temperature < 20) {response.factor = 0.144}
        if (temperature >= 20 & temperature < 25) {response.factor = 0.191}
        if (temperature >= 25 & temperature < 30) {response.factor = 0.215}
        if (temperature >= 30 & temperature < 35) {response.factor = 0.221}
        if (temperature >= 35 & temperature < 40) {response.factor = 0.040}
        if (temperature >= 40 & temperature < 45) {response.factor = 0}
        if (temperature >= 45 ) {response.factor = 0}
        response.factor}


# 2g. Define constants relevant to developmental duration and generations
produced per year
################################################################################
###########

# Should developlmental times be calculated?
Do.DevelopmentalTimes <- "y"

# Define lower developmental threshold temperatures. A value of "-Inf" can be
```

```
used if the lower developmental threshold is not being considered. The example
here provides the LDT for two different species
LDT <- c(8.25, 7.6, 4.68, 4.85)

# Define total cumulative DEGREE HOURS [NB = hours] (i.e. sum of effective
temperatures) required for development. SET is specified here for two different
species
SET <- c(270 * 24, 688 * 24, 244 * 24, 102 * 24)

# Provide a name for each set of LDT and SET values (this will be used to label
the outputs).
SET.spp <- c("Plutella_xylostella", "Agrotis_ipsilon", "Aphidius_matricariae",
"Rhopalosiphum_padi")

# Provide labels for each set of LDT and SET values (default is to use the same
values as "SET.spp")
SET.labs <- SET.spp

# Define upper developmental threshold temperatures (set to "Inf" if unknown).
Values are specified for two species in this example.
UDT <- c(Inf, Inf, Inf, Inf)

# Define lower and upper lethal temperatures (set to "-Inf" and "Inf"
respectively is unknown or ignored).
LLT <- c(-Inf, -Inf, -Inf, -Inf)
ULT <- c(Inf, Inf, Inf, Inf)

# Define whether interpolated or unmanipulated time series should be used for
calculations.
DevTimes.Use.TTS <- "n"                                # use unmanipulated time
series data for calculations
DevTimes.Use.LongestDuration <- "n"
DevTimes.Use.Interpolated.TTS <- "y"
DevTimes.Use.Interpolated.Sinus.TTS <- "n"
DevTimes.Use.Interpolated.LongestDuration <- "n"

# Should generation times be calculated?
Do.GenTimes.Output <- "y"

# Should the number of potential generations per year calculated over each year?
Do.calculate.generations.each.year <- "y"

# If calculating the number of generations possible per year, specify the start
and end data (format: dd/mm) of the the year period (values here may specify a
season shorter than a full year to represent shorter activity / growing seasons
in seasonal environments).
Generations.StartDate <- "1/1"
Generations.EndDate <- "31/12"

# Should the number of potential generations be calculated over just one year
from the data (i.e. calculations will only be limited to a single period, and
not for each year in the time series)? This is a useful option if users want to
```

```
compare different time series that only partially overlap. Importantly, ensure
that the time period specified falls completely within the time series being
analysed (if not, the code will generate an error and the analysis will be
aborted).
Do.calculate.generations.one.year <- "n"

# Specify the start and end date (format: dd/mm/yyyy hh:mm:ss) if the number of
potential generations is to calculated for only one year from the data
Generations.StartDate4 <- "1/1/2013 00:00:00"
Generations.EndDate4 <- "31/12/2013 23:59:59"

# Should development times (the full results and the output summary) be
outputted, and should the results be plotted?
Do.DevTimes.Output <- "y"
Do.DevTimes.summary <- "y"
Do.DevTimes.Plot <- "y"

# Should the potential number of generations per year be plotted?
Plot.MaxGenerations <- "y"


### Beyond this point no further inputs are required from the user. Comments are
provided to broadly describe the analysis steps being implimented. ###


##########################
# 3. Open and check data #
##########################

# Check that the number of columns and sites (i.e. names of the variables to be
analysed) match up. If any discrepancy, then TTS will be set to Null (i.e.
aborting analysis) and a warning message will be displayed
if (length(Site) != length(TimeSeriesColumn) | length(Site) != length(Lat) |
length(Site) != length(Long) | length(Site) != length(TimeZone)) {cat("The
number of sites, columns, co-ordinates and/or time zones do not match\n")}

# Open the data file(s) using the read.delim() or read.csv() functions
if (FileFormat == "txt") {
  winDialog("ok", "Please choose the input data file (.txt)")
        TTS.all <- read.delim(file.choose(), sep = "\t", dec = ".", na.strings =
"", strip.white = TRUE, header = TRUE)
        if (NumberFiles > 1) {for (h in 2 : NumberFiles) {
    winDialog("ok", "Please choose additional input data file (.txt)")
    Temp <- read.table(file.choose(), sep = "\t", na.strings = "", dec = ".",
strip.white = TRUE, header = TRUE); TTS.all <- rbind(TTS.all, Temp)}}
} else {
  winDialog("ok", "Please choose the input data file (.csv)")
        TTS.all <- read.csv(file.choose(), sep = ",", dec = ".")
        if (NumberFiles > 1) {for (h in 2 : NumberFiles) {
    winDialog("ok", "Please choose additional input data file (.csv)")
    Temp <- read.csv(file.choose(), sep = ",", dec = ".", na.strings = "",
strip.white = TRUE, header = TRUE); TTS.all <- rbind(TTS.all, Temp)}}}
```

```
attach(TTS.all)
remove(NumberFiles)

# Format the date-time column(s), depending on if teh date-time data is
presented in 1 or 6 columns.
if (DateTime.in.six.columns == "y") {
        # create DateTime column and add to dataset
        DateTime <- as.POSIXlt(ISOdate(TTS.all$Year, TTS.all$Month, TTS.all$Day,
TTS.all$Hour, TTS.all$Minute, TTS.all$Second))
        TTS.all <- cbind(TTS.all, DateTime)
} else {
  TimeSeriesColumn <- TimeSeriesColumn + 5
        # TTS.all[, 1] = as.numeric(TTS.all[, 1])
        Year.temp <- (strptime(TTS.all[, 1], DateTime.format)$year) + 1900
        Month.temp <- (strptime(TTS.all[, 1], DateTime.format)$mon) + 1
        Day.temp <- strptime(TTS.all[, 1], DateTime.format)$mday
        Hour.temp <- strptime(TTS.all[, 1], DateTime.format)$hour
        Minute.temp <- strptime(TTS.all[, 1], DateTime.format)$min
        Second.temp <- strptime(TTS.all[, 1], DateTime.format)$sec
        TTS.all <- data.frame("Year" = Year.temp, "Month" = Month.temp, "Day" =
Day.temp, "Hour" = Hour.temp, "Minute" = Minute.temp, "Second" = Second.temp,
"Temperature" = TTS.all[, -(1)], "DateTime" = ISOdate(Year.temp, Month.temp,
Day.temp, Hour.temp, Minute.temp, Second.temp))
        DateTime <- as.POSIXlt(TTS.all$DateTime)
        remove(Year.temp, Month.temp, Day.temp, Hour.temp, Minute.temp,
Second.temp)}

# Check if year, month, day and hour values are within acceptable limits (e.g.
hours between 0 and 23)
CurrentYear <- as.POSIXlt(Sys.Date())$year + 1900
if (any(TTS.all$Year > CurrentYear)) {TTS <- NULL; cat("Incorrect year
coding\n")}
if (any(TTS.all$Month > 12 | TTS.all$Month < 1)) {TTS <- NULL; cat("Incorrect
month coding\n")}
if (any(TTS.all$Day > 31 | TTS.all$Day < 1)) {TTS <- NULL; cat("Incorrect month
coding\n")}
if (any(TTS.all$Hour > 24 | TTS.all$Hour < 0)) {TTS <- NULL; cat("Incorrect
month coding\n")}

# Check if DateTime values are in chronological order. If not, the code will
sort the dataset by DateTime
if (is.unsorted(TTS.all$DateTime, na.rm = FALSE, strictly = FALSE) == TRUE)
{TTS.all <- TTS.all[order(TTS.all$DateTime), ]}

# Check for DateTime duplicates, and remove all except the first records for any
duplicates
if (any(duplicated(TTS.all$DateTime)) == TRUE) {
        length.temp1 <- length(TTS.all$DateTime)
        TTS.all <- TTS.all[-which(duplicated(TTS.all$DateTime)), ]
        length.temp2 <- length(TTS.all$DateTime)
        print(paste(length.temp1 - length.temp2,"Duplicate records have been
```

```
deleted!"))
        remove(length.temp1, length.temp2)}

# Since "Temperature" used as placeholder name, none of the data columns from 
the original file can have this heading. Here the code checks if any original 
columns labeled "Temperature" and changes it to "Tmprtr"
if (any(names(TTS.all) == "Temperature")) {names(TTS.all)[which(names(TTS.all) 
== "Temperature")] <- "Tmprtr"}

# Output the list of site names and corresponding site numbers as a .csv format 
file.
if (is.null(SiteNames) == FALSE) {write.table(noquote(cbind(SiteNumber = Site, 
SiteName = SiteNames)), file = paste(OutputFileName, "_SiteNames.csv", sep = 
""), append = FALSE, sep = ",", dec = ".", quote = FALSE, na = "NA", col.names = 
TRUE, row.names = FALSE)}


################################################################################
#
# 4. Start analysing data, looping through each of the variables to be analysed
#
################################################################################
#

# Determine the number of variables to be analysed, and the total number of 
variable in the data file (including those not for analysis).
NumberTTS <- length(TimeSeriesColumn)
TotalTTS <- length(TTS.all) - 7

# Start looping through each variable to be analysed
for (ColNumber in 1 : NumberTTS) {


# Create subset of the data for analysis, comprising only the variable of 
interest
TTS.all$Temperature <- TTS.all[, TimeSeriesColumn[ColNumber]]

# Removes any rows in the data subset that are missing values
TTS <- subset(TTS.all, complete.cases(TTS.all$Temperature))
TTS <- TTS[, -(7 : (6 + TotalTTS))]

# Create empty columns to hold the variables that will be calculated next
TimeDiff <- vector(length = length(TTS$Temperature))
TimeChange <- vector(length = length(TTS$Temperature))
RateOfChange <- vector(length = length(TTS$Temperature))

# Calculate the time difference between the first record and all other records, 
and the rate of change and the time difference between all consecutive records. 
The rate of change at time "i" is calculated from "i - 1" to "i".
for (i in 2 : length(TimeDiff)) {
        TimeDiff[i] <- as.numeric(difftime(TTS$DateTime[i], TTS$DateTime[1], 
units = "hours"))
```

```
        TimeChange[i] <- as.numeric(difftime(TTS$DateTime[i], TTS$DateTime[i - 
1], units = "hours"))
        RateOfChange[i] <- (TTS$Temperature[i] - TTS$Temperature[i - 1]) / 
as.numeric(difftime(TTS$DateTime[i], TTS$DateTime[i - 1], units = "hours"))}

# Because the first recording has no previous recording from which to calculate 
the previous time and temperature, it is assigned a value of zero rate of 
change.
RateOfChange[1] <- 0

# Add the three new columns to the dataset
TTS <- cbind(TTS,RateOfChange, TimeDiff, TimeChange)
remove(RateOfChange, TimeDiff, TimeChange)




################################################################################
# 5. Identify gaps in the time series and perform required interpolation      #
#                                                                              #
# 5a. Identify longest duration of time series without gaps in the time series #
################################################################################

Interpolated.TTS <- TTS        # for cases where no interpolation is conducted 
(or required)

# Find any sections of the time series where the time difference between 
consecutive recordings is greater than the threshold specified to identify a gap 
(i.e. missing data) in the data (i.e. where missing data covers a period great 
than that specified for ShortGap)
# The code then records where these gaps are located in the time series, and 
records the longest duration of the time series without any gaps

if (any(TTS$TimeChange > ShortGap)) {
        Gaps <- which(TTS$TimeChange > ShortGap)
        Gaps <- c(1, Gaps, length(TTS$TimeChange))
        non.gap.duration <- vector(length = length(Gaps) - 1)
        for (i in 1 : (length(Gaps) - 1)) {non.gap.duration[i] <- Gaps[i + 1] - 
Gaps[i]}
        LongestDuration.start <- Gaps[which(max(non.gap.duration) == 
non.gap.duration)]
        LongestDuration.end <- Gaps[which(max(non.gap.duration) == 
non.gap.duration) + 1] - 1
        LongestDuration <- TTS[LongestDuration.start : LongestDuration.end, ]
        LongestDuration$TimeChange[1] <- LongestDuration$TimeChange[2]
} else {LongestDuration <- TTS
        Gaps <- c(1, length(TTS$TimeChange))
        LongestDuration.start <- 1
        LongestDuration.end <- length(TTS$TimeChange)
        non.gap.duration <- length(TTS$TimeChange)}
```

```
#############################################################
# 5b. Linear interpolation to fill all gaps in time series #
#############################################################

if (Do.Interpolation == "y") {
# Gaps are identified at the start and end of the entire time series. Hence
interpolation is only used when the value of Gaps exceeds two (i.e. indicating
that there is a segment of missing values in the time series that exceeds the
limit defined as ShortGap).
if (length(Gaps) > 2) {
	GapLength <- vector(length = length(Gaps) - 2)
	for (i in 2 : (length(Gaps) -  1)) {
		Temp.Interpolated.DateTime <- seq(from =
as.POSIXlt(TTS$DateTime[Gaps[i] - 1]), to = as.POSIXlt(TTS$DateTime[Gaps[i]]),
by = as.numeric(60 * 60 * median(TTS$TimeChange)))
		Temp.Interpolated.DateTime <- Temp.Interpolated.DateTime[-1]
		if (TTS$DateTime[Gaps[i]] ==
Temp.Interpolated.DateTime[length(Temp.Interpolated.DateTime)])
{Temp.Interpolated.DateTime <-
Temp.Interpolated.DateTime[-length(Temp.Interpolated.DateTime)]}
		GapLength[i - 1] <- length(Temp.Interpolated.DateTime)
		if (non.gap.duration[i - 1] < RecordsForInterpolation |
non.gap.duration[i] < RecordsForInterpolation) {TempRecordsForInterpolation <-
min(non.gap.duration[i - 1], non.gap.duration[i]); print("Not enough records to
interpolate across some missing data; period of data required for interpolation
reduced")} else {TempRecordsForInterpolation <- RecordsForInterpolation}
		Temp.Interpolated.TTS <- data.frame(
			as.numeric(format(Temp.Interpolated.DateTime,'%Y')),
			as.numeric(format(Temp.Interpolated.DateTime,'%m')),
			as.numeric(format(Temp.Interpolated.DateTime,'%d')),
			as.numeric(format(Temp.Interpolated.DateTime,'%H')),
			as.numeric(format(Temp.Interpolated.DateTime,'%M')),
			as.numeric(format(Temp.Interpolated.DateTime,'%S')),
			Temp.Interpolated.DateTime,
			seq(from = mean(TTS$Temperature[Gaps[i] - 1 - (0 :
(TempRecordsForInterpolation - 1))]), to = mean(TTS$Temperature[Gaps[i] + 1 + (0
: (TempRecordsForInterpolation - 1))]), length.out = GapLength[i - 1]))
		remove(Temp.Interpolated.DateTime)
		if (i == 2) {Interpolated.TTS <- Temp.Interpolated.TTS} else
{Interpolated.TTS <- rbind(Interpolated.TTS, Temp.Interpolated.TTS)}}
	names(Interpolated.TTS) <- names(TTS[, 1 : 8])
	Interpolated.TTS <- rbind(TTS[, 1 : 8], Interpolated.TTS)
	Interpolated.TTS <- Interpolated.TTS[order(Interpolated.TTS$DateTime), ]
	Interpolated.TimeDiff <- vector(length =
length(Interpolated.TTS$DateTime))
	Interpolated.TimeChange <- vector(length =
length(Interpolated.TTS$DateTime))
	Interpolated.RateOfChange <- vector(length =
length(Interpolated.TTS$DateTime))
	for (i in 2 : length(Interpolated.TTS$DateTime)) {
		Interpolated.TimeDiff[i] <-
as.numeric(difftime(Interpolated.TTS$DateTime[i], Interpolated.TTS$DateTime[1],
```

```
units = "hours"))
                Interpolated.TimeChange[i] <-
as.numeric(difftime(Interpolated.TTS$DateTime[i], Interpolated.TTS$DateTime[i -
1], units = "hours"))
                Interpolated.RateOfChange[i] <- (Interpolated.TTS$Temperature[i]
- Interpolated.TTS$Temperature[i - 1]) /
as.numeric(difftime(Interpolated.TTS$DateTime[i], Interpolated.TTS$DateTime[i -
1], units = "hours"))}
        Interpolated.TTS <- cbind(Interpolated.TTS, Interpolated.RateOfChange,
Interpolated.TimeDiff, Interpolated.TimeChange)
        remove(Interpolated.RateOfChange, Interpolated.TimeDiff,
Interpolated.TimeChange, Temp.Interpolated.TTS)
        names(Interpolated.TTS) <- names(LongestDuration)
} else {Interpolated.TTS <- LongestDuration}           # if there are no gaps,
then Interpolated TTS == Longest Duration



################################################################
# 5c. Sinusoidal interpolation to fill all gaps in time series #
################################################################

if (Do.Sinusoidal.Interpolation == "y") {
if (length(Gaps) > 2) {
        for (i in 2 : (length(Gaps) - 1)) {
                Temp.Interpolated.DateTime <- seq(from = TTS$DateTime[Gaps[i] -
1], to = TTS$DateTime[Gaps[i]], by = as.numeric(60 * 60 *
median(TTS$TimeChange)))
                Temp.Interpolated.DateTime <- Temp.Interpolated.DateTime[-1]
                if (TTS$DateTime[Gaps[i]] ==
Temp.Interpolated.DateTime[length(Temp.Interpolated.DateTime)])
{Temp.Interpolated.DateTime <- Temp.Interpolated.DateTime[ -
length(Temp.Interpolated.DateTime)]}
                GapLength[i - 1] <- length(Temp.Interpolated.DateTime)
                if (non.gap.duration[i - 1] < RecordsForInterpolation)
{TempRecordsForInterpolation <- non.gap.duration[i - 1]; print("Not enough
records to interpolate across some missing data with full number of records;
period of data required for interpolation reduced")} else
{TempRecordsForInterpolation <- RecordsForInterpolation}
                Temp.Interpolated.TTS<-data.frame(
                        as.numeric(format(Temp.Interpolated.DateTime,'%Y')),
                        as.numeric(format(Temp.Interpolated.DateTime,'%m')),
                        as.numeric(format(Temp.Interpolated.DateTime,'%d')),
                        as.numeric(format(Temp.Interpolated.DateTime,'%H')),
                        as.numeric(format(Temp.Interpolated.DateTime,'%M')),
                        as.numeric(format(Temp.Interpolated.DateTime,'%S')),
                        Temp.Interpolated.DateTime)
                Temp.Interpolated.TTS <-
Temp.Interpolated.TTS[order(Temp.Interpolated.TTS[, 7]), ]
                Temp.Interpolated.TimeDiff <- vector(length =
length(Temp.Interpolated.TTS[, 7]))
                Temp.Interpolated.TimeChange <- vector(length =
```

```
length(Temp.Interpolated.TTS[, 7]))
                Temp.Interpolated.RateOfChange <- vector(length =
length(Temp.Interpolated.TTS[, 7]))
                for (j in 2 : length(Temp.Interpolated.TTS[, 7])) {
                        Temp.Interpolated.TimeDiff[j] <-
as.numeric(difftime(Temp.Interpolated.TTS[, 7][j], Temp.Interpolated.TTS[,
7][1], units = "hours"))
                        Temp.Interpolated.TimeChange[j] <-
as.numeric(difftime(Temp.Interpolated.TTS[, 7][j], Temp.Interpolated.TTS[, 7][j
- 1], units = "hours"))}

                sinus.start.mean <- mean(TTS$Temperature[Gaps[i] - 1 - (0 :
(TempRecordsForInterpolation - 1))])
                sinus.end.mean <- mean(TTS$Temperature[Gaps[i] + 1 + (0 :
(TempRecordsForInterpolation - 1))])
                sinus.linear.coeff <- (sinus.end.mean - sinus.start.mean) /
(GapLength[i - 1] - 2)
                sinus.trend.start <- lm(TTS$Temperature[Gaps[i] - 1 - (0 :
(TempRecordsForInterpolation - 1))] ~ TTS$TimeDiff[Gaps[i] - 1 - (0 :
(TempRecordsForInterpolation - 1))] + sin(2 * pi * TTS$TimeDiff[Gaps[i] - 1 - (0
: (TempRecordsForInterpolation - 1))] / (24 * median(TTS$TimeChange))) + cos(2 *
pi * TTS$TimeDiff[Gaps[i] - 1 - (0 : (TempRecordsForInterpolation - 1))] / (24 *
median(TTS$TimeChange))))
                sinus.trend.end <- lm(TTS$Temperature[Gaps[i] + 1 + (0 :
(TempRecordsForInterpolation - 1))] ~ TTS$TimeDiff[Gaps[i] + 1 + (0 :
(TempRecordsForInterpolation - 1))] + sin(2 * pi * TTS$TimeDiff[Gaps[i] + 1 + (0
: (TempRecordsForInterpolation - 1))] / (24 * median(TTS$TimeChange))) + cos(2 *
pi * TTS$TimeDiff[Gaps[i] + 1 + (0 : (TempRecordsForInterpolation - 1))] / (24 *
median(TTS$TimeChange))))
                sinus.start.sine.amplitude <- sinus.trend.start$coeff[3]
                sinus.end.sine.amplitude <- sinus.trend.end$coeff[3]
                sinus.trend.sine.amplitude <- (sinus.end.sine.amplitude -
sinus.start.sine.amplitude) / (GapLength[i - 1] - 2)
                sinus.start.cosine.amplitude <- sinus.trend.start$coeff[4]
                sinus.end.cosine.amplitude <- sinus.trend.end$coeff[4]
                sinus.trend.cosine.amplitude <- (sinus.end.cosine.amplitude -
sinus.start.cosine.amplitude) / (GapLength[i - 1] - 2)

                Temp.Interpolated.Sinus.Temperatures <- sinus.start.mean +
(sinus.linear.coeff * Temp.Interpolated.TimeDiff) + ((sinus.start.sine.amplitude
+ sinus.trend.sine.amplitude * Temp.Interpolated.TimeDiff) * sin(2 * pi *
(Temp.Interpolated.TTS[, 4] + 1) /(24 * median(TTS$TimeChange)))) +
((sinus.start.cosine.amplitude + sinus.trend.cosine.amplitude *
Temp.Interpolated.TimeDiff) * cos(2 * pi * (Temp.Interpolated.TTS[, 4] + 1) /
(24 * median(TTS$TimeChange))))

                for (j in 2 : length(Temp.Interpolated.TTS[, 7]))
                        {Temp.Interpolated.RateOfChange[j] <-
(Temp.Interpolated.Sinus.Temperatures[j] -
Temp.Interpolated.Sinus.Temperatures[j - 1]) /
as.numeric(difftime(Temp.Interpolated.TTS[, 7][j], Temp.Interpolated.TTS[, 7][j
- 1], units = "hours"))}
```

```
                Temp.Interpolated.Sinus.TTS <- cbind(Temp.Interpolated.TTS, 
Temp.Interpolated.Sinus.Temperatures, Temp.Interpolated.RateOfChange, 
Temp.Interpolated.TimeDiff, Temp.Interpolated.TimeChange)
                names(Temp.Interpolated.Sinus.TTS) <- names(LongestDuration)
                if (i == 2) {Interpolated.Sinus.TTS <- rbind(TTS, 
Temp.Interpolated.Sinus.TTS)} else {Interpolated.Sinus.TTS <- 
rbind(Interpolated.Sinus.TTS, Temp.Interpolated.Sinus.TTS)}
        remove(Temp.Interpolated.RateOfChange, Temp.Interpolated.TimeDiff, 
Temp.Interpolated.TimeChange, Temp.Interpolated.Sinus.TTS, 
Temp.Interpolated.TTS)
        remove(Temp.Interpolated.DateTime)}
Interpolated.Sinus.TTS <- 
Interpolated.Sinus.TTS[order(Interpolated.Sinus.TTS$DateTime), ]
for (j in 2 : length(Interpolated.Sinus.TTS[, 7])) {
        Interpolated.Sinus.TTS$TimeDiff[j] <- 
as.numeric(difftime(Interpolated.Sinus.TTS[, 7][j], Interpolated.Sinus.TTS[, 
7][1], units = "hours"))
        Interpolated.Sinus.TTS$TimeChange[j] <- 
as.numeric(difftime(Interpolated.Sinus.TTS[, 7][j], Interpolated.Sinus.TTS[, 
7][j - 1], units = "hours"))
        Interpolated.Sinus.TTS$RateOfChange[j] <- 
(Interpolated.Sinus.TTS$Temperature[j] - Interpolated.Sinus.TTS$Temperature[j - 
1]) / as.numeric(difftime(Interpolated.Sinus.TTS[, 7][j], 
Interpolated.Sinus.TTS[, 7][j - 1], units = "hours"))}
} else {Interpolated.Sinus.TTS <- LongestDuration}}  # if there are no gaps, 
then Interpolated TTS == Longest Duration



###################################################################
# 5d. Linear interpolation to fill only short gaps in time series #
###################################################################

if (Do.Interpolation.Across.Short.Gaps == "y") {

if (any(TTS$TimeChange > LongGap)) {
        LongGaps <- which(TTS$TimeChange > LongGap)
        LongGaps <- c(1, LongGaps,length(TTS$TimeChange))
        LongGaps.non.gap.duration <- vector(length = length(LongGaps) - 1)
        for (i in 1 : (length(LongGaps) - 1)) {LongGaps.non.gap.duration[i] <- 
LongGaps[i + 1] - LongGaps[i]}
        LongGaps.LongestDuration.start <- 
LongGaps[which(max(LongGaps.non.gap.duration) == LongGaps.non.gap.duration)]
        LongGaps.LongestDuration.end <- 
LongGaps[which(max(LongGaps.non.gap.duration) == LongGaps.non.gap.duration) + 1] 
- 1
        LongGaps.LongestDuration <- TTS[LongGaps.LongestDuration.start : 
LongGaps.LongestDuration.end, ]
        LongGaps.LongestDuration$TimeChange[1] <- 
LongGaps.LongestDuration$TimeChange[2]

if (any(LongGaps.LongestDuration$TimeChange > ShortGap)) {
```

```
        ShortGaps <- which(LongGaps.LongestDuration$TimeChange > ShortGap)
        ShortGaps <- c(1, ShortGaps,length(LongGaps.LongestDuration$TimeChange))
        ShortGaps.non.gap.duration <- vector(length = length(ShortGaps) - 1)
        for (i in 1 : (length(ShortGaps) - 1)) {ShortGaps.non.gap.duration[i] <-
ShortGaps[i + 1] - ShortGaps[i]}

        ShortGapLength <- vector(length = length(ShortGaps) - 2)
        for (i in 2 : (length(ShortGaps) - 1)) {
                Temp.Interpolated.LongestDuration.DateTime <- seq(from =
LongGaps.LongestDuration$DateTime[ShortGaps[i] - 1], to =
LongGaps.LongestDuration$DateTime[ShortGaps[i]], by = as.numeric(60 * 60 *
median(LongGaps.LongestDuration$TimeChange)))
                ShortGapLength[i - 1] <-
length(Temp.Interpolated.LongestDuration.DateTime)
                if (ShortGaps.non.gap.duration[i - 1] < RecordsForInterpolation)
{TempRecordsForInterpolation <- ShortGaps.non.gap.duration[i-1]; print("Not
enough records to interpolate across some missing data with full number of
records; period of data required for interpolation reduced")} else
{TempRecordsForInterpolation <- RecordsForInterpolation}
                Temp.Interpolated.LongestDuration.TTS <- data.frame(

as.numeric(format(Temp.Interpolated.LongestDuration.DateTime,'%Y')),

as.numeric(format(Temp.Interpolated.LongestDuration.DateTime,'%m')),

as.numeric(format(Temp.Interpolated.LongestDuration.DateTime,'%d')),

as.numeric(format(Temp.Interpolated.LongestDuration.DateTime,'%H')),

as.numeric(format(Temp.Interpolated.LongestDuration.DateTime,'%M')),

as.numeric(format(Temp.Interpolated.LongestDuration.DateTime,'%S')),
                        Temp.Interpolated.LongestDuration.DateTime,
                        seq(from =
mean(LongGaps.LongestDuration$Temperature[ShortGaps[i] - 1 - (0 :
(TempRecordsForInterpolation - 1))], na.rm = TRUE), to =
mean(LongGaps.LongestDuration$Temperature[ShortGaps[i] + 1 + (0 :
(TempRecordsForInterpolation - 1))], na.rm = TRUE), length.out =
ShortGapLength[i - 1]))
                if (i == 2) {Interpolated.LongestDuration.TTS <-
Temp.Interpolated.LongestDuration.TTS} else {Interpolated.LongestDuration.TTS <-
rbind(Interpolated.LongestDuration.TTS, Temp.Interpolated.LongestDuration.TTS)}}
                remove(Temp.Interpolated.LongestDuration.DateTime,
Temp.Interpolated.LongestDuration.TTS)
        names(Interpolated.LongestDuration.TTS) <-
names(LongGaps.LongestDuration[, 1 : 8])
        Interpolated.LongestDuration.TTS <- rbind(LongGaps.LongestDuration[, 1 :
8], Interpolated.LongestDuration.TTS)
        Interpolated.LongestDuration.TTS <-
Interpolated.LongestDuration.TTS[order(Interpolated.LongestDuration.TTS$DateTime
), ]
        Interpolated.LongestDuration.TimeDiff <- vector(length =
```

```
length(Interpolated.LongestDuration.TTS$DateTime))
        Interpolated.LongestDuration.TimeChange <- vector(length =
length(Interpolated.LongestDuration.TTS$DateTime))
        Interpolated.LongestDuration.RateOfChange <- vector(length =
length(Interpolated.LongestDuration.TTS$DateTime))
        for (i in 2 : length(Interpolated.LongestDuration.TTS$DateTime)) {
                Interpolated.LongestDuration.TimeDiff[i] <-
as.numeric(difftime(Interpolated.LongestDuration.TTS$DateTime[i],
Interpolated.LongestDuration.TTS$DateTime[1], units = "hours"))
                Interpolated.LongestDuration.TimeChange[i] <-
as.numeric(difftime(Interpolated.LongestDuration.TTS$DateTime[i],
Interpolated.LongestDuration.TTS$DateTime[i - 1], units = "hours"))
                Interpolated.LongestDuration.RateOfChange[i] <-
(Interpolated.LongestDuration.TTS$Temperature[i] -
Interpolated.LongestDuration.TTS$Temperature[i - 1]) /
as.numeric(difftime(Interpolated.LongestDuration.TTS$DateTime[i],
Interpolated.LongestDuration.TTS$DateTime[i - 1], units = "hours"))}
        Interpolated.LongestDuration.TTS <-
cbind(Interpolated.LongestDuration.TTS,
Interpolated.LongestDuration.RateOfChange,
Interpolated.LongestDuration.TimeDiff, Interpolated.LongestDuration.TimeChange)
        remove(Interpolated.LongestDuration.RateOfChange,
Interpolated.LongestDuration.TimeDiff, Interpolated.LongestDuration.TimeChange)
        names(Interpolated.LongestDuration.TTS) <- names(LongestDuration)
} else {
        Interpolated.LongestDuration.TTS <- LongestDuration; ShortGaps <- c(1,
length(LongGaps.LongestDuration$TimeChange))}   # This occurs if there are no
short gaps, but there are nonetheless long gaps in the time series.

} else {
        Interpolated.LongestDuration.TTS <- Interpolated.TTS; ShortGaps <- Gaps;
LongGaps <- Gaps; LongGaps.LongestDuration.start <- 1}  # This occurs if there
are no long gaps in the time series (although there maybe still be short gaps in
the time series).

}



###################################################
# 6. Loop through each segment of the time series #
###################################################

# If the user has chosen to analyse segments of the time series that are
separated by a LongGap individually (by specifying that Do.Analyses.Each.Segment
= "y"), then here each segment is considered in turn. Segments will named
according to the variable number, followed by the segment number. Thus "1.1"
will refer to the first segment of the first variable; "3.8" will refer to the
eighth segment of the third variable.

TTS.no.segments <- TTS
```

```
if (Do.Analyses.Each.Segment == "y") {
        if (Do.Analyses.Each.Segment.No.Interpolation == "y") {
                NumberSegments = length(Gaps) - 1
                SegGaps <- Gaps}
        if (Do.Analyses.Each.Segment.With.Interpolation == "y") {
                NumberSegments = length(LongGaps) - 1
                SegGaps <- LongGaps}
        } else {NumberSegments = 1; SegGaps <- c(1, length(TTS$DateTime))}

for (SegNumber in 1 : NumberSegments) {
        if (SegNumber != NumberSegments) {TTS <-
TTS.no.segments[SegGaps[SegNumber] : SegGaps[SegNumber + 1] - 1, ]} else {TTS <-
TTS.no.segments[SegGaps[SegNumber] : SegGaps[SegNumber + 1], ]}




#####################################################
# 7. Calculate annual, monthly and daily summaries #
#####################################################

# Create output matrices & name the parameters being calculated.
ParameterNames <- c("n", "Mean", "Maximum", "Minimum", "Range", "SD", "SE",
"C.V.", "MaxRoC", "MinRoC", "MeanDTR", "MaxDTR", "MinDTR", "MeanMTR", "MaxMTR",
"MinMTR", "Isotherm.", paste(Quantile * 100,"% Qu. Temp"), paste((1 - Quantile)
* 100,"% Qu. Temp"), paste(Quantile * 100,"% RoC"), paste((1 - Quantile) *
100,"% RoC"), paste(Quantile * 100,"% DTR"), paste((1 - Quantile) * 100,"%
DTR"), paste(Quantile * 100,"% MTR"), paste((1 - Quantile) * 100,"% MTR"))
DatasetOutput <- matrix(ncol = length(ParameterNames) + 1, nrow = 1)
YearlyOutput <- matrix(ncol = length(ParameterNames) + 2, nrow = 1 +
max(TTS$Year) - min(TTS$Year))
MonthlyOutput <- matrix(ncol = length(ParameterNames) + 3, nrow = (1 +
max(TTS$Year) - min(TTS$Year)) * 12)
DailyOutput <- matrix(ncol = length(ParameterNames) + 4, nrow =
length(unique(paste(TTS$Year, TTS$Month, TTS$Day))))
MonthlySummary <- matrix(ncol = length(ParameterNames) + 2, nrow = 12)

colnames(DatasetOutput) <- c("Site", ParameterNames)
colnames(YearlyOutput) <- c("Site", "Year", ParameterNames)
colnames(MonthlyOutput) <- c("Site", "Year", "Month", ParameterNames)
colnames(DailyOutput) <- c("Site", "Year", "Month", "Day", ParameterNames)
colnames(MonthlySummary) <- c("Site", "Month", ParameterNames)

# Calculate statistics per year, month and day. The code loops through the date
data, and defines a day as a period for which the year, month and day values do
not change. Similarly, a month is defined as a period during which the year and
month values do not change, etc.
# "i" counts the number of rows in the dataset through which the code has
already looped; "j" counts the number of recordings within each day; "k" counts
how many rows have already been filled in the output file.
# Because the last day's end cannot be defined by a change in year, month or day
values, the code is repeated for the last day.
```

```
i = 1
j = 0
k = 1

while (i + j <= length(TTS$Year)) {
        if ((TTS$Day[i] == TTS$Day[i + j]) & (TTS$Month[i] == TTS$Month[i + j])
& (TTS$Year[i] == TTS$Year[i + j])) {
                j = j + 1
        } else {DailyOutput[k, 1] = as.numeric(paste(Site[ColNumber], ".",
SegNumber, sep = ""))
                DailyOutput[k, 2] = TTS$Year[i]
                DailyOutput[k, 3] = TTS$Month[i]
                DailyOutput[k, 4] = TTS$Day[i]
                DailyOutput[k, 5] = length(TTS$Temperature[TTS$Year ==
TTS$Year[i] & TTS$Month == TTS$Month[i] & TTS$Day == TTS$Day[i]])
                DailyOutput[k, 6] = mean(TTS$Temperature[TTS$Year == TTS$Year[i]
& TTS$Month == TTS$Month[i] & TTS$Day == TTS$Day[i]])
                DailyOutput[k, 7] = max(TTS$Temperature[TTS$Year == TTS$Year[i]
& TTS$Month == TTS$Month[i] & TTS$Day == TTS$Day[i]])
                DailyOutput[k, 8] = min(TTS$Temperature[TTS$Year == TTS$Year[i]
& TTS$Month == TTS$Month[i] & TTS$Day == TTS$Day[i]])
                if (DailyOutput[k, 5] < MinDailyRecords) {
                        DailyOutput[k, 9] = NA
                } else {
                        DailyOutput[k, 9] = DailyOutput[k, 7] - DailyOutput[k,
8]}
                DailyOutput[k, 10] = sd(TTS$Temperature[TTS$Year == TTS$Year[i]
& TTS$Month == TTS$Month[i] & TTS$Day == TTS$Day[i]])
                DailyOutput[k, 11] = DailyOutput[k, 10] / sqrt(DailyOutput[k,
5])
                DailyOutput[k, 12] = DailyOutput[k, 10] / DailyOutput[k, 6]
                if (DailyOutput[k, 5] < MinDailyRecords) {
                        DailyOutput[k, 13] = NA
                        DailyOutput[k, 14] = NA
                } else {
                        DailyOutput[k, 13] = max(TTS$RateOfChange[TTS$Year ==
TTS$Year[i] & TTS$Month == TTS$Month[i] & TTS$Day == TTS$Day[i]])
                        DailyOutput[k,14] = min(TTS$RateOfChange[TTS$Year ==
TTS$Year[i] & TTS$Month == TTS$Month[i] & TTS$Day == TTS$Day[i]])}

                # Columns 15 - 21 are reserved for DTR (daily temperature
range), MTR (monthly temperature range)and isothermality values (redundant or
not applicable for daily data summaries).
                i = i + j
                j = 0
                k = k + 1}}

        # Repeat the calculations for the last day in the time series
        DailyOutput[k, 1] = as.numeric(paste(Site[ColNumber], ".", SegNumber,
sep = ""))
        DailyOutput[k, 2] = TTS$Year[i]
```

```
        DailyOutput[k, 3] = TTS$Month[i]
        DailyOutput[k, 4] = TTS$Day[i]
        DailyOutput[k, 5] = length(TTS$Temperature[TTS$Year == TTS$Year[i] &
TTS$Month == TTS$Month[i] & TTS$Day == TTS$Day[i]])
        DailyOutput[k, 6] = mean(TTS$Temperature[TTS$Year == TTS$Year[i] &
TTS$Month == TTS$Month[i] & TTS$Day == TTS$Day[i]])
        DailyOutput[k, 7] = max(TTS$Temperature[TTS$Year == TTS$Year[i] &
TTS$Month == TTS$Month[i] & TTS$Day == TTS$Day[i]])
        DailyOutput[k, 8] = min(TTS$Temperature[TTS$Year == TTS$Year[i] &
TTS$Month == TTS$Month[i] & TTS$Day == TTS$Day[i]])
        if (DailyOutput[k, 5] < MinDailyRecords) {
                DailyOutput[k, 9] = NA
        } else {
                DailyOutput[k, 9] = DailyOutput[k, 7] - DailyOutput[k, 8]}
        DailyOutput[k, 10] = sd(TTS$Temperature[TTS$Year == TTS$Year[i] &
TTS$Month == TTS$Month[i] & TTS$Day == TTS$Day[i]])
        DailyOutput[k, 11] = DailyOutput[k, 10] / sqrt(DailyOutput[k, 5])
        DailyOutput[k, 12] = DailyOutput[k, 10] / DailyOutput[k, 6]
        if (DailyOutput[k, 5]<MinDailyRecords) {
                DailyOutput[k, 13] = NA
                DailyOutput[k, 14] = NA
        } else {
                DailyOutput[k, 13] = max(TTS$RateOfChange[TTS$Year ==
TTS$Year[i] & TTS$Month == TTS$Month[i] & TTS$Day == TTS$Day[i]])
                DailyOutput[k, 14] = min(TTS$RateOfChange[TTS$Year ==
TTS$Year[i] & TTS$Month == TTS$Month[i] & TTS$Day == TTS$Day[i]])}


# Calculate statistics per year and month
for (i in min(TTS$Year) : max(TTS$Year)) {
        l = i - min(TTS$Year)
        for (k in 1 : 12) {
                MonthlyOutput[k +(l * 12), 1] =
as.numeric(paste(Site[ColNumber], ".", SegNumber, sep = ""))
                MonthlyOutput[k +(l * 12), 2] = i
                MonthlyOutput[k +(l * 12), 3] = k
                MonthlyOutput[k +(l * 12), 4] = length(TTS$Temperature[TTS$Year
== i & TTS$Month == k])
                if (MonthlyOutput[k +(l * 12), 4] > 0) {
                MonthlyOutput[k + (l * 12), 5] = mean(TTS$Temperature[TTS$Year
== i & TTS$Month == k], na.rm = TRUE)
                MonthlyOutput[k + (l * 12), 6] = max(TTS$Temperature[TTS$Year ==
i & TTS$Month == k], na.rm = TRUE)
                MonthlyOutput[k + (l * 12), 7] = min(TTS$Temperature[TTS$Year ==
i & TTS$Month == k], na.rm = TRUE)
                MonthlyOutput[k + (l * 12), 8] = MonthlyOutput[k + (l * 12),6] -
MonthlyOutput[k + (l * 12), 7]
                MonthlyOutput[k + (l * 12), 9] = sd(TTS$Temperature[TTS$Year ==
i & TTS$Month == k])
                MonthlyOutput[k + (l * 12), 10] = MonthlyOutput[k + (l * 12), 9]
/ sqrt(MonthlyOutput[k + (l * 12), 4])
                MonthlyOutput[k + (l * 12), 11] = MonthlyOutput[k + (l * 12), 9]
```

```
/ MonthlyOutput[k + (l * 12), 5]
                MonthlyOutput[k + (l * 12), 12] = max(TTS$RateOfChange[TTS$Year
== i & TTS$Month == k], na.rm = TRUE)
                MonthlyOutput[k + (l * 12), 13] = min(TTS$RateOfChange[TTS$Year
== i & TTS$Month == k], na.rm = TRUE)
                MonthlyOutput[k + (l * 12), 14] = mean(DailyOutput[,
9][DailyOutput[, 2] == i & DailyOutput[, 3] == k], na.rm = TRUE)
                MonthlyOutput[k + (l * 12), 15] = max(DailyOutput[,
9][DailyOutput[, 2] == i & DailyOutput[, 3] == k], na.rm = TRUE)
                MonthlyOutput[k + (l * 12), 16] = min(DailyOutput[,
9][DailyOutput[, 2] == i & DailyOutput[, 3] == k], na.rm = TRUE)
                MonthlyOutput[k + (l * 12), 21] =
quantile(TTS$Temperature[TTS$Year == i & TTS$Month == k], Quantile, na.rm =
TRUE)
                MonthlyOutput[k + (l * 12), 22] =
quantile(TTS$Temperature[TTS$Year == i & TTS$Month == k], 1 - Quantile, na.rm =
TRUE)
                MonthlyOutput[k + (l * 12), 23] =
quantile(TTS$RateOfChange[TTS$Year == i & TTS$Month == k], Quantile, na.rm =
TRUE)
                MonthlyOutput[k + (l * 12), 24] =
quantile(TTS$RateOfChange[TTS$Year == i & TTS$Month == k], 1 - Quantile, na.rm =
TRUE)
                MonthlyOutput[k + (l * 12), 25] = quantile(DailyOutput[,
9][DailyOutput[, 2] == i & DailyOutput[, 3] == k], Quantile, na.rm = TRUE)
                MonthlyOutput[k + (l * 12), 26] = quantile(DailyOutput[,
9][DailyOutput[, 2] == i & DailyOutput[, 3] == k], 1 - Quantile, na.rm =
TRUE)}}}
MonthlyOutput <- MonthlyOutput[MonthlyOutput[, 4] > 0, ]
if (is.null(dim(MonthlyOutput))) {dim(MonthlyOutput) <- c(1,
length(ParameterNames) + 3)}

# Calculate the statistics per month (i.e. to determine seasonal patterns,
irrespective of year).
for (k in 1 : 12) {
        MonthlySummary[k, 1] = as.numeric(paste(Site[ColNumber], ".", SegNumber,
sep = ""))
        MonthlySummary[k, 2] = k
        MonthlySummary[k, 3] = length(TTS$Temperature[TTS$Month == k])
        MonthlySummary[k, 4] = mean(TTS$Temperature[TTS$Month == k], na.rm =
TRUE)
        MonthlySummary[k, 5] = max(TTS$Temperature[TTS$Month == k], na.rm =
TRUE)
        MonthlySummary[k, 6] = min(TTS$Temperature[TTS$Month == k], na.rm =
TRUE)
        MonthlySummary[k, 7] = MonthlySummary[k, 5] - MonthlySummary[k, 6]
        MonthlySummary[k, 8] = sd(TTS$Temperature[TTS$Month == k])
        MonthlySummary[k, 9] = MonthlySummary[k, 8] / sqrt(MonthlySummary[k, 3])
        MonthlySummary[k, 10] = MonthlySummary[k, 8] / MonthlySummary[k, 4]
        MonthlySummary[k, 11] = max(TTS$RateOfChange[TTS$Month == k], na.rm =
TRUE)
        MonthlySummary[k, 12] = min(TTS$RateOfChange[TTS$Month == k], na.rm =
```

```
TRUE)
        MonthlySummary[k, 13] = mean(DailyOutput[, 9][DailyOutput[, 3] == k],
na.rm = TRUE)
        MonthlySummary[k, 14] = max(DailyOutput[, 9][DailyOutput[, 3] == k],
na.rm = TRUE)
        MonthlySummary[k, 15] = min(DailyOutput[, 9][DailyOutput[, 3] == k],
na.rm = TRUE)
        MonthlySummary[k, 16] = mean(MonthlyOutput[, 8][MonthlyOutput[, 3] == k
& is.na(MonthlyOutput[, 5]) == FALSE])
        MonthlySummary[k, 17] = max(MonthlyOutput[, 8][MonthlyOutput[, 3] == k &
is.na(MonthlyOutput[, 5]) == FALSE])
        MonthlySummary[k, 18] = min(MonthlyOutput[, 8][MonthlyOutput[, 3] == k &
is.na(MonthlyOutput[, 5]) == FALSE])
        MonthlySummary[k, 20] = quantile(TTS$Temperature[TTS$Month == k], na.rm
= TRUE, Quantile)
        MonthlySummary[k, 21] = quantile(TTS$Temperature[TTS$Month == k], na.rm
= TRUE, 1 - Quantile)
        MonthlySummary[k, 22] = quantile(TTS$RateOfChange[TTS$Month == k],
Quantile, na.rm = TRUE)
        MonthlySummary[k, 23] = quantile(TTS$RateOfChange[TTS$Month == k], 1 -
Quantile, na.rm = TRUE)
        MonthlySummary[k, 24] = quantile(DailyOutput[, 9][DailyOutput[,3] == k],
Quantile, na.rm = TRUE)
        MonthlySummary[k, 25] = quantile(DailyOutput[, 9][DailyOutput[,3] == k],
1 - Quantile, na.rm = TRUE)
        MonthlySummary[k, 26] = quantile(MonthlyOutput[, 8][MonthlyOutput[,3] ==
k], Quantile, na.rm = TRUE)
        MonthlySummary[k, 27] = quantile(MonthlyOutput[, 8][MonthlyOutput[,3] ==
k], 1 - Quantile, na.rm = TRUE)}
MonthlySummary <- MonthlySummary[MonthlySummary[, 3] > 0, ]
if (is.null(dim(MonthlySummary))) {dim(MonthlySummary) <- c(1,
length(ParameterNames) + 2)}

# Calculate the statistics per year
for (i in min(TTS$Year) : max(TTS$Year)){
        k = i + 1 - min(TTS$Year)
        YearlyOutput[k, 1] = as.numeric(paste(Site[ColNumber], ".", SegNumber,
sep = ""))
        YearlyOutput[k, 2] = i
        YearlyOutput[k, 3] = length(TTS$Temperature[TTS$Year == i])
        YearlyOutput[k, 4] = mean(TTS$Temperature[TTS$Year == i], na.rm = TRUE)
        YearlyOutput[k, 5] = max(TTS$Temperature[TTS$Year == i], na.rm = TRUE)
        YearlyOutput[k, 6] = min(TTS$Temperature[TTS$Year == i], na.rm = TRUE)
        YearlyOutput[k, 7] = YearlyOutput[k, 5] - YearlyOutput[k, 6]
        YearlyOutput[k, 8] = sd(TTS$Temperature[TTS$Year == i])
        YearlyOutput[k, 9] = YearlyOutput[k, 8] / (sqrt(YearlyOutput[k, 3]))
        YearlyOutput[k, 10] = YearlyOutput[k, 8] / YearlyOutput[k, 4]
        YearlyOutput[k, 11] = max(TTS$RateOfChange[TTS$Year == i], na.rm = TRUE)
        YearlyOutput[k, 12] = min(TTS$RateOfChange[TTS$Year == i], na.rm = TRUE)
        YearlyOutput[k, 13] = mean(DailyOutput[, 9][DailyOutput[, 2] == i],
na.rm = TRUE)
        YearlyOutput[k, 14] = max(DailyOutput[, 9][DailyOutput[, 2] == i], na.rm
```

```
= TRUE)
        YearlyOutput[k, 15] = min(DailyOutput[, 9][DailyOutput[, 2] == i], na.rm
= TRUE)
        YearlyOutput[k, 16] = mean(MonthlyOutput[, 8][MonthlyOutput[, 2] == i],
na.rm = TRUE)
        YearlyOutput[k, 17] = max(MonthlyOutput[, 8][MonthlyOutput[, 2] == i],
na.rm = TRUE)
        YearlyOutput[k, 18] = min(MonthlyOutput[, 8][MonthlyOutput[, 2] == i],
na.rm = TRUE)
        YearlyOutput[k, 19] = YearlyOutput[k, 13] / YearlyOutput[k, 7]
        YearlyOutput[k, 20] = quantile(TTS$Temperature[TTS$Year == i], Quantile,
na.rm = TRUE)
        YearlyOutput[k, 21] = quantile(TTS$Temperature[TTS$Year == i], 1 -
Quantile, na.rm = TRUE)
        YearlyOutput[k, 22] = quantile(TTS$RateOfChange[TTS$Year == i],
Quantile, na.rm = TRUE)
        YearlyOutput[k, 23] = quantile(TTS$RateOfChange[TTS$Year == i], 1 -
Quantile, na.rm = TRUE)
        YearlyOutput[k, 24] = quantile(DailyOutput[, 9][DailyOutput[, 2] == i],
Quantile, na.rm = TRUE)
        YearlyOutput[k, 25] = quantile(DailyOutput[, 9][DailyOutput[, 2] == i],
1 - Quantile, na.rm = TRUE)
        YearlyOutput[k, 26] = quantile(MonthlyOutput[, 8][MonthlyOutput[, 2] ==
i], Quantile, na.rm = TRUE)
        YearlyOutput[k, 27] = quantile(MonthlyOutput[, 8][MonthlyOutput[, 2] ==
i], 1 - Quantile, na.rm = TRUE)}


# Calculate the statistics over the entire dataset
DatasetOutput[1, 1] = as.numeric(paste(Site[ColNumber], ".", SegNumber, sep =
""))
DatasetOutput[1, 2] = length(TTS$Temperature)
DatasetOutput[1, 3] = mean(TTS$Temperature, na.rm = TRUE)
DatasetOutput[1, 4] = max(TTS$Temperature)
DatasetOutput[1, 5] = min(TTS$Temperature)
DatasetOutput[1, 6] = DatasetOutput[1, 4] - DatasetOutput[1, 5]
DatasetOutput[1, 7] = sd(TTS$Temperature)
DatasetOutput[1, 8] = DatasetOutput[1, 7] / sqrt(DatasetOutput[1, 2])
DatasetOutput[1, 9] = DatasetOutput[1, 7] / DatasetOutput[1, 3]
DatasetOutput[1, 10] = max(TTS$RateOfChange, na.rm = TRUE)
DatasetOutput[1, 11] = min(TTS$RateOfChange, na.rm = TRUE)
DatasetOutput[1, 12] = mean(DailyOutput[, 9], na.rm = TRUE)
DatasetOutput[1, 13] = max(DailyOutput[, 9], na.rm = TRUE)
DatasetOutput[1, 14] = min(DailyOutput[, 9], na.rm = TRUE)
DatasetOutput[1, 15] = mean(MonthlyOutput[, 8], na.rm = TRUE)
DatasetOutput[1, 16] = max(MonthlyOutput[, 8], na.rm = TRUE)
DatasetOutput[1, 17] = min(MonthlyOutput[, 8], na.rm = TRUE)
DatasetOutput[1, 18] = DatasetOutput[1, 12] / DatasetOutput[1, 6]
DatasetOutput[1, 19] = quantile(TTS$Temperature, Quantile, na.rm = TRUE)
DatasetOutput[1, 20] = quantile(TTS$Temperature, 1 - Quantile, na.rm = TRUE)
DatasetOutput[1, 21] = quantile(TTS$RateOfChange, Quantile, na.rm = TRUE)
DatasetOutput[1, 22] = quantile(TTS$RateOfChange, 1 - Quantile, na.rm = TRUE)
```

```
DatasetOutput[1, 23] = quantile(DailyOutput[, 9], Quantile, na.rm = TRUE)
DatasetOutput[1, 24] = quantile(DailyOutput[, 9], 1 - Quantile, na.rm = TRUE)
DatasetOutput[1, 25] = quantile(MonthlyOutput[, 8], Quantile, na.rm = TRUE)
DatasetOutput[1, 26] = quantile(MonthlyOutput[, 8], 1 - Quantile, na.rm = TRUE)

# Create the relevant output files
if (Do.DatasetOutput == "y") {if (file.exists(paste(OutputFileName,
"_DataSummary.csv", sep = ""))) {write.table(DatasetOutput, file =
paste(OutputFileName, "_DataSummary.csv", sep = ""), append = TRUE, sep = ",",
dec = ".", quote = FALSE, na = "NA", col.names = FALSE, row.names = FALSE)} else
{write.table(DatasetOutput, file = paste(OutputFileName, "_DataSummary.csv", sep
= ""), append = FALSE, sep = ",", dec = ".", quote = FALSE, na = "NA", col.names
= TRUE, row.names = FALSE)}}
if (Do.YearlyOutput == "y") {if (file.exists(paste(OutputFileName,
"_AnnualSummary.csv", sep = ""))) {write.table(YearlyOutput, file =
paste(OutputFileName, "_AnnualSummary.csv", sep = ""), append = TRUE, sep = ",",
dec = ".", quote = FALSE, na = "NA", col.names = FALSE, row.names = FALSE)} else
{write.table(YearlyOutput, file = paste(OutputFileName, "_AnnualSummary.csv",
sep = ""), append = FALSE, sep = ",", dec = ".", quote = FALSE, na = "NA",
col.names = TRUE, row.names = FALSE)}}
if (Do.MonthlyOutput == "y") {if (file.exists(paste(OutputFileName,
"_MonthlySummary.csv", sep = ""))) {write.table(MonthlyOutput[, c(1 : 16, 21 :
26)], file = paste(OutputFileName, "_MonthlySummary.csv", sep = ""), append =
TRUE, sep = ",", dec = ".", quote = FALSE, na = "NA", col.names = FALSE,
row.names = FALSE)} else {write.table(MonthlyOutput[, c(1 : 16, 21 : 26)], file
= paste(OutputFileName, "_MonthlySummary.csv", sep = ""), append = FALSE, sep =
",", dec = ".", quote = FALSE, na = "NA", col.names = TRUE, row.names = FALSE)}}
if (Do.DailyOutput == "y") {if (file.exists(paste(OutputFileName,
"_DailySummary.csv", sep = ""))) {write.table(DailyOutput[, 1 : 14], file =
paste(OutputFileName, "_DailySummary.csv", sep = ""), append = TRUE, sep = ",",
dec = ".", quote = FALSE, na = "NA", col.names = FALSE, row.names = FALSE)} else
{write.table(DailyOutput[, 1 : 14], file = paste(OutputFileName,
"_DailySummary.csv", sep = ""), append = FALSE, sep = ",", dec = ".", quote =
FALSE, na = "NA", col.names = TRUE, row.names = FALSE)}}
if (Do.MonthlySummary == "y") {if (file.exists(paste(OutputFileName,
"_MonthSummary.csv", sep = ""))) {write.table(MonthlySummary[, c(1 : 18, 20 :
27)], file = paste(OutputFileName, "_MonthSummary.csv", sep = ""), append =
TRUE, sep = ",", dec = ".", quote = FALSE, na = "NA", col.names = FALSE,
row.names = FALSE)} else {write.table(MonthlySummary[, c(1 : 18, 20 : 27)], file
= paste(OutputFileName, "_MonthSummary.csv", sep = ""), append = FALSE, sep =
",", dec = ".", quote = FALSE, na = "NA", col.names = TRUE, row.names = FALSE)}}


##############################
# 7a. Create summary figures #
##############################

if (Do.Plot == "y") {
if (exists("Interpolated.TTS") & exists("LongestDuration") &
exists("Interpolated.LongestDuration.TTS")) {

# Plot time series (with/without interpolation)
```

```
tiff(paste(OutputFileName, "_time_series_Site_", Site[ColNumber], ".",
SegNumber, ".tif", sep = ""), units = "cm", height = 24, width = 20, res = 150)
par(mfcol=c(3, 2))

plot(TTS$DateTime, TTS$Temperature, type = "l", ylab = "Temperature", main =
paste("Unmodified time series (n = ", length(TTS[, 1]), "; gaps = ",
length(Gaps) - 2, ")", sep = ""), xlim = c(min(TTS$DateTime),
max(TTS$DateTime)))
if (length(Gaps) > 2) {
for (i in 2 : (length(Gaps) - 1)) {
        x = c(TTS$DateTime[Gaps[i]], TTS$DateTime[Gaps[i]], TTS$DateTime[Gaps[i]
- 1], TTS$DateTime[Gaps[i] - 1])
        y = c(min(TTS$Temperature), max(TTS$Temperature), max(TTS$Temperature),
min(TTS$Temperature))
        polygon(x, y, col = "white", border = "white")}}

plot(LongestDuration$DateTime, LongestDuration$Temperature, type = "l", ylab =
"Temperature", main = paste("Longest duration w/out missing data (n = ",
length(LongestDuration[, 1]), " records)", sep = ""), xlim =
c(min(TTS$DateTime), max(TTS$DateTime)))

plot(TTS$DateTime, TTS$Temperature, type = "l", ylab = "Temperature", main =
paste("Time series split by missing data (segments = ", length(Gaps) - 1,")",
sep = ""), xlim = c(min(TTS$DateTime), max(TTS$DateTime)))
if (length(Gaps) > 2) {
for (i in 2 : (length(Gaps) - 1)) {
        x = c(TTS$DateTime[Gaps[i]], TTS$DateTime[Gaps[i]], TTS$DateTime[Gaps[i]
- 1], TTS$DateTime[Gaps[i] - 1])
        y = c(min(TTS$Temperature), max(TTS$Temperature), max(TTS$Temperature),
min(TTS$Temperature))
        polygon(x, y, col = "white", border = "white")
        abline(v = TTS$DateTime[Gaps - 1], col = "red")}
abline(v = TTS$DateTime[Gaps], col = "red")
for (i in 1 : (length(Gaps) - 1)) {
        text(x = TTS$DateTime[Gaps[i]] + as.numeric(difftime(TTS$DateTime[Gaps[i
+ 1]], TTS$DateTime[Gaps[i]], units = "sec") / 2), y = 0.9 *
min(TTS$Temperature), paste("Seg.", i, sep = ""), cex = 0.9, bg = "white", col =
"red", font = 2)}}

plot(Interpolated.TTS$DateTime, Interpolated.TTS$Temperature, type = "l", ylab =
"Temperature", main = paste("TS with linear interpolation (n = ",
length(Interpolated.TTS[, 1]), ")", sep = ""), xlim = c(min(TTS$DateTime),
max(TTS$DateTime)))
if (length(Gaps) > 2) {
for (i in 2 : (length(Gaps) - 1)) {
        x = c(TTS$DateTime[Gaps[i]], TTS$DateTime[Gaps[i]], TTS$DateTime[Gaps[i]
- 1], TTS$DateTime[Gaps[i] - 1])
        y = c(1.5 * min(TTS$Temperature), 1.5 * max(TTS$Temperature), 1.5 *
max(TTS$Temperature), 1.5 * min(TTS$Temperature))
        polygon(x, y, col = rgb(0, 0, 1, alpha = 0.5), border = "blue")}}

plot(Interpolated.TTS$DateTime, Interpolated.TTS$Temperature, type = "l", ylab =
```

```
"Temperature", main = paste("TS with limited interpolation (segments = ",
length(LongGaps) - 1,")"), xlim = c(min(TTS$DateTime), max(TTS$DateTime)))
if (length(Gaps) > 2) {
for (i in 2 : (length(Gaps) - 1)) {
        x <- c(TTS$DateTime[Gaps[i]], TTS$DateTime[Gaps[i]],
TTS$DateTime[Gaps[i] - 1], TTS$DateTime[Gaps[i] - 1])
        y <- c(1.5 * min(TTS$Temperature), 1.5 * max(TTS$Temperature), 1.5 *
max(TTS$Temperature), 1.5 * min(TTS$Temperature))
        polygon(x, y, col = rgb(0, 0, 1, alpha = 0.5), border = "blue")}
if (length(LongGaps) != 2) {
for (i in 2 : (length(LongGaps) - 1)) {
        x <- c(TTS$DateTime[LongGaps[i]], TTS$DateTime[LongGaps[i]],
TTS$DateTime[LongGaps[i] - 1], TTS$DateTime[LongGaps[i] - 1])
        y <- c(1.5 * min(TTS$Temperature), 1.5 * max(TTS$Temperature), 1.5 *
max(TTS$Temperature), 1.5 * min(TTS$Temperature))
        polygon(x, y, col="white", border="white")}
for (i in 1 : (length(LongGaps) - 1)) {
        text(x = TTS$DateTime[LongGaps[i]] +
as.numeric(difftime(TTS$DateTime[LongGaps[i + 1]], TTS$DateTime[LongGaps[i]],
units = "sec") / 2), y = 0.9 * min(TTS$Temperature), paste("Seg.", i, sep = ""),
cex = 0.9, bg = "white", col = "red", font = 2)}}
if (length(LongGaps) == 2) {text(x = TTS$DateTime[1] +
as.numeric(difftime(TTS$DateTime[length(TTS$DateTime)], TTS$DateTime[1], units =
"sec") / 2), y = 0.9 * min(TTS$Temperature), "Only one segment", cex = 0.9, bg =
"white", col = "red", font = 2)}
box("plot")}

plot(Interpolated.LongestDuration.TTS$DateTime,
Interpolated.LongestDuration.TTS$Temperature, type = "l", ylab = "Temperature",
main = paste("Longest duration with limited interpolation (n = ",
length(Interpolated.LongestDuration.TTS[, 1]), ")", sep = ""), xlim =
c(min(TTS$DateTime), max(TTS$DateTime)))
if (length(Gaps) > 2) {
if (length(ShortGaps) > 2) {
for (i in 2 : (length(ShortGaps) - 1)) {
        Temp.ShortGaps <- ShortGaps + LongGaps.LongestDuration.start
        x = c(TTS$DateTime[Temp.ShortGaps[i]], TTS$DateTime[Temp.ShortGaps[i]],
TTS$DateTime[Temp.ShortGaps[i] - 1], TTS$DateTime[Temp.ShortGaps[i] - 1])
        y = c(1.5 * min(TTS$Temperature), 1.5 * max(TTS$Temperature), 1.5 *
max(TTS$Temperature), 1.5 * min(TTS$Temperature))
        polygon(x, y, col = rgb(0, 0, 1, alpha = 0.5), border = "blue")}
        remove(Temp.ShortGaps)}}
dev.off()}


# Draw a histogram of the rate of change values, annotating with maximum and
minimum rate of change values
tiff(paste(OutputFileName, "_RateOfChange_Site_", Site[ColNumber], ".",
SegNumber, ".tif", sep = ""), units = "cm", height = 8, width = 18, res = 150)
par(mfrow = c(1, 3))
histo <- hist(TTS$RateOfChange, plot = FALSE)
hist(TTS$RateOfChange, xlab = "Rate of temperature range (°C per hour)", ylab =
```

```
"Number of observations", main = "", ylim = c(0, max(histo$count) * 1.1))
abline(h = 0)
plot(histo$mid[abs(log10(histo$counts)) <
Inf],log10(histo$counts)[abs(log10(histo$counts)) < Inf], xlab = "Rate of
temperature range (°C per hour)", ylab = "Number of observations (log 10)", main
= paste(OutputFileName, ": Site ", Site[ColNumber], ".", SegNumber, sep = ""),
type = "h", lwd = 8, lend = 1, ylim = c(0, max(log10(histo$count)) * 1.1), bty =
"n")
abline(h = 0)
plot(1 : 2, 1 : 2, type = "n",xaxt = "n", yaxt = "n", xlab = "", ylab = "", bty
= "n")
legend("center", cex = 0.85, xjust = 0.5, legend = paste("Max. cooling rate = ",
round(min(TTS$RateOfChange), 2), " °C/hour",
        "\nMax. warming rate = ", round(max(TTS$RateOfChange), 2), " °C/hour",
"\n", "\n", 1 - Quantile,"% - ", Quantile, "% quantile range = ",
        "\n", round(quantile(TTS$RateOfChange, 1 - Quantile, na.rm = TRUE), 2),"
: ", round(quantile(TTS$RateOfChange, Quantile, na.rm = TRUE), 2), "
°C/hour\n"), bty = "n")
dev.off()
remove(histo)

# Draw a histogram of the number of records relative to the total number of
possible records for time period (i.e. indicating the proportion of missing data
in the time series based on the average sampling interval).
if (exists("Interpolated.TTS")) {
tiff(paste(OutputFileName, "_SampleSize_monthly_", Site[ColNumber], ".",
SegNumber, ".tif", sep = ""), width = 620)
plot(y=MonthlySummary[, 3], x = MonthlySummary[, 2], type = "h", xaxt = "n", lwd
= 20, lend = 2, ylim = c(0, 1.2 * max(MonthlySummary[, 3])), xlab = "Month",
ylab = "Records")
axis(side = 1,at = 1 : 12, labels = c("J", "F", "M", "A", "M", "J", "J", "A",
"S", "O", "N", "D"))
text(x = 1 : 12, y = 1.1 * max(MonthlySummary[, 3]), labels =
paste(round(((MonthlySummary[, 3] / table(Interpolated.TTS[, 2]) * 100)), 0),
"%", sep = ""))
text(labels = "Percentage of recordings in time series:", adj = 0.5, x = 6.5, y
= 1.175 * max(MonthlySummary[, 3]))
dev.off()

tiff(paste(OutputFileName, "_SampleSize_yearly_", Site[ColNumber], ".",
SegNumber, ".tif", sep = ""), width = 540)
plot(y = YearlyOutput[, 3], x = YearlyOutput[, 2], type = "h", lwd = 20, lend =
2, ylim = c(0, 1.2 * max(YearlyOutput[, 3])), xlab = "Year", ylab = "Records")
text(x = seq(from = min(YearlyOutput[, 2]), to = max(YearlyOutput[, 2]), by =
1), y = 1.1 * max(YearlyOutput[, 3]), labels = paste(round(((YearlyOutput[, 3] /
table(Interpolated.TTS[, 1]) * 100)), 0),"%", sep = ""))
text(labels = "Percentage of recordings in time series:", adj = 0.5, x =
mean(YearlyOutput[, 2]), y = 1.175 * max(YearlyOutput[, 3]))
dev.off()
}
}
```

```
##########################################################################################
# 7b. Calculate annual, monthly and daily summaries, splitting data by day and night #
##########################################################################################

# Calculate sunrise and sunset time for each day based on geographic
co-ordinates (formulas from
http://www.srrb.noaa.gov/highlights/sunrise/calcdetails.html)
# For this section, the following notation is used: 0 = Night; 1 = Day

# Check if analyses must be run.
if (Do.DayNightOutput == "y") {

# Calculate sunrise and sunset for each day, and classify all recordings as day
or night
DayMatrix <- matrix(ncol = 3, nrow = length(TTS$Year))
colnames(DayMatrix) <- c("Sunrise", "Sunset", "DayOrNight")
sunrise <- vector(length = length(TTS$Year))
sunset <- vector(length = length(TTS$Year))
for (i in 1 : length(TTS$Year)) {
        Fractional.Year <- 2 * (pi / 365) * DateTime$yday[i]
        eqtime <- 229.18 * (0.000075 + 0.001868 * cos(Fractional.Year) -
0.032077 * sin(Fractional.Year) - 0.014615 * cos(2 * Fractional.Year) - 0.040849
* sin(2 * Fractional.Year))
        decl <- (0.006918 - 0.399912 * cos(Fractional.Year) + 0.070257 *
sin(Fractional.Year) - 0.006758 * cos(2 * Fractional.Year) + 0.000907 * sin(2 *
Fractional.Year) - 0.002697 * cos(3 * Fractional.Year) + 0.00148 * sin(3 *
Fractional.Year))
        time.offset <- eqtime - (4 * Long[ColNumber]) + (60 *
TimeZone[ColNumber])
        tst <- (DateTime$hour[i] * 60) + DateTime$min[i] + (DateTime$sec[i] /
60) + time.offset
        ha <- (cos(90.833 * pi / 180) / (cos(Lat[ColNumber] * pi / 180) *
cos(decl))) - tan(Lat[ColNumber] * pi / 180) * tan(decl)
        sunrise[i] <- 720 + 4 * (Long[ColNumber] - (acos(ha) * 180 / pi)) -
eqtime - (TimeZone[ColNumber] * 60)
        DayMatrix[i, 1] <- round(sunrise[i] / 60, 2)
        sunset[i] <- 720 + 4 * (Long[ColNumber] + (acos(ha) * 180 / pi)) -
eqtime - (TimeZone[ColNumber] * 60)
        DayMatrix[i, 2] <- round(sunset[i] / 60, 2)
        if (as.numeric(DateTime$hour[i] * 60 + DateTime$min[i]) < sunrise[i] |
as.numeric(DateTime$hour[i] * 60 + DateTime$min[i]) > sunset[i]) {DayMatrix[i,
3] = 0} else {DayMatrix[i, 3] = 1}}
TTS <- cbind(TTS,DayMatrix)
remove(Fractional.Year,eqtime, time.offset, tst, ha, sunrise, DayMatrix, sunset)

# Calculate statistics per year, month, day and day/night (following the same
approach used at the start of section 7 of this code)
ParameterNames <- c("n", "Mean", "Maximum", "Minimum", "Range", "SD", "SE",
```

```
"C.V.", "MaxRoC", "MinRoC", "MeanDTR", "MaxDTR", "MinDTR", "MeanMTR", "MaxMTR",
"MinMTR", "Isotherm.", paste(Quantile * 100, "% Qu. Temp"), paste((1 - Quantile)
* 100, "% Qu. Temp"), paste(Quantile * 100, "% RoC"), paste((1 - Quantile) *
100, "% RoC"), paste(Quantile * 100, "% DTR"), paste((1 - Quantile) * 100, "%
DTR"), paste(Quantile * 100, "% MTR"), paste((1 - Quantile) * 100, "% MTR"))
DatasetDayNightOutput <- matrix(ncol = length(ParameterNames) + 2, nrow = 2)
YearlyDayNightOutput <- matrix(ncol = length(ParameterNames) + 3, nrow = 2 * (1
+ max(TTS$Year) - min(TTS$Year)))
MonthlyDayNightOutput <- matrix(ncol = length(ParameterNames) + 4, nrow = 2 * 12
* (1 + max(TTS$Year) - min(TTS$Year)))
MonthlyDayNightSummary <- matrix(ncol = length(ParameterNames) + 3, nrow = 24)
DailyDayNightOutput <- matrix(ncol = length(ParameterNames) + 5, nrow = 2 *
length(unique(paste(TTS$Year, TTS$Month, TTS$Day))))
colnames(DatasetDayNightOutput) <- c("Site", "DayNight", ParameterNames)
colnames(YearlyDayNightOutput) <- c("Site", "Year", "DayNight", ParameterNames)
colnames(MonthlyDayNightOutput) <- c("Site", "Year", "Month", "DayNight",
ParameterNames)
colnames(DailyDayNightOutput) <- c("Site", "Year", "Month", "Day", "DayNight",
ParameterNames)
colnames(MonthlyDayNightSummary) <- c("Site", "Month", "DayNight",
ParameterNames)
remove(ParameterNames)


# Repeat calculations per year, month and day
k = 1
for (i in 1 : length(DailyOutput[, 1])) {
        q = 0
                DailyDayNightOutput[k, 1] = as.numeric(paste(Site[ColNumber],
".", SegNumber, sep = ""))
                DailyDayNightOutput[k, 2] = DailyOutput[i, 2]
                DailyDayNightOutput[k, 3] = DailyOutput[i, 3]
                DailyDayNightOutput[k, 4] = DailyOutput[i, 4]
                DailyDayNightOutput[k, 5] = q
                DailyDayNightOutput[k, 6] = length(TTS$Temperature[TTS$Year ==
DailyOutput[i, 2] & TTS$Month == DailyOutput[i, 3] & TTS$Day == DailyOutput[i,
4] & TTS$DayOrNight == q])
                DailyDayNightOutput[k, 7] = mean(TTS$Temperature[TTS$Year ==
DailyOutput[i, 2] & TTS$Month == DailyOutput[i, 3] & TTS$Day == DailyOutput[i,
4] & TTS$DayOrNight == q])
                DailyDayNightOutput[k, 8] = max(TTS$Temperature[TTS$Year ==
DailyOutput[i, 2] & TTS$Month == DailyOutput[i, 3] & TTS$Day == DailyOutput[i,
4] & TTS$DayOrNight == q])
                DailyDayNightOutput[k, 9] = min(TTS$Temperature[TTS$Year ==
DailyOutput[i, 2] & TTS$Month == DailyOutput[i, 3] & TTS$Day == DailyOutput[i,
4] & TTS$DayOrNight == q])
                if (DailyDayNightOutput[k, 6]<MinDNRecords)
{DailyDayNightOutput[k, 10] = NA} else {DailyDayNightOutput[k, 10] =
DailyDayNightOutput[k, 8] - DailyDayNightOutput[k, 9]}
                DailyDayNightOutput[k, 11] = sd(TTS$Temperature[TTS$Year ==
DailyOutput[i, 2] & TTS$Month == DailyOutput[i, 3] & TTS$Day == DailyOutput[i,
4] & TTS$DayOrNight == q])
```

```
                DailyDayNightOutput[k, 12] = DailyDayNightOutput[k, 11] / 
sqrt(DailyDayNightOutput[k, 6])
                DailyDayNightOutput[k, 13] = DailyDayNightOutput[k, 11] / 
DailyDayNightOutput[k, 7]
                if (DailyDayNightOutput[k, 6] < MinDNRecords) 
{DailyDayNightOutput[k, 14] = NA} else {DailyDayNightOutput[k, 14] = 
max(TTS$RateOfChange[TTS$Year == DailyOutput[i, 2] & TTS$Month == DailyOutput[i, 
3] & TTS$Day == DailyOutput[i, 4] & TTS$DayOrNight == q])}
                if (DailyDayNightOutput[k, 6] < MinDNRecords) 
{DailyDayNightOutput[k, 15] = NA} else {DailyDayNightOutput[k, 15] = 
min(TTS$RateOfChange[TTS$Year == DailyOutput[i, 2] & TTS$Month == DailyOutput[i, 
3] & TTS$Day == DailyOutput[i, 4] & TTS$DayOrNight == q])}
                # columns 16 - 22 reserved for DTR and MTR and Isotherm.
        k = k + 1
        q = 1
                DailyDayNightOutput[k, 1] = as.numeric(paste(Site[ColNumber], 
".", SegNumber, sep = ""))
                DailyDayNightOutput[k, 2] = DailyOutput[i, 2]
                DailyDayNightOutput[k, 3] = DailyOutput[i, 3]
                DailyDayNightOutput[k, 4] = DailyOutput[i, 4]
                DailyDayNightOutput[k, 5] = q
                DailyDayNightOutput[k, 6] = length(TTS$Temperature[TTS$Year == 
DailyOutput[i, 2] & TTS$Month == DailyOutput[i, 3] & TTS$Day == DailyOutput[i, 
4] & TTS$DayOrNight == q])
                DailyDayNightOutput[k, 7] = mean(TTS$Temperature[TTS$Year == 
DailyOutput[i, 2] & TTS$Month == DailyOutput[i, 3] & TTS$Day == DailyOutput[i, 
4] & TTS$DayOrNight == q])
                DailyDayNightOutput[k, 8] = max(TTS$Temperature[TTS$Year == 
DailyOutput[i, 2] & TTS$Month == DailyOutput[i, 3] & TTS$Day == DailyOutput[i, 
4] & TTS$DayOrNight == q])
                DailyDayNightOutput[k, 9] = min(TTS$Temperature[TTS$Year == 
DailyOutput[i, 2] & TTS$Month == DailyOutput[i, 3] & TTS$Day == DailyOutput[i, 
4] & TTS$DayOrNight == q])
                if (DailyDayNightOutput[k, 6] < MinDNRecords) 
{DailyDayNightOutput[k, 10] = NA} else {DailyDayNightOutput[k, 10] = 
DailyDayNightOutput[k, 8] - DailyDayNightOutput[k, 9]}
                DailyDayNightOutput[k, 11] = sd(TTS$Temperature[TTS$Year == 
DailyOutput[i, 2] & TTS$Month == DailyOutput[i, 3] & TTS$Day == DailyOutput[i, 
4] & TTS$DayOrNight == q])
                DailyDayNightOutput[k, 12] = DailyDayNightOutput[k, 11] / 
sqrt(DailyDayNightOutput[k, 6])
                DailyDayNightOutput[k, 13] = DailyDayNightOutput[k, 11] / 
DailyDayNightOutput[k, 7]
                if (DailyDayNightOutput[k, 6] < MinDNRecords) 
{DailyDayNightOutput[k, 14] = NA} else {DailyDayNightOutput[k, 14] = 
max(TTS$RateOfChange[TTS$Year == DailyOutput[i, 2] & TTS$Month == DailyOutput[i, 
3] & TTS$Day == DailyOutput[i, 4] & TTS$DayOrNight == q])}
                if (DailyDayNightOutput[k, 6] < MinDNRecords) 
{DailyDayNightOutput[k, 15] = NA} else {DailyDayNightOutput[k, 15] = 
min(TTS$RateOfChange[TTS$Year == DailyOutput[i, 2] & TTS$Month == DailyOutput[i, 
3] & TTS$Day == DailyOutput[i, 4] & TTS$DayOrNight == q])}
        k = k + 1}
```

```
# Repeat calculations per year and month
k = 1
for (i in 1:length(MonthlyOutput[, 1])) {
        q = 0
                MonthlyDayNightOutput[k, 1] = as.numeric(paste(Site[ColNumber],
".", SegNumber, sep = ""))
                MonthlyDayNightOutput[k, 2] = MonthlyOutput[i, 2]
                MonthlyDayNightOutput[k, 3] = MonthlyOutput[i, 3]
                MonthlyDayNightOutput[k, 4] = q
                MonthlyDayNightOutput[k, 5] = length(TTS$Temperature[TTS$Year ==
MonthlyOutput[i, 2] & TTS$Month == MonthlyOutput[i, 3] & TTS$DayOrNight == q])
                MonthlyDayNightOutput[k, 6] = mean(TTS$Temperature[TTS$Year ==
MonthlyOutput[i, 2] & TTS$Month == MonthlyOutput[i,3] & TTS$DayOrNight == q])
                MonthlyDayNightOutput[k, 7] = max(TTS$Temperature[TTS$Year ==
MonthlyOutput[i, 2] & TTS$Month == MonthlyOutput[i,3] & TTS$DayOrNight == q])
                MonthlyDayNightOutput[k, 8] = min(TTS$Temperature[TTS$Year ==
MonthlyOutput[i, 2] & TTS$Month == MonthlyOutput[i,3] & TTS$DayOrNight == q])
                MonthlyDayNightOutput[k, 9] = MonthlyDayNightOutput[k, 7] -
MonthlyDayNightOutput[k, 8]
                MonthlyDayNightOutput[k, 10] = sd(TTS$Temperature[TTS$Year ==
MonthlyOutput[i, 2] & TTS$Month == MonthlyOutput[i,3] & TTS$DayOrNight == q])
                MonthlyDayNightOutput[k, 11] = MonthlyDayNightOutput[k, 10] /
sqrt(MonthlyDayNightOutput[k, 5])
                MonthlyDayNightOutput[k, 12] = MonthlyDayNightOutput[k, 10] /
MonthlyDayNightOutput[k, 6]
                MonthlyDayNightOutput[k, 13] = max(TTS$RateOfChange[TTS$Year ==
MonthlyOutput[i, 2] & TTS$Month == MonthlyOutput[i,3] & TTS$DayOrNight == q])
                MonthlyDayNightOutput[k, 14] = min(TTS$RateOfChange[TTS$Year ==
MonthlyOutput[i, 2] & TTS$Month == MonthlyOutput[i,3] & TTS$DayOrNight == q])
                MonthlyDayNightOutput[k, 15] = mean(DailyDayNightOutput[,
10][DailyDayNightOutput[, 2] == MonthlyOutput[i,2] & DailyDayNightOutput[, 3] ==
MonthlyOutput[i, 3] & DailyDayNightOutput[, 5] == q], na.rm = TRUE)
                MonthlyDayNightOutput[k, 16] = max(DailyDayNightOutput[,
10][DailyDayNightOutput[, 2] == MonthlyOutput[i,2] & DailyDayNightOutput[, 3] ==
MonthlyOutput[i, 3] & DailyDayNightOutput[, 5] == q], na.rm = TRUE)
                MonthlyDayNightOutput[k, 17] = min(DailyDayNightOutput[,
10][DailyDayNightOutput[, 2] == MonthlyOutput[i,2] & DailyDayNightOutput[, 3] ==
MonthlyOutput[i, 3] & DailyDayNightOutput[, 5] == q], na.rm = TRUE)
                #MonthlyOutput[k, 18 - 21] reserved
                MonthlyDayNightOutput[k, 22] = quantile(TTS$Temperature[TTS$Year
== MonthlyOutput[i, 2] & TTS$Month == MonthlyOutput[i, 3] & TTS$DayOrNight ==
q], Quantile, na.rm = TRUE)
                MonthlyDayNightOutput[k, 23] = quantile(TTS$Temperature[TTS$Year
== MonthlyOutput[i, 2] & TTS$Month == MonthlyOutput[i, 3] & TTS$DayOrNight ==
q], 1 - Quantile, na.rm = TRUE)
                MonthlyDayNightOutput[k, 24] =
quantile(TTS$RateOfChange[TTS$Year == MonthlyOutput[i, 2] & TTS$Month ==
MonthlyOutput[i, 3] & TTS$DayOrNight == q], Quantile, na.rm = TRUE)
                MonthlyDayNightOutput[k, 25] =
quantile(TTS$RateOfChange[TTS$Year == MonthlyOutput[i, 2] & TTS$Month ==
```

```
MonthlyOutput[i, 3] & TTS$DayOrNight == q], 1 - Quantile, na.rm = TRUE)
                MonthlyDayNightOutput[k, 26] = quantile(DailyDayNightOutput[, 
10][DailyDayNightOutput[, 2] == MonthlyOutput[i, 2] & DailyDayNightOutput[, 3] 
== MonthlyOutput[i, 3] & DailyDayNightOutput[, 5] == q], Quantile, na.rm = TRUE)
                MonthlyDayNightOutput[k, 27] = quantile(DailyDayNightOutput[, 
10][DailyDayNightOutput[, 2] == MonthlyOutput[i, 2] & DailyDayNightOutput[, 3] 
== MonthlyOutput[i, 3] & DailyDayNightOutput[, 5] == q], 1 - Quantile, na.rm = 
TRUE)
        k = k+1
        q = 1
                MonthlyDayNightOutput[k, 1] = as.numeric(paste(Site[ColNumber], 
".", SegNumber, sep = ""))
                MonthlyDayNightOutput[k, 2] = MonthlyOutput[i, 2]
                MonthlyDayNightOutput[k, 3] = MonthlyOutput[i, 3]
                MonthlyDayNightOutput[k, 4] = q
                MonthlyDayNightOutput[k, 5] = length(TTS$Temperature[TTS$Year == 
MonthlyOutput[i, 2] & TTS$Month == MonthlyOutput[i, 3]  & TTS$DayOrNight == q])
                MonthlyDayNightOutput[k, 6] = mean(TTS$Temperature[TTS$Year == 
MonthlyOutput[i, 2] & TTS$Month == MonthlyOutput[i, 3]  & TTS$DayOrNight == q])
                MonthlyDayNightOutput[k, 7] = max(TTS$Temperature[TTS$Year == 
MonthlyOutput[i, 2] & TTS$Month == MonthlyOutput[i, 3]  & TTS$DayOrNight == q])
                MonthlyDayNightOutput[k, 8] = min(TTS$Temperature[TTS$Year == 
MonthlyOutput[i, 2] & TTS$Month == MonthlyOutput[i, 3]  & TTS$DayOrNight == q])
                MonthlyDayNightOutput[k, 9] = MonthlyDayNightOutput[k, 7] - 
MonthlyDayNightOutput[k, 8]
                MonthlyDayNightOutput[k, 10] = sd(TTS$Temperature[TTS$Year == 
MonthlyOutput[i, 2] & TTS$Month == MonthlyOutput[i, 3] & TTS$DayOrNight == q])
                MonthlyDayNightOutput[k, 11] = MonthlyDayNightOutput[k, 10] / 
sqrt(MonthlyDayNightOutput[k, 5])
                MonthlyDayNightOutput[k, 12] = MonthlyDayNightOutput[k, 10] / 
MonthlyDayNightOutput[k, 6]
                MonthlyDayNightOutput[k, 13] = max(TTS$RateOfChange[TTS$Year == 
MonthlyOutput[i, 2] & TTS$Month == MonthlyOutput[i, 3] & TTS$DayOrNight == q])
                MonthlyDayNightOutput[k, 14] = min(TTS$RateOfChange[TTS$Year == 
MonthlyOutput[i, 2] & TTS$Month == MonthlyOutput[i, 3] & TTS$DayOrNight == q])
                MonthlyDayNightOutput[k, 15] = mean(DailyDayNightOutput[, 
10][DailyDayNightOutput[, 2] == MonthlyOutput[i, 2] & DailyDayNightOutput[, 3] 
== MonthlyOutput[i, 3] & DailyDayNightOutput[, 5] == q], na.rm = TRUE)
                MonthlyDayNightOutput[k, 16] = max(DailyDayNightOutput[, 
10][DailyDayNightOutput[, 2] == MonthlyOutput[i, 2] & DailyDayNightOutput[, 3] 
== MonthlyOutput[i, 3] & DailyDayNightOutput[, 5] == q], na.rm = TRUE)
                MonthlyDayNightOutput[k, 17] = min(DailyDayNightOutput[, 
10][DailyDayNightOutput[, 2] == MonthlyOutput[i, 2] & DailyDayNightOutput[, 3] 
== MonthlyOutput[i, 3] & DailyDayNightOutput[, 5] == q], na.rm = TRUE)
                MonthlyDayNightOutput[k, 22] = quantile(TTS$Temperature[TTS$Year 
== MonthlyOutput[i, 2] & TTS$Month == MonthlyOutput[i, 3] & TTS$DayOrNight == 
q], Quantile, na.rm = TRUE)
                MonthlyDayNightOutput[k, 23] = quantile(TTS$Temperature[TTS$Year 
== MonthlyOutput[i, 2] & TTS$Month == MonthlyOutput[i, 3] & TTS$DayOrNight == 
q], 1 - Quantile, na.rm = TRUE)
                MonthlyDayNightOutput[k, 24] = 
quantile(TTS$RateOfChange[TTS$Year == MonthlyOutput[i, 2] & TTS$Month == 
```

```
MonthlyOutput[i, 3] & TTS$DayOrNight == q], Quantile, na.rm = TRUE)
                MonthlyDayNightOutput[k, 25] =
quantile(TTS$RateOfChange[TTS$Year == MonthlyOutput[i, 2] & TTS$Month ==
MonthlyOutput[i, 3] & TTS$DayOrNight == q], 1 - Quantile, na.rm = TRUE)
                MonthlyDayNightOutput[k, 26] = quantile(DailyDayNightOutput[,
10][DailyDayNightOutput[, 2] == MonthlyOutput[i, 2] & DailyDayNightOutput[, 3]
== MonthlyOutput[i, 3] & DailyDayNightOutput[, 5] == q], Quantile, na.rm = TRUE)
                MonthlyDayNightOutput[k, 27] = quantile(DailyDayNightOutput[,
10][DailyDayNightOutput[, 2] == MonthlyOutput[i, 2] & DailyDayNightOutput[, 3]
== MonthlyOutput[i, 3] & DailyDayNightOutput[, 5] == q], 1 - Quantile, na.rm =
TRUE)
        k = k + 1}


# Repeat calculation per month only
k = 1
for (i in 1 : 12) {
q = 0
        MonthlyDayNightSummary[k, 1] = as.numeric(paste(Site[ColNumber], ".",
SegNumber, sep = ""))
        MonthlyDayNightSummary[k, 2] = i
        MonthlyDayNightSummary[k, 3] = q
        MonthlyDayNightSummary[k, 4] = length(TTS$Temperature[TTS$Month == i &
TTS$DayOrNight == q])
        MonthlyDayNightSummary[k, 5] = mean(TTS$Temperature[TTS$Month == i &
TTS$DayOrNight == q], na.rm = TRUE)
        MonthlyDayNightSummary[k, 6] = max(TTS$Temperature[TTS$Month == i &
TTS$DayOrNight == q], na.rm = TRUE)
        MonthlyDayNightSummary[k, 7] = min(TTS$Temperature[TTS$Month == i &
TTS$DayOrNight == q], na.rm = TRUE)
        MonthlyDayNightSummary[k, 8] = MonthlyDayNightSummary[k, 6] -
MonthlyDayNightSummary[k, 7]
        MonthlyDayNightSummary[k, 9] = sd(TTS$Temperature[TTS$Month == i &
TTS$DayOrNight == q])
        MonthlyDayNightSummary[k, 10] = MonthlyDayNightSummary[k, 9] /
sqrt(MonthlyDayNightSummary[k, 4])
        MonthlyDayNightSummary[k, 11] = MonthlyDayNightSummary[k, 9] /
MonthlyDayNightSummary[k, 5]
        MonthlyDayNightSummary[k, 12] = max(TTS$RateOfChange[TTS$Month == i &
TTS$DayOrNight == q], na.rm = TRUE)
        MonthlyDayNightSummary[k, 13] = min(TTS$RateOfChange[TTS$Month == i &
TTS$DayOrNight == q], na.rm = TRUE)
        MonthlyDayNightSummary[k, 14] = mean(DailyDayNightOutput[,
10][DailyDayNightOutput[, 3] == i & DailyDayNightOutput[, 5] == q], na.rm =
TRUE)
        MonthlyDayNightSummary[k, 15] = max(DailyDayNightOutput[,
10][DailyDayNightOutput[, 3] == i & DailyDayNightOutput[, 5] == q], na.rm =
TRUE)
        MonthlyDayNightSummary[k, 16] = min(DailyDayNightOutput[,
10][DailyDayNightOutput[, 3] == i & DailyDayNightOutput[, 5] == q], na.rm =
TRUE)
        MonthlyDayNightSummary[k, 17] = mean(MonthlyDayNightOutput[,
```

```
9][MonthlyDayNightOutput[, 3] == i & MonthlyDayNightOutput[, 4] == q &
is.na(MonthlyDayNightOutput[, 5]) == FALSE])
        MonthlyDayNightSummary[k, 18] = max(MonthlyDayNightOutput[,
9][MonthlyDayNightOutput[, 3] == i & MonthlyDayNightOutput[, 4] == q &
is.na(MonthlyDayNightOutput[, 5]) == FALSE])
        MonthlyDayNightSummary[k, 19] = min(MonthlyDayNightOutput[,
9][MonthlyDayNightOutput[, 3] == i & MonthlyDayNightOutput[, 4] == q &
is.na(MonthlyDayNightOutput[, 5]) == FALSE])
        MonthlyDayNightSummary[k, 21] = quantile(TTS$Temperature[TTS$Month == i
& TTS$DayOrNight == q], na.rm = TRUE, Quantile)
        MonthlyDayNightSummary[k, 22] = quantile(TTS$Temperature[TTS$Month == i
& TTS$DayOrNight == q], na.rm = TRUE, 1 - Quantile)
        MonthlyDayNightSummary[k, 23] = quantile(TTS$RateOfChange[TTS$Month == i
& TTS$DayOrNight == q], Quantile, na.rm = TRUE)
        MonthlyDayNightSummary[k, 24] = quantile(TTS$RateOfChange[TTS$Month == i
& TTS$DayOrNight == q], 1 - Quantile, na.rm = TRUE)
        MonthlyDayNightSummary[k, 25] = quantile(DailyDayNightOutput[,
10][DailyDayNightOutput[, 3] == i & DailyDayNightOutput[,5] == q], Quantile,
na.rm = TRUE)
        MonthlyDayNightSummary[k, 26] = quantile(DailyDayNightOutput[,
10][DailyDayNightOutput[, 3] == i & DailyDayNightOutput[,5] == q], 1 - Quantile,
na.rm = TRUE)
        MonthlyDayNightSummary[k, 27] = quantile(MonthlyDayNightOutput[,
9][MonthlyDayNightOutput[, 3] == i & MonthlyDayNightOutput[, 4] == q], Quantile,
na.rm = TRUE)
        MonthlyDayNightSummary[k, 28] = quantile(MonthlyDayNightOutput[,
9][MonthlyDayNightOutput[, 3] == i & MonthlyDayNightOutput[, 4] == q], 1 -
Quantile, na.rm = TRUE)
k = k + 1
q = 1
        MonthlyDayNightSummary[k, 1] = as.numeric(paste(Site[ColNumber], ".",
SegNumber, sep = ""))
        MonthlyDayNightSummary[k, 2] = i
        MonthlyDayNightSummary[k, 3] = q
        MonthlyDayNightSummary[k, 4] = length(TTS$Temperature[TTS$Month == i &
TTS$DayOrNight == q])
        MonthlyDayNightSummary[k, 5] = mean(TTS$Temperature[TTS$Month == i &
TTS$DayOrNight == q], na.rm = TRUE)
        MonthlyDayNightSummary[k, 6] = max(TTS$Temperature[TTS$Month == i &
TTS$DayOrNight == q], na.rm = TRUE)
        MonthlyDayNightSummary[k, 7] = min(TTS$Temperature[TTS$Month == i &
TTS$DayOrNight == q], na.rm = TRUE)
        MonthlyDayNightSummary[k, 8] = MonthlyDayNightSummary[k, 6] -
MonthlyDayNightSummary[k, 7]
        MonthlyDayNightSummary[k, 9] = sd(TTS$Temperature[TTS$Month == i &
TTS$DayOrNight == q])
        MonthlyDayNightSummary[k, 10] = MonthlyDayNightSummary[k, 9] /
sqrt(MonthlyDayNightSummary[k, 4])
        MonthlyDayNightSummary[k, 11] = MonthlyDayNightSummary[k, 9] /
MonthlyDayNightSummary[k, 5]
        MonthlyDayNightSummary[k, 12] = max(TTS$RateOfChange[TTS$Month == i &
TTS$DayOrNight == q], na.rm = TRUE)
```

```
        MonthlyDayNightSummary[k, 13] = min(TTS$RateOfChange[TTS$Month == i &
TTS$DayOrNight == q], na.rm = TRUE)
        MonthlyDayNightSummary[k, 14] = mean(DailyDayNightOutput[,
10][DailyDayNightOutput[, 3] == i & DailyDayNightOutput[, 5] == q], na.rm =
TRUE)
        MonthlyDayNightSummary[k, 15] = max(DailyDayNightOutput[,
10][DailyDayNightOutput[, 3] == i & DailyDayNightOutput[, 5] == q], na.rm =
TRUE)
        MonthlyDayNightSummary[k, 16] = min(DailyDayNightOutput[,
10][DailyDayNightOutput[, 3] == i & DailyDayNightOutput[, 5] == q], na.rm =
TRUE)
        MonthlyDayNightSummary[k, 17] = mean(MonthlyDayNightOutput[,
9][MonthlyDayNightOutput[, 3] == i & MonthlyDayNightOutput[, 4] == q &
is.na(MonthlyDayNightOutput[, 5]) == FALSE])
        MonthlyDayNightSummary[k, 18] = max(MonthlyDayNightOutput[,
9][MonthlyDayNightOutput[, 3] == i & MonthlyDayNightOutput[, 4] == q &
is.na(MonthlyDayNightOutput[, 5]) == FALSE])
        MonthlyDayNightSummary[k, 19] = min(MonthlyDayNightOutput[,
9][MonthlyDayNightOutput[, 3] == i & MonthlyDayNightOutput[, 4] == q &
is.na(MonthlyDayNightOutput[, 5]) == FALSE])
        MonthlyDayNightSummary[k, 21] = quantile(TTS$Temperature[TTS$Month == i
& TTS$DayOrNight == q], na.rm = TRUE, Quantile)
        MonthlyDayNightSummary[k, 22] = quantile(TTS$Temperature[TTS$Month == i
& TTS$DayOrNight == q], na.rm = TRUE, 1 - Quantile)
        MonthlyDayNightSummary[k, 23] = quantile(TTS$RateOfChange[TTS$Month == i
& TTS$DayOrNight == q], Quantile, na.rm = TRUE)
        MonthlyDayNightSummary[k, 24] = quantile(TTS$RateOfChange[TTS$Month == i
& TTS$DayOrNight == q], 1-Quantile, na.rm = TRUE)
        MonthlyDayNightSummary[k, 25] = quantile(DailyDayNightOutput[,
10][DailyDayNightOutput[, 3] == i & DailyDayNightOutput[, 5] == q], Quantile,
na.rm = TRUE)
        MonthlyDayNightSummary[k, 26] = quantile(DailyDayNightOutput[,
10][DailyDayNightOutput[, 3] == i & DailyDayNightOutput[, 5] == q], 1 -
Quantile, na.rm = TRUE)
        MonthlyDayNightSummary[k, 27] = quantile(MonthlyDayNightOutput[,
9][MonthlyDayNightOutput[, 3] == i & MonthlyDayNightOutput[, 4] == q], Quantile,
na.rm = TRUE)
        MonthlyDayNightSummary[k, 28] = quantile(MonthlyDayNightOutput[,
9][MonthlyDayNightOutput[, 3] == i & MonthlyDayNightOutput[, 4] == q], 1 -
Quantile, na.rm = TRUE)
k = k + 1}

# Repeat calculations per year
k = 1
for (i in 1 : length(YearlyOutput[, 1])) {
           q = 0
                YearlyDayNightOutput[k, 1] = as.numeric(paste(Site[ColNumber],
".", SegNumber, sep = ""))
                YearlyDayNightOutput[k, 2] = YearlyOutput[i, 2]
                YearlyDayNightOutput[k, 3] = q
                YearlyDayNightOutput[k, 4] = length(TTS$Temperature[TTS$Year ==
YearlyOutput[i, 2] & TTS$DayOrNight == q])
```

```
                YearlyDayNightOutput[k, 5] = mean(TTS$Temperature[TTS$Year ==
YearlyOutput[i, 2] & TTS$DayOrNight == q])
                YearlyDayNightOutput[k, 6] = max(TTS$Temperature[TTS$Year ==
YearlyOutput[i, 2] & TTS$DayOrNight == q])
                YearlyDayNightOutput[k, 7] = min(TTS$Temperature[TTS$Year ==
YearlyOutput[i ,2] & TTS$DayOrNight == q])
                YearlyDayNightOutput[k, 8] = YearlyDayNightOutput[k, 6] -
YearlyDayNightOutput[k, 7]
                YearlyDayNightOutput[k, 9] = sd(TTS$Temperature[TTS$Year ==
YearlyOutput[i, 2] & TTS$DayOrNight == q])
                YearlyDayNightOutput[k, 10] = YearlyDayNightOutput[k, 9] /
sqrt(YearlyDayNightOutput[k, 4])
                YearlyDayNightOutput[k, 11] = YearlyDayNightOutput[k, 9] /
YearlyDayNightOutput[k, 5]
                YearlyDayNightOutput[k, 12] = max(TTS$RateOfChange[TTS$Year ==
YearlyOutput[i, 2] & TTS$DayOrNight == q])
                YearlyDayNightOutput[k, 13] = min(TTS$RateOfChange[TTS$Year ==
YearlyOutput[i, 2] & TTS$DayOrNight == q])
                YearlyDayNightOutput[k, 14] = mean(DailyDayNightOutput[,
10][DailyDayNightOutput[, 2] == YearlyOutput[i, 2] & DailyDayNightOutput[, 5] ==
q], na.rm = TRUE)
                YearlyDayNightOutput[k, 15] = max(DailyDayNightOutput[,
10][DailyDayNightOutput[, 2] == YearlyOutput[i, 2] & DailyDayNightOutput[, 5] ==
q], na.rm = TRUE)
                YearlyDayNightOutput[k, 16] = min(DailyDayNightOutput[,
10][DailyDayNightOutput[, 2] == YearlyOutput[i, 2] & DailyDayNightOutput[, 5] ==
q], na.rm = TRUE)
                YearlyDayNightOutput[k, 17] = mean(MonthlyDayNightOutput[,
9][MonthlyDayNightOutput[, 2] == YearlyOutput[i, 2] & MonthlyDayNightOutput[, 4]
== q], na.rm = TRUE)
                YearlyDayNightOutput[k, 18] = max(MonthlyDayNightOutput[,
9][MonthlyDayNightOutput[, 2] == YearlyOutput[i, 2] & MonthlyDayNightOutput[, 4]
== q], na.rm = TRUE)
                YearlyDayNightOutput[k, 19] = min(MonthlyDayNightOutput[,
9][MonthlyDayNightOutput[, 2] == YearlyOutput[i, 2] & MonthlyDayNightOutput[, 4]
== q], na.rm = TRUE)
                YearlyDayNightOutput[k, 20] = YearlyDayNightOutput[k, 14] /
YearlyDayNightOutput[k, 8]
                YearlyDayNightOutput[k, 21] = quantile(TTS$Temperature[TTS$Year
== YearlyOutput[i, 2] & TTS$DayOrNight == q], Quantile, na.rm = TRUE)
                YearlyDayNightOutput[k, 22] = quantile(TTS$Temperature[TTS$Year
== YearlyOutput[i, 2] & TTS$DayOrNight == q], 1 - Quantile, na.rm = TRUE)
                YearlyDayNightOutput[k, 23] = quantile(TTS$RateOfChange[TTS$Year
== YearlyOutput[i, 2] & TTS$DayOrNight == q], Quantile, na.rm = TRUE)
                YearlyDayNightOutput[k, 24] = quantile(TTS$RateOfChange[TTS$Year
== YearlyOutput[i, 2] & TTS$DayOrNight == q], 1 - Quantile, na.rm = TRUE)
                YearlyDayNightOutput[k, 25] = quantile(DailyDayNightOutput[,
10][DailyDayNightOutput[, 2] == YearlyOutput[i, 2] & DailyDayNightOutput[, 5] ==
q], Quantile, na.rm = TRUE)
                YearlyDayNightOutput[k, 26] = quantile(DailyDayNightOutput[,
10][DailyDayNightOutput[, 2] == YearlyOutput[i, 2] & DailyDayNightOutput[, 5] ==
q], 1 - Quantile, na.rm = TRUE)
```

```
                YearlyDayNightOutput[k, 27] = quantile(MonthlyDayNightOutput[,
9][MonthlyDayNightOutput[, 2] == YearlyOutput[i, 2] & MonthlyDayNightOutput[, 4]
== q], Quantile, na.rm = TRUE)
                YearlyDayNightOutput[k, 28] = quantile(MonthlyDayNightOutput[,
9][MonthlyDayNightOutput[, 2] == YearlyOutput[i, 2] & MonthlyDayNightOutput[, 4]
== q], 1 - Quantile, na.rm = TRUE)
           k = k + 1
           q = 1
                YearlyDayNightOutput[k, 1] = as.numeric(paste(Site[ColNumber],
".", SegNumber, sep = ""))
                YearlyDayNightOutput[k, 2] = YearlyOutput[i, 2]
                YearlyDayNightOutput[k, 3] = q
                YearlyDayNightOutput[k, 4] = length(TTS$Temperature[TTS$Year ==
YearlyOutput[i, 2] & TTS$DayOrNight == q])
                YearlyDayNightOutput[k, 5] = mean(TTS$Temperature[TTS$Year ==
YearlyOutput[i, 2] & TTS$DayOrNight == q])
                YearlyDayNightOutput[k, 6] = max(TTS$Temperature[TTS$Year ==
YearlyOutput[i, 2] & TTS$DayOrNight == q])
                YearlyDayNightOutput[k, 7] = min(TTS$Temperature[TTS$Year ==
YearlyOutput[i, 2] & TTS$DayOrNight == q])
                YearlyDayNightOutput[k, 8] = YearlyDayNightOutput[k, 6] -
YearlyDayNightOutput[k, 7]
                YearlyDayNightOutput[k, 9] = sd(TTS$Temperature[TTS$Year ==
YearlyOutput[i, 2] & TTS$DayOrNight == q])
                YearlyDayNightOutput[k, 10] = YearlyDayNightOutput[k, 9] /
sqrt(YearlyDayNightOutput[k, 4])
                YearlyDayNightOutput[k, 11] = YearlyDayNightOutput[k, 9] /
YearlyDayNightOutput[k, 5]
                YearlyDayNightOutput[k, 12] = max(TTS$RateOfChange[TTS$Year ==
YearlyOutput[i, 2] & TTS$DayOrNight == q])
                YearlyDayNightOutput[k, 13] = min(TTS$RateOfChange[TTS$Year ==
YearlyOutput[i, 2] & TTS$DayOrNight == q])
                YearlyDayNightOutput[k, 14] = mean(DailyDayNightOutput[,
10][DailyDayNightOutput[, 2] == YearlyOutput[i, 2] & DailyDayNightOutput[, 5] ==
q], na.rm = TRUE)
                YearlyDayNightOutput[k, 15] = max(DailyDayNightOutput[,
10][DailyDayNightOutput[, 2] == YearlyOutput[i, 2] & DailyDayNightOutput[, 5] ==
q], na.rm = TRUE)
                YearlyDayNightOutput[k, 16] = min(DailyDayNightOutput[,
10][DailyDayNightOutput[, 2] == YearlyOutput[i, 2] & DailyDayNightOutput[, 5] ==
q], na.rm = TRUE)
                YearlyDayNightOutput[k, 17] = mean(MonthlyDayNightOutput[,
9][MonthlyDayNightOutput[, 2] == YearlyOutput[i, 2] & MonthlyDayNightOutput[, 4]
== q], na.rm = TRUE)
                YearlyDayNightOutput[k, 18] = max(MonthlyDayNightOutput[,
9][MonthlyDayNightOutput[, 2] == YearlyOutput[i, 2] & MonthlyDayNightOutput[, 4]
== q], na.rm = TRUE)
                YearlyDayNightOutput[k, 19] = min(MonthlyDayNightOutput[,
9][MonthlyDayNightOutput[, 2] == YearlyOutput[i, 2] & MonthlyDayNightOutput[, 4]
== q], na.rm = TRUE)
                YearlyDayNightOutput[k, 20] = YearlyDayNightOutput[k, 14] /
YearlyDayNightOutput[k, 8]
```

```
                YearlyDayNightOutput[k, 21] = quantile(TTS$Temperature[TTS$Year
== YearlyOutput[i, 2] & TTS$DayOrNight == q], Quantile, na.rm =TRUE)
                YearlyDayNightOutput[k, 22] = quantile(TTS$Temperature[TTS$Year
== YearlyOutput[i, 2] & TTS$DayOrNight == q], 1 - Quantile, na.rm =TRUE)
                YearlyDayNightOutput[k, 23] = quantile(TTS$RateOfChange[TTS$Year
== YearlyOutput[i, 2] & TTS$DayOrNight == q], Quantile, na.rm =TRUE)
                YearlyDayNightOutput[k, 24] = quantile(TTS$RateOfChange[TTS$Year
== YearlyOutput[i, 2] & TTS$DayOrNight == q], 1 - Quantile, na.rm =TRUE)
                YearlyDayNightOutput[k, 25] = quantile(DailyDayNightOutput[,
10][DailyDayNightOutput[, 2] == YearlyOutput[i, 2] & DailyDayNightOutput[, 5] ==
q], Quantile, na.rm = TRUE)
                YearlyDayNightOutput[k, 26] = quantile(DailyDayNightOutput[,
10][DailyDayNightOutput[, 2] == YearlyOutput[i, 2] & DailyDayNightOutput[, 5] ==
q], 1 - Quantile, na.rm = TRUE)
                YearlyDayNightOutput[k, 27] = quantile(MonthlyDayNightOutput[,
9][MonthlyDayNightOutput[, 2] == YearlyOutput[i, 2] & MonthlyDayNightOutput[, 4]
== q], Quantile, na.rm = TRUE)
                YearlyDayNightOutput[k, 28] = quantile(MonthlyDayNightOutput[,
9][MonthlyDayNightOutput[, 2] == YearlyOutput[i, 2] & MonthlyDayNightOutput[, 4]
== q], 1 - Quantile, na.rm = TRUE)
k = k + 1}


# Repeat calculations for the entire dataset
k = 1
q = 0
        DatasetDayNightOutput[k, 1] = as.numeric(paste(Site[ColNumber], ".",
SegNumber, sep = ""))
        DatasetDayNightOutput[k, 2] = q
        DatasetDayNightOutput[k, 3] = length(TTS$Temperature[TTS$DayOrNight ==
q])
        DatasetDayNightOutput[k, 4] = mean(TTS$Temperature[TTS$DayOrNight == q])
        DatasetDayNightOutput[k, 5] = max(TTS$Temperature[TTS$DayOrNight == q])
        DatasetDayNightOutput[k, 6] = min(TTS$Temperature[TTS$DayOrNight == q])
        DatasetDayNightOutput[k, 7] = DatasetDayNightOutput[k, 5] -
DatasetDayNightOutput[k, 6]
        DatasetDayNightOutput[k, 8] = sd(TTS$Temperature[TTS$DayOrNight == q])
        DatasetDayNightOutput[k, 9] = DatasetDayNightOutput[k, 8] /
sqrt(DatasetDayNightOutput[k, 3])
        DatasetDayNightOutput[k, 10] = DatasetDayNightOutput[k, 8] /
DatasetDayNightOutput[k, 4]
        DatasetDayNightOutput[k, 11] = max(TTS$RateOfChange[TTS$DayOrNight ==
q], na.rm = TRUE)
        DatasetDayNightOutput[k, 12] = min(TTS$RateOfChange[TTS$DayOrNight ==
q], na.rm = TRUE)
        DatasetDayNightOutput[k, 13] = mean(DailyDayNightOutput[,
10][DailyDayNightOutput[, 5] == q], na.rm = TRUE)
        DatasetDayNightOutput[k, 14] = max(DailyDayNightOutput[,
10][DailyDayNightOutput[, 5] == q], na.rm = TRUE)
        DatasetDayNightOutput[k, 15] = min(DailyDayNightOutput[,
10][DailyDayNightOutput[, 5] == q], na.rm = TRUE)
        DatasetDayNightOutput[k, 16] = mean(MonthlyDayNightOutput[,
```

```
9][MonthlyDayNightOutput[,4] == q], na.rm = TRUE)
        DatasetDayNightOutput[k, 17] = max(MonthlyDayNightOutput[,
9][MonthlyDayNightOutput[, 4] == q], na.rm = TRUE)
        DatasetDayNightOutput[k, 18] = min(MonthlyDayNightOutput[,
9][MonthlyDayNightOutput[, 4] == q], na.rm = TRUE)
        DatasetDayNightOutput[k, 19] = DatasetDayNightOutput[k, 13] /
YearlyDayNightOutput[k, 7]
        DatasetDayNightOutput[k, 20] = quantile(TTS$Temperature[TTS$DayOrNight
== q], Quantile, na.rm = TRUE)
        DatasetDayNightOutput[k, 21] = quantile(TTS$Temperature[TTS$DayOrNight
== q], 1 - Quantile, na.rm = TRUE)
        DatasetDayNightOutput[k, 22] = quantile(TTS$RateOfChange[TTS$DayOrNight
== q], Quantile, na.rm = TRUE)
        DatasetDayNightOutput[k, 23] = quantile(TTS$RateOfChange[TTS$DayOrNight
== q], 1 - Quantile, na.rm = TRUE)
        DatasetDayNightOutput[k, 24] = quantile(DailyDayNightOutput[,
10][DailyDayNightOutput[, 5] == q], Quantile, na.rm = TRUE)
        DatasetDayNightOutput[k, 25] = quantile(DailyDayNightOutput[,
10][DailyDayNightOutput[, 5] == q], 1 - Quantile, na.rm = TRUE)
        DatasetDayNightOutput[k, 26] = quantile(MonthlyDayNightOutput[,
9][MonthlyDayNightOutput[, 4] == q], Quantile, na.rm = TRUE)
        DatasetDayNightOutput[k, 27] = quantile(MonthlyDayNightOutput[,
9][MonthlyDayNightOutput[, 4] == q], 1 - Quantile, na.rm = TRUE)
k = k + 1
q = 1
        DatasetDayNightOutput[k, 1] = as.numeric(paste(Site[ColNumber], ".",
SegNumber, sep = ""))
        DatasetDayNightOutput[k, 2] = q
        DatasetDayNightOutput[k, 3] = length(TTS$Temperature[TTS$DayOrNight ==
q])
        DatasetDayNightOutput[k, 4] = mean(TTS$Temperature[TTS$DayOrNight == q])
        DatasetDayNightOutput[k, 5] = max(TTS$Temperature[TTS$DayOrNight == q])
        DatasetDayNightOutput[k, 6] = min(TTS$Temperature[TTS$DayOrNight == q])
        DatasetDayNightOutput[k, 7] = DatasetDayNightOutput[k, 5] -
DatasetDayNightOutput[k, 6]
        DatasetDayNightOutput[k, 8] = sd(TTS$Temperature[TTS$DayOrNight == q])
        DatasetDayNightOutput[k, 9] = DatasetDayNightOutput[k, 8] /
sqrt(DatasetDayNightOutput[k, 3])
        DatasetDayNightOutput[k, 10] = DatasetDayNightOutput[k, 8] /
DatasetDayNightOutput[k, 4]
        DatasetDayNightOutput[k, 11] = max(TTS$RateOfChange[TTS$DayOrNight ==
q])
        DatasetDayNightOutput[k, 12] = min(TTS$RateOfChange[TTS$DayOrNight ==
q])
        DatasetDayNightOutput[k, 13] = mean(DailyDayNightOutput[,
10][DailyDayNightOutput[, 5] == q], na.rm = TRUE)
        DatasetDayNightOutput[k, 14] = max(DailyDayNightOutput[,
10][DailyDayNightOutput[, 5] == q], na.rm = TRUE)
        DatasetDayNightOutput[k, 15] = min(DailyDayNightOutput[,
10][DailyDayNightOutput[, 5] == q], na.rm = TRUE)
        DatasetDayNightOutput[k, 16] = mean(MonthlyDayNightOutput[,
9][MonthlyDayNightOutput[, 4] == q], na.rm = TRUE)
```

```
        DatasetDayNightOutput[k, 17] = max(MonthlyDayNightOutput[,
9][MonthlyDayNightOutput[, 4] == q], na.rm = TRUE)
        DatasetDayNightOutput[k, 18] = min(MonthlyDayNightOutput[,
9][MonthlyDayNightOutput[, 4] == q], na.rm = TRUE)
        DatasetDayNightOutput[k, 19] = DatasetDayNightOutput[k, 13] /
YearlyDayNightOutput[k, 7]
        DatasetDayNightOutput[k, 20] = quantile(TTS$Temperature[TTS$DayOrNight
== q], Quantile, na.rm = TRUE)
        DatasetDayNightOutput[k, 21] = quantile(TTS$Temperature[TTS$DayOrNight
== q], 1 - Quantile, na.rm = TRUE)
        DatasetDayNightOutput[k, 22] = quantile(TTS$RateOfChange[TTS$DayOrNight
== q], Quantile, na.rm = TRUE)
        DatasetDayNightOutput[k, 23] = quantile(TTS$RateOfChange[TTS$DayOrNight
== q], 1 - Quantile, na.rm = TRUE)
        DatasetDayNightOutput[k, 24] = quantile(DailyDayNightOutput[,
10][DailyDayNightOutput[, 5] == q], Quantile, na.rm = TRUE)
        DatasetDayNightOutput[k, 25] = quantile(DailyDayNightOutput[,
10][DailyDayNightOutput[, 5] == q], 1 - Quantile, na.rm = TRUE)
        DatasetDayNightOutput[k, 26] = quantile(MonthlyDayNightOutput[,
9][MonthlyDayNightOutput[, 4] == q], Quantile, na.rm = TRUE)
        DatasetDayNightOutput[k, 27] = quantile(MonthlyDayNightOutput[,
9][MonthlyDayNightOutput[, 4] == q], 1 - Quantile, na.rm = TRUE)

# Remove any rows with missing values
DailyDayNightOutput <- DailyDayNightOutput[-c(which(is.na(DailyDayNightOutput[,
10]) == TRUE)), ]
MonthlyDayNightOutput <-
MonthlyDayNightOutput[-c(which(is.na(MonthlyDayNightOutput[, 1]) == TRUE)), ]

# Creat the output files
if (file.exists(paste(OutputFileName, "_DataDayNightSummary.csv", sep = "")))
{write.table(DatasetDayNightOutput, file = paste(OutputFileName,
"_DataDayNightSummary.csv", sep = ""), append = TRUE, sep = ",", dec = ".",
quote = FALSE, na = "NA", col.names = FALSE, row.names = FALSE)} else
{write.table(DatasetDayNightOutput, file = paste(OutputFileName,
"_DataDayNightSummary.csv", sep = ""), append = FALSE, sep = ",", dec = ".",
quote = FALSE, na = "NA", col.names = TRUE, row.names = FALSE)}
if (file.exists(paste(OutputFileName, "_AnnualDayNightSummary.csv", sep = "")))
{write.table(YearlyDayNightOutput, file = paste(OutputFileName,
"_AnnualDayNightSummary.csv", sep = ""), append = TRUE, sep = ",", dec = ".",
quote = FALSE, na = "NA", col.names = FALSE, row.names = FALSE)} else
{write.table(YearlyDayNightOutput, file = paste(OutputFileName,
"_AnnualDayNightSummary.csv", sep = ""), append = FALSE, sep = ",", dec = ".",
quote = FALSE, na = "NA", col.names = TRUE, row.names = FALSE)}
if (file.exists(paste(OutputFileName, "_MonthlyDayNightSummary.csv", sep = "")))
{write.table(MonthlyDayNightOutput[, c(1 : 17, 22 : 27)], file =
paste(OutputFileName, "_MonthlyDayNightSummary.csv", sep = ""), append = TRUE,
sep = ",", dec = ".", quote = FALSE, na = "NA", col.names = FALSE, row.names =
FALSE)} else {write.table(MonthlyDayNightOutput[, c(1 : 17, 22 : 27)], file =
paste(OutputFileName, "_MonthlyDayNightSummary.csv", sep = ""), append = FALSE,
sep = ",", dec = ".", quote = FALSE, na = "NA", col.names = TRUE, row.names =
FALSE)}
```

```
if (file.exists(paste(OutputFileName, "_DailyDayNightSummary.csv", sep = "")))
{write.table(DailyDayNightOutput[, 1 : 15], file = paste(OutputFileName,
"_DailyDayNightSummary.csv", sep = ""), append = TRUE, sep = ",", dec = ".",
quote = FALSE, na = "NA", col.names = FALSE, row.names = FALSE)} else
{write.table(DailyDayNightOutput[, 1 : 15], file = paste(OutputFileName,
"_DailyDayNightSummary.csv", sep = ""), append = FALSE, sep = ",", dec = ".",
quote = FALSE, na = "NA", col.names = TRUE, row.names = FALSE)}
if (file.exists(paste(OutputFileName, "_MonthDayNightSummary.csv", sep = "")))
{write.table(MonthlyDayNightSummary[, c(1 : 19, 21 : 28)], file =
paste(OutputFileName, "_MonthDayNightSummary.csv", sep = ""), append = TRUE, sep
= ",", dec = ".", quote = FALSE, na = "NA", col.names = FALSE, row.names =
FALSE)} else {write.table(MonthlyDayNightSummary[, c(1 : 19, 21 : 28)], file =
paste(OutputFileName, "_MonthDayNightSummary.csv", sep = ""), append = FALSE,
sep = ",", dec = ".", quote = FALSE, na = "NA", col.names = TRUE, row.names =
FALSE)}

remove(DailyDayNightOutput, MonthlyDayNightOutput, YearlyDayNightOutput,
DatasetDayNightOutput)
}       # ends day/night calculations




##########################################
# 8. Calculate temporal autocorrelation #
##########################################

if (lag.interval == "days") {interval = 60 * 60 * 24}
if (lag.interval == "hours") {interval = 60 * 60}
lags.seq <- seq(from = 0, to = lag.max * interval, by = interval)

# Perform the calculation for the whole dataset, saving the resulting
correlograms and creating an output file containing the autocorrelation values
if (Do.DatasetAutocorrel == "y") {

if (UseAllReadingsForACF == "n") {TempTTS <- subset(TTS, strptime(TTS$DateTime,
format = "%Y-%m-%d %H:%M:%S")$hour %in% HoursToUse & strptime(TTS$DateTime,
format = "%Y-%m-%d %H:%M:%S")$min %in% MinutesToUse)} else {TempTTS <- TTS}
detrended <- lm(TempTTS$Temperature ~ TempTTS$TimeDiff + sin(2 * pi *
TempTTS$TimeDiff/(365.25 * 24)) + cos(2 * pi * TempTTS$TimeDiff / (365.25 *
24)))
TempTTS$Temperature <- TempTTS$Temperature - predict(detrended)
output.matrix.mc.dataset <- matrix(data = NA, nrow = 1, ncol = 2 +
length(lags.seq))
LagColNames <- c("Site", "n", "First n.s. lag")
DatasetAutocorrel.p.values <- as.vector(rep(NA, length(lags.seq)))
names(DatasetAutocorrel.p.values) <- c("Site", paste("AC_lag", 1 : lag.max, sep
= ""))
DatasetAutocorrel.p.values[1] <- as.numeric(paste(Site[ColNumber], ".",
SegNumber, sep = ""))
for (l in 1 : (length(lags.seq) - 1)) {LagColNames <- c(LagColNames, paste("AC
lag",l))}
```

```
colnames(output.matrix.mc.dataset) <- LagColNames
output.matrix.mc.dataset[1, 1] <- as.numeric(paste(Site[ColNumber], ".",
SegNumber, sep = ""))
output.matrix.mc.dataset[1, 2] <- length(TempTTS[, 1])
if (length(TempTTS[, 1]) < 15 | var(TempTTS$Temperature) == 0) {next}
first.ns.lag <- 0
for (lag in 1 : (length(lags.seq) - 1)) {
        correlogram.mc.gr <- NULL
        correlogram.mc.ls <- NULL
        try(correlogram.mc.gr <- moran.mc(TempTTS$Temperature,
nb2listw(dnearneigh(cbind(as.numeric(TempTTS$DateTime), rep(1,
length(TempTTS$DateTime))), lags.seq[lag], (lags.seq[lag + 1])), style = "B",
zero.policy = TRUE), zero.policy = TRUE, nsim = 199, alternative = "greater"))
        try(correlogram.mc.ls <- moran.mc(TempTTS$Temperature,
nb2listw(dnearneigh(cbind(as.numeric(TempTTS$DateTime), rep(1,
length(TempTTS$DateTime))), lags.seq[lag], (lags.seq[lag + 1])), style = "B",
zero.policy = TRUE), zero.policy = TRUE, nsim = 199, alternative = "less"))
        if (is.null(correlogram.mc.gr) | is.null(correlogram.mc.ls)) {next}
        output.matrix.mc.dataset[1, 3 + lag] <- correlogram.mc.gr$statistic
        if (correlogram.mc.gr$p.value >= 0.05 & correlogram.mc.ls$p.value >=
0.05) {DatasetAutocorrel.p.values[lag + 1] <- "n.s."} else
{DatasetAutocorrel.p.values[lag + 1] <- min(correlogram.mc.ls$p.value,
correlogram.mc.gr$p.value)}
        if (first.ns.lag < 1) {if (correlogram.mc.gr$p.value >= 0.05 &
correlogram.mc.ls$p.value >= 0.05) {first.ns.lag = lag}}}
output.matrix.mc.dataset[1, 3] <- first.ns.lag
if (Plot.DatasetAutocorrel == "y") {
                tiff(paste(OutputFileName, "_Site_", Site[ColNumber], ".",
SegNumber, "_Dataset_Autocorrel.tif", sep = ""), width = 180, height = 240,
units = "mm", res = 72, compression = "none")
                plot(1 : max(lags.seq / interval), output.matrix.mc.dataset[1, 4
: (length(output.matrix.mc.dataset[1, ]))], ylim = c(-1, 1), xlab = paste("Time
lag (", lag.interval, ")", sep = ""), ylab = "Moran's I", type = "b", bty = "n")
                title(paste("Site: ", Site[ColNumber], ".", SegNumber, sep =
""), cex.main = 0.9)
                lag.axis <- 1 : max(lags.seq / interval)
                points(lag.axis[which(DatasetAutocorrel.p.values[2 : (lag.max +
1)] != "n.s.")], output.matrix.mc.dataset[1, 4 : (2 +
length(lags.seq))][which(DatasetAutocorrel.p.values[2 : (lag.max + 1)] !=
"n.s.")], pch = 16, col = "red")
                abline(h = 0)
                dev.off()}
if (file.exists(paste(OutputFileName, "_DatasetAutocorrel.csv", sep = "")))
{write.table(output.matrix.mc.dataset, file = paste(OutputFileName,
"_DatasetAutocorrel.csv", sep = ""), append = TRUE, sep = ",", dec = ".", quote
= FALSE, na = "NA", col.names = FALSE, row.names = FALSE)} else
{write.table(output.matrix.mc.dataset, file = paste(OutputFileName,
"_DatasetAutocorrel.csv", sep = ""), append = FALSE, sep = ",", dec = ".", quote
= FALSE, na = "NA", col.names = TRUE, row.names = FALSE)}
if (file.exists(paste(OutputFileName, "_DatasetAutocorrel_p_values.csv", sep =
""))) {write.table(DatasetAutocorrel.p.values, file = paste(OutputFileName,
"_DatasetAutocorrel_p_values.csv", sep = ""), append = TRUE, sep = ",", dec =
```

```
".", quote = FALSE, na = "NA", col.names = FALSE, row.names = TRUE)} else
{write.table(DatasetAutocorrel.p.values, file = paste(OutputFileName,
"_DatasetAutocorrel_p_values.csv", sep = ""), append = FALSE, sep = ",", dec =
".", quote = FALSE, na = "NA", col.names = TRUE, row.names = TRUE)}}


# Repeat the calculation per year, saving the resulting correlograms and
creating an output file containing the autocorrelation values
if (Do.YearlyAutocorrel == "y") {
output.matrix.mc.year <- matrix(data = NA, nrow = 1 + max(TTS$Year) -
min(TTS$Year), ncol = 3 + length(lags.seq))
LagColNames <- c("Site", "Year", "n", "First n.s. lag")
for (l in 1 : (length(lags.seq) - 1)) {LagColNames <- c(LagColNames, paste("AC
lag", l))}
colnames(output.matrix.mc.year) <- LagColNames
YearlyAutocorrel.p.values <- matrix(data = NA, nrow = 1 + max(TTS$Year) -
min(TTS$Year), ncol = length(lags.seq) + 1)
colnames(YearlyAutocorrel.p.values) <- c("Site", "Year", paste("AC_lag", 1 :
lag.max, sep = ""))
YearlyAutocorrel.p.values[, 1] <- rep(as.numeric(paste(Site[ColNumber], ".",
SegNumber, sep = "")), length(YearlyAutocorrel.p.values[, 1]))
for (i in 1 : length(YearlyOutput[, 1])) {
        if (YearlyOutput[i, 3] == 0) {next}
        TempTTS <- subset(TTS, TTS$Year == YearlyOutput[i, 2])
        if (UseAllReadingsForACF == "n") {TempTTS <- subset(TempTTS,
strptime(TempTTS$DateTime, format = "%Y-%m-%d %H:%M:%S")$hour %in% HoursToUse &
strptime(TempTTS$DateTime, format = "%Y-%m-%d %H:%M:%S")$min %in% MinutesToUse)}
        detrended <- lm(TempTTS$Temperature ~ TempTTS$TimeDiff + sin(2 * pi *
TempTTS$TimeDiff / (365.25 * 24)) + cos(2 * pi * TempTTS$TimeDiff / (365.25 *
24)))
        TempTTS$Temperature <- TempTTS$Temperature - predict(detrended)
        output.matrix.mc.year[i, 1] <- as.numeric(paste(Site[ColNumber], ".",
SegNumber, sep = ""))
        output.matrix.mc.year[i, 2] <- YearlyOutput[i, 2]
        output.matrix.mc.year[i, 3] <- length(TempTTS[, 1])
        if (length(TempTTS[, 1]) < 15 | var(TempTTS$Temperature) == 0) {next}
        first.ns.lag <- 0
        for (lag in 1 : (length(lags.seq) - 1)) {
                correlogram.mc.gr <- NULL
                correlogram.mc.ls <- NULL
                try(correlogram.mc.gr <- moran.mc(TempTTS$Temperature,
nb2listw(dnearneigh(cbind(as.numeric(TempTTS$DateTime), rep(1,
length(TempTTS$DateTime))), lags.seq[lag], (lags.seq[lag + 1])), style = "B",
zero.policy = TRUE), zero.policy = TRUE, nsim = 199, alternative = "greater"))
                try(correlogram.mc.ls <- moran.mc(TempTTS$Temperature,
nb2listw(dnearneigh(cbind(as.numeric(TempTTS$DateTime), rep(1,
length(TempTTS$DateTime))), lags.seq[lag], (lags.seq[lag + 1])), style = "B",
zero.policy = TRUE), zero.policy = TRUE, nsim = 199, alternative = "less"))
                if (is.null(correlogram.mc.gr) | is.null(correlogram.mc.ls))
{next}
                output.matrix.mc.year[i, 4 + lag] <- correlogram.mc.gr$statistic
                YearlyAutocorrel.p.values[i, 2] <- YearlyOutput[i, 2]
```

```
                if (correlogram.mc.gr$p.value >= 0.05 &
correlogram.mc.ls$p.value >= 0.05) {YearlyAutocorrel.p.values[i, (lag + 1)] <-
"n.s."} else {YearlyAutocorrel.p.values[i, (lag + 1)] <-
min(correlogram.mc.ls$p.value, correlogram.mc.gr$p.value)}
                if (first.ns.lag < 1) {if (correlogram.mc.gr$p.value >= 0.05 &
correlogram.mc.ls$p.value >= 0.05) {first.ns.lag = lag}}}
        output.matrix.mc.year[i, 4] <- first.ns.lag
        if (Plot.YearlyAutocorrel == "y") {
                tiff(paste(OutputFileName, "_Site_", Site[ColNumber], ".",
SegNumber, "_Year_", YearlyOutput[i, 2], "_Autocorrel.tif", sep = ""), width =
180, height = 240, units = "mm", res = 72, compression = "none")
                plot(1 : max(lags.seq / interval), output.matrix.mc.year[i, 5 :
(length(output.matrix.mc.year[i, ]))], ylim = c(-1, 1), xlab = paste("Time lag
(", lag.interval, ")", sep = ""), ylab = "Moran's I", type = "b", bty = "n")
                lag.axis <- 1 : max(lags.seq / interval)
                points(lag.axis[which(YearlyAutocorrel.p.values[i, 2 :
length(YearlyAutocorrel.p.values[1, ])] != "n.s.")], output.matrix.mc.year[i, 5
: (3 + length(lags.seq))][which(YearlyAutocorrel.p.values[i, 2 :
length(YearlyAutocorrel.p.values[1, ])] != "n.s.")], pch = 16, col = "red")
                title(paste("Site: " ,Site[ColNumber], ".", SegNumber, " Year:
", YearlyOutput[i, 2], sep = ""), cex.main = 0.9)
                abline(h = 0)
                dev.off()}}
if (file.exists(paste(OutputFileName, "_YearlyAutocorrel.csv", sep = "")))
{write.table(output.matrix.mc.year, file = paste(OutputFileName,
"_YearlyAutocorrel.csv", sep = ""), append = TRUE, sep = ",", dec = ".", quote =
FALSE, na = "NA", col.names = FALSE, row.names = FALSE)} else
{write.table(output.matrix.mc.year, file = paste(OutputFileName,
"_YearlyAutocorrel.csv", sep = ""), append = FALSE, sep = ",", dec = ".", quote
= FALSE, na = "NA", col.names = TRUE, row.names = FALSE)}
if (file.exists(paste(OutputFileName, "_YearlyAutocorrel_p_values.csv", sep =
""))) {write.table(YearlyAutocorrel.p.values, file = paste(OutputFileName,
"_YearlyAutocorrel_p_values.csv", sep = ""), append = TRUE, sep = ",", dec =
".", quote = FALSE, na = "NA", col.names = FALSE, row.names = FALSE)} else
{write.table(YearlyAutocorrel.p.values, file = paste(OutputFileName,
"_YearlyAutocorrel_p_values.csv", sep = ""), append = FALSE, sep = ",", dec =
".", quote = FALSE, na = "NA", col.names = TRUE, row.names = FALSE)}}

# Repeat the calculation per month, saving the resulting correlograms and
creating an output file containing the autocorrelation values
if (Do.MonthlyAutocorrel == "y") {
output.matrix.mc.month <- matrix(data = NA, nrow = 12 * (1 + max(TTS$Year) -
min(TTS$Year)), ncol = 4 + length(lags.seq))
LagColNames <- c("Site", "Year", "Month", "n", "First n.s. lag")
for (l in 1 : (length(lags.seq) - 1)) {LagColNames <- c(LagColNames, paste("AC
lag", l))}
colnames(output.matrix.mc.month) <- LagColNames
MonthlyAutocorrel.p.values <- matrix(data = NA, nrow =
length(unique(paste(TTS$Year, TTS$Month))), ncol = length(lags.seq) + 2)
colnames(MonthlyAutocorrel.p.values) <- c("Site", "Year", "Month",
paste("AC_lag", 1 : lag.max, sep = ""))
```

```
for (i in 1 : length(MonthlyOutput[, 1])) {
	if (MonthlyOutput[i, 4] == 0) {next}
	if (UseAllReadingsForACF == "n") {
	TempTTS <- subset(TTS, strptime(TTS$DateTime, format = "%Y-%m-%d
%H:%M:%S")$hour %in% HoursToUse & strptime(TTS$DateTime, format = "%Y-%m-%d
%H:%M:%S")$min %in% MinutesToUse)} else {TempTTS <- TTS}
	TempTTS <- subset(TempTTS,TempTTS$Year == MonthlyOutput[i, 2] &
TempTTS$Month == MonthlyOutput[i, 3])
	detrended <- lm(TempTTS$Temperature ~ TempTTS$TimeDiff)
	TempTTS$Temperature <- TempTTS$Temperature - predict(detrended)
	output.matrix.mc.month[i, 1] <- as.numeric(paste(Site[ColNumber], ".",
SegNumber, sep = ""))
	output.matrix.mc.month[i, 2] <- MonthlyOutput[i, 2]
	output.matrix.mc.month[i, 3] <- MonthlyOutput[i, 3]
	output.matrix.mc.month[i, 4] <- length(TempTTS[, 1])
	if (length(TempTTS[, 1]) < 15 | var(TempTTS$Temperature) == 0) {next}
	first.ns.lag <- 0
	for (lag in 1:(length(lags.seq) - 1)) {
		correlogram.mc.gr <- NULL
		correlogram.mc.ls <- NULL
		try(correlogram.mc.gr <- moran.mc(TempTTS$Temperature,
nb2listw(dnearneigh(cbind(as.numeric(TempTTS$DateTime), rep(1,
length(TempTTS$DateTime))), lags.seq[lag], (lags.seq[lag + 1])), style = "B",
zero.policy = TRUE), zero.policy = TRUE, nsim = 99, alternative = "greater"))
		try(correlogram.mc.ls <- moran.mc(TempTTS$Temperature,
nb2listw(dnearneigh(cbind(as.numeric(TempTTS$DateTime), rep(1,
length(TempTTS$DateTime))), lags.seq[lag], (lags.seq[lag + 1])), style = "B",
zero.policy = TRUE), zero.policy = TRUE, nsim = 99, alternative = "less"))
		if (is.null(correlogram.mc.gr) | is.null(correlogram.mc.ls))
{next}
		output.matrix.mc.month[i, 5 + lag] <-
correlogram.mc.gr$statistic
		MonthlyAutocorrel.p.values[i, 1] <-
as.numeric(paste(Site[ColNumber], ".", SegNumber, sep = ""))
		MonthlyAutocorrel.p.values[i, 2] <- MonthlyOutput[i, 2]
		MonthlyAutocorrel.p.values[i, 3] <- MonthlyOutput[i, 3]
    if (correlogram.mc.gr$p.value >= 0.05 & correlogram.mc.ls$p.value >= 0.05)
{MonthlyAutocorrel.p.values[i, (lag + 3)] <- "n.s."} else
{MonthlyAutocorrel.p.values[i, (lag + 3)] <- min(correlogram.mc.ls$p.value,
correlogram.mc.gr$p.value)}
		if (first.ns.lag < 1) {if (correlogram.mc.gr$p.value >= 0.05 &
correlogram.mc.ls$p.value >= 0.05) {first.ns.lag <- lag}}}
	output.matrix.mc.month[i, 5] <- first.ns.lag

	if (Plot.MonthlyAutocorrel == "y") {
		tiff(paste(OutputFileName, "_Site_", Site[ColNumber], ".",
SegNumber, "_Year_", MonthlyOutput[i, 2], "_Month_", MonthlyOutput[i, 3],
"_Autocorrel.tif", sep = ""), width = 180, height = 240, units = "mm", res = 72,
compression = "none")
		plot(1 : max(lags.seq / interval), output.matrix.mc.month[i, 6 :
(length(output.matrix.mc.month[i, ]))], ylim = c(-1, 1), xlab = paste("Time lag
(",lag.interval, ")", sep = ""), ylab = "Moran's I", type = "b", bty = "n")
```

```
                lag.axis <- 1 : max(lags.seq / interval)
                points(lag.axis[which(MonthlyAutocorrel.p.values[i, 4 :
length(MonthlyAutocorrel.p.values[1, ])] != "n.s.")], output.matrix.mc.month[i,
6 : (4 + length(lags.seq))][which(MonthlyAutocorrel.p.values[i, 4 :
length(MonthlyAutocorrel.p.values[1, ])] != "n.s.")], pch = 16, col = "red")
                title(paste("Site: " ,Site[ColNumber], ".", SegNumber, " Month:
", MonthlyOutput[i, 3], "/", MonthlyOutput[i, 2], sep = ""), cex.main = 0.9)
                abline(h = 0)
                dev.off()}}
# remove rows with no data
output.matrix.mc.month <-
output.matrix.mc.month[-c(which(is.na(output.matrix.mc.month[, 1]) == TRUE)), ]
MonthlyAutocorrel.p.values <-
MonthlyAutocorrel.p.values[-c(which(is.na(MonthlyAutocorrel.p.values[, 1]) ==
TRUE)), ]
# save output files
if (file.exists(paste(OutputFileName, "_MonthlyAutocorrel.csv", sep = "")))
{write.table(output.matrix.mc.month, file = paste(OutputFileName,
"_MonthlyAutocorrel.csv", sep = ""), append = TRUE, sep = ",", dec=".", quote =
FALSE, na = "NA", col.names = FALSE, row.names = FALSE)} else
{write.table(output.matrix.mc.month, file = paste(OutputFileName,
"_MonthlyAutocorrel.csv", sep = ""), append = FALSE, sep = ",", dec = ".", quote
= FALSE, na = "NA", col.names = TRUE, row.names = FALSE)}
if (file.exists(paste(OutputFileName, "_MonthlyAutocorrel_p_values.csv", sep =
""))) {write.table(MonthlyAutocorrel.p.values, file = paste(OutputFileName,
"_MonthlyAutocorrel_p_values.csv", sep = ""), append = TRUE, sep = ",", dec =
".", quote = FALSE, col.names = FALSE, row.names = FALSE)} else
{write.table(MonthlyAutocorrel.p.values, file = paste(OutputFileName,
"_MonthlyAutocorrel_p_values.csv", sep = ""), append = FALSE, sep = ",", dec =
".", quote = FALSE, col.names = TRUE, row.names = FALSE)}}


############################################
# 9. Calculate duration between thresholds #
############################################

# Check if the users has asked for these calculations
if (Do.Thresholds == "y") {

# Choose time series (i.e. longest duration and/or interpolation) to use for
calculations, based on the users choice in section 2b).
if (Thresholds.Use.TTS == "y") {Threshold.TTS <- TTS}
if (Thresholds.Use.LongestDuration == "y") {Threshold.TTS <- LongestDuration}
if (Thresholds.Use.Interpolated.TTS == "y") {Threshold.TTS <- Interpolated.TTS}
if (Thresholds.Use.Interpolated.LongestDuration == "y") {Threshold.TTS <-
Interpolated.LongestDuration.TTS}
if (Thresholds.Use.Interpolated.Sinus.TTS == "y") {Threshold.TTS <-
Interpolated.Sinus.TTS}

# Define column names and create an output matrix
Btwn.threshold <- matrix(ncol = 2, data = Thresholds, byrow = TRUE)
dimnames(Btwn.threshold) = list(Threshold.names,c("Min", "Max"))
```

```
Btwn.parameters <- c("OccurrenceBetween", "HoursBetween",
"MaxDurationHoursBetween", "HourDegreesBetween")
BtwnParameterNames <- c("n", "Response1", "Response2", "Response3")
for (p in 1 : (length(Btwn.threshold) / 2)) {
        for (q in 1 : length(Btwn.parameters)) {
                BtwnParameterNames <-
c(BtwnParameterNames,paste(Btwn.parameters[q], Btwn.threshold[p, 1], "and",
Btwn.threshold[p, 2], sep = ""))}}
DatasetBtwnOutput <- matrix(ncol = length(BtwnParameterNames) + 1, nrow = 1)
YearlyBtwnOutput <- matrix(ncol = length(BtwnParameterNames) + 2, nrow =
length(unique(Threshold.TTS$Year)))
MonthlyBtwnOutput <- matrix(ncol = length(BtwnParameterNames) + 3, nrow =
length(unique(paste(Threshold.TTS$Year, Threshold.TTS$Month))))
colnames(DatasetBtwnOutput) <- c("Site", BtwnParameterNames)
colnames(YearlyBtwnOutput) <- c("Site", "Year", BtwnParameterNames)
colnames(MonthlyBtwnOutput) <- c("Site", "Year", "Month", BtwnParameterNames)
remove(BtwnParameterNames)


# a = counts occurrences above threshold
# b = total duration above threshold
# f = maximum duration above threshold
# d is a counter
# e = sum of values within threshold (i.e. degree days or frost degree days if
subzero)
# g = sum of values of response function (multiplied by hourly interval)


# Calculate the threshold related paramters for the whole dataset
if (Do.DatasetBtwnOutput == "y") {
        i = 1
        k = 1
        j = length(Threshold.TTS$Temperature)
        DatasetBtwnOutput[k, 1] = as.numeric(paste(Site[ColNumber], ".",
SegNumber, sep = ""))
        DatasetBtwnOutput[k, 2] = j
        # loop through all records and sum up responses
        g1 = 0; g2 = 0; g3 = 0
        for (l in 0 : (j - 1)) {
                if (Threshold.TTS$TimeChange[l + i] > ShortGap)
{Threshold.TTS$TimeChange[l + i] <- ShortGap}
                g1 = g1 + (TemperatureResponse1(Threshold.TTS$Temperature[l +
i]) * Threshold.TTS$TimeChange[l + i])
                g2 = g2 + (TemperatureResponse2(Threshold.TTS$Temperature[l +
i]) * Threshold.TTS$TimeChange[l + i])
                g3 = g3 + (TemperatureResponse3(Threshold.TTS$Temperature[l +
i]) * Threshold.TTS$TimeChange[l + i])}
        DatasetBtwnOutput[k, 3] = g1
        DatasetBtwnOutput[k, 4] = g2
        DatasetBtwnOutput[k, 5] = g3
        # loop through all records, summing threshold values
```

```
        for (d in 1 : (length(Btwn.threshold) / 2)) {
                a = 0; b = 0; e = 0; f = 0
                flist <- as.vector(0)
        for (l in 0 : (j - 1)) {
                if (l == 0) {
                        if (difftime(Threshold.TTS$DateTime[l + i +
1],Threshold.TTS$DateTime[l + i], units = "hours") < ShortGap)
{Threshold.TTS$TimeDiff[l + i] <- difftime(Threshold.TTS$DateTime[l + i + 1],
Threshold.TTS$DateTime[l + i], units = "hours")} else {Threshold.TTS$TimeDiff[l
+ i] <- ShortGap}
                        if (Threshold.TTS$Temperature[l + i] > Btwn.threshold[d,
1] & Threshold.TTS$Temperature[l + i] < Btwn.threshold[d, 2]) {a = a + 1}
                        if (Threshold.TTS$Temperature[l + i] > Btwn.threshold[d,
1] & Threshold.TTS$Temperature[l + i] < Btwn.threshold[d, 2]) {b = b +
as.numeric(Threshold.TTS$TimeDiff[l + i])}
                        if (Threshold.TTS$Temperature[l + i] > Btwn.threshold[d,
1] & Threshold.TTS$Temperature[l + i] < Btwn.threshold[d, 2]) {f = f +
(as.numeric(Threshold.TTS$TimeDiff[l + i]))} else {flist = c(flist, f); f = 0}
                        if (Threshold.TTS$Temperature[l + i] > Btwn.threshold[d,
1] & Threshold.TTS$Temperature[l + i] < Btwn.threshold[d, 2]) {if
(Btwn.threshold[d, 1] != -Inf) {e = e + ((Threshold.TTS$Temperature[l + i] -
Btwn.threshold[d, 1]) * Threshold.TTS$TimeDiff[l + i])} else {e = e +
(Btwn.threshold[d, 2] - Threshold.TTS$Temperature[l + i]) *
Threshold.TTS$TimeDiff[l + i]}}}
                if (l == (j - 1)) {
                        if (difftime(Threshold.TTS$DateTime[l +
i],Threshold.TTS$DateTime[l + i - 1], units = "hours") < ShortGap)
{Threshold.TTS$TimeDiff[l + i] <- difftime(Threshold.TTS$DateTime[l + i],
Threshold.TTS$DateTime[l + i - 1], units = "hours")} else
{Threshold.TTS$TimeDiff[l + i] <- ShortGap}
                        if (Threshold.TTS$Temperature[l + i] > Btwn.threshold[d,
1] & Threshold.TTS$Temperature[l + i] < Btwn.threshold[d, 2] &
(Threshold.TTS$Temperature[l + i - 1] <= Btwn.threshold[d, 1] |
Threshold.TTS$Temperature[l + i - 1] >= Btwn.threshold[d, 2])) {a = a + 1}
                        if (Threshold.TTS$Temperature[l + i] > Btwn.threshold[d,
1] & Threshold.TTS$Temperature[l + i] < Btwn.threshold[d, 2]) {b = b +
as.numeric(Threshold.TTS$TimeDiff[l + i])}
                        if (Threshold.TTS$Temperature[l + i] > Btwn.threshold[d,
1] & Threshold.TTS$Temperature[l + i] < Btwn.threshold[d, 2]) {f = f +
(as.numeric(Threshold.TTS$TimeDiff[l + i])); flist = c(flist, f)} else {flist =
c(flist, f); f = 0}
                        if (Threshold.TTS$Temperature[l + i] > Btwn.threshold[d,
1] & Threshold.TTS$Temperature[l + i] < Btwn.threshold[d, 2]) {if
(Btwn.threshold[d, 1] != -Inf) {e = e + ((Threshold.TTS$Temperature[l + i] -
Btwn.threshold[d, 1]) * Threshold.TTS$TimeDiff[l + i])} else {e = e +
(Btwn.threshold[d, 2] - Threshold.TTS$Temperature[l + i]) *
Threshold.TTS$TimeDiff[l + i]}}}
                if (l != 0 & l != (j - 1)) {
                        if (difftime(Threshold.TTS$DateTime[l +
i],Threshold.TTS$DateTime[l + i - 1], units = "hours") < ShortGap)
{Threshold.TTS$TimeDiff[l + i] <- difftime(Threshold.TTS$DateTime[l +
i],Threshold.TTS$DateTime[l + i - 1], units = "hours")} else
```

```
{Threshold.TTS$TimeDiff[l + i] <- ShortGap}
                        if (Threshold.TTS$Temperature[l + i] > Btwn.threshold[d,
1] & Threshold.TTS$Temperature[l + i] < Btwn.threshold[d, 2] &
(Threshold.TTS$Temperature[l + i - 1] <= Btwn.threshold[d, 1] |
Threshold.TTS$Temperature[l + i - 1] >= Btwn.threshold[d, 2])) {a = a + 1}
                        if (Threshold.TTS$Temperature[l + i] > Btwn.threshold[d,
1] & Threshold.TTS$Temperature[l + i] < Btwn.threshold[d, 2]) {b = b +
as.numeric(Threshold.TTS$TimeDiff[l + i])}
                        if (Threshold.TTS$Temperature[l + i] > Btwn.threshold[d,
1] & Threshold.TTS$Temperature[l + i] < Btwn.threshold[d, 2]) {f = f +
(as.numeric(Threshold.TTS$TimeDiff[l + i]))} else {flist = c(flist, f); f = 0}
                        if (Threshold.TTS$Temperature[l + i] > Btwn.threshold[d,
1] & Threshold.TTS$Temperature[l + i] < Btwn.threshold[d, 2]) {if
(Btwn.threshold[d, 1] != -Inf) {e = e + ((Threshold.TTS$Temperature[l + i] -
Btwn.threshold[d, 1]) * Threshold.TTS$TimeDiff[l + i])} else {e = e +
(Btwn.threshold[d, 2] - Threshold.TTS$Temperature[l + i]) *
Threshold.TTS$TimeDiff[l + i]}}}}
        DatasetBtwnOutput[k,6 + (4 * (d - 1))] = a
        DatasetBtwnOutput[k,7 + (4 * (d - 1))] = b
        DatasetBtwnOutput[k,8 + (4 * (d - 1))] = max(flist)
        DatasetBtwnOutput[k,9 + (4 * (d - 1))] = e
}
if (file.exists(paste(OutputFileName, "_DataThresholds.csv", sep = "")))
{write.table(DatasetBtwnOutput, file = paste(OutputFileName,
"_DataThresholds.csv", sep = ""), append = TRUE, sep = ",", dec = ".", quote =
FALSE, col.names = FALSE, row.names = FALSE)} else
{write.table(DatasetBtwnOutput, file = paste(OutputFileName,
"_DataThresholds.csv", sep = ""), append = FALSE, sep = ",", dec = ".", quote =
FALSE, col.names = TRUE, row.names = FALSE)}
remove(DatasetBtwnOutput, a, b, e, f, g1, g2, g3)}


# Calculate the threshold related paramters per year
if (Do.YearlyBtwnOutput == "y") {
i = 1; j = 0; k = 1
while (i + j <= length(Threshold.TTS$Temperature) ) {
        if (Threshold.TTS$Year[i] == Threshold.TTS$Year[i + j]) {j = j + 1} else
{
        YearlyBtwnOutput[k, 1] = as.numeric(paste(Site[ColNumber], ".",
SegNumber, sep = ""))
        YearlyBtwnOutput[k, 2] = Threshold.TTS$Year[i]
        YearlyBtwnOutput[k, 3] =
length(Threshold.TTS$Temperature[Threshold.TTS$Year == Threshold.TTS$Year[i]])

        # loop through all records and sum up responses
        g1 = 0; g2 = 0; g3 = 0
        for (l in 0 : (j - 1)) {
                if (Threshold.TTS$TimeChange[l + i] > ShortGap)
{Threshold.TTS$TimeChange[l + i] <- ShortGap}
                g1 = g1 + (TemperatureResponse1(Threshold.TTS$Temperature[l +
i]) * Threshold.TTS$TimeDiff[l + i])
                g2 = g2 + (TemperatureResponse2(Threshold.TTS$Temperature[l +
```

```
i]) * Threshold.TTS$TimeDiff[l + i])
                g3 = g3 + (TemperatureResponse3(Threshold.TTS$Temperature[l +
i]) * Threshold.TTS$TimeDiff[l + i])}
        YearlyBtwnOutput[k, 4] = g1
        YearlyBtwnOutput[k, 5] = g2
        YearlyBtwnOutput[k, 6] = g3

        for (d in 1 : (length(Btwn.threshold) / 2)) {
                a = 0; b = 0; e = 0; f = 0; g = 0
                flist <- as.vector(0)
        for (l in 0 : (j - 1)) {
                if (l == 0) {
                        if (difftime(Threshold.TTS$DateTime[l + i +
1],Threshold.TTS$DateTime[l + i], units = "hours") < ShortGap)
{Threshold.TTS$TimeDiff[l + i] <- difftime(Threshold.TTS$DateTime[l + i + 1],
Threshold.TTS$DateTime[l + i], units = "hours")} else {Threshold.TTS$TimeDiff[l
+ i] <- ShortGap}
                        if (Threshold.TTS$Temperature[l + i] > Btwn.threshold[d,
1] & Threshold.TTS$Temperature[l + i] < Btwn.threshold[d, 2]) {a = a + 1}
                        if (Threshold.TTS$Temperature[l + i] > Btwn.threshold[d,
1] & Threshold.TTS$Temperature[l + i] < Btwn.threshold[d, 2]) {b = b +
as.numeric(Threshold.TTS$TimeDiff[l + i])}
                        if (Threshold.TTS$Temperature[l + i] > Btwn.threshold[d,
1] & Threshold.TTS$Temperature[l + i] < Btwn.threshold[d, 2]) {f = f +
(as.numeric(Threshold.TTS$TimeDiff[l + i]))} else {flist = c(flist, f); f = 0}
                        if (Threshold.TTS$Temperature[l + i] > Btwn.threshold[d,
1] & Threshold.TTS$Temperature[l + i] < Btwn.threshold[d, 2]) {if
(Btwn.threshold[d, 1] != -Inf) {e = e + ((Threshold.TTS$Temperature[l + i] -
Btwn.threshold[d, 1]) * Threshold.TTS$TimeDiff[l + i])} else {e = e +
(Btwn.threshold[d, 2] - Threshold.TTS$Temperature[l + i]) *
Threshold.TTS$TimeDiff[l + i]}}}
                if (l == (j - 1)) {
                        if (difftime(Threshold.TTS$DateTime[l +
i],Threshold.TTS$DateTime[l + i - 1], units = "hours") < ShortGap)
{Threshold.TTS$TimeDiff[l + i] <- difftime(Threshold.TTS$DateTime[l +
i],Threshold.TTS$DateTime[l + i - 1], units = "hours")} else
{Threshold.TTS$TimeDiff[l + i] <- ShortGap}
                        if (Threshold.TTS$Temperature[l + i] > Btwn.threshold[d,
1] & Threshold.TTS$Temperature[l + i] < Btwn.threshold[d, 2] &
(Threshold.TTS$Temperature[l + i - 1] <= Btwn.threshold[d, 1] |
Threshold.TTS$Temperature[l + i - 1] >= Btwn.threshold[d, 2])) {a = a + 1}
                        if (Threshold.TTS$Temperature[l + i] > Btwn.threshold[d,
1] & Threshold.TTS$Temperature[l + i] < Btwn.threshold[d, 2]) {b = b +
as.numeric(Threshold.TTS$TimeDiff[l + i])}
                        if (Threshold.TTS$Temperature[l + i] > Btwn.threshold[d,
1] & Threshold.TTS$Temperature[l + i] < Btwn.threshold[d, 2]) {f = f +
(as.numeric(Threshold.TTS$TimeDiff[l + i])); flist=c(flist,f)} else {flist =
c(flist, f); f = 0}
                        if (Threshold.TTS$Temperature[l + i] > Btwn.threshold[d,
1] & Threshold.TTS$Temperature[l + i] < Btwn.threshold[d, 2]) {if
(Btwn.threshold[d, 1] != -Inf) {e = e + ((Threshold.TTS$Temperature[l + i] -
Btwn.threshold[d, 1]) * Threshold.TTS$TimeDiff[l + i])} else {e = e +
```

```
(Btwn.threshold[d, 2] - Threshold.TTS$Temperature[l + i]) *
Threshold.TTS$TimeDiff[l + i]}}}
                if (l != 0 & l != (j - 1)) {
                        if (difftime(Threshold.TTS$DateTime[l +
i],Threshold.TTS$DateTime[l + i - 1], units = "hours") < ShortGap)
{Threshold.TTS$TimeDiff[l + i] <- difftime(Threshold.TTS$DateTime[l +
i],Threshold.TTS$DateTime[l + i - 1], units = "hours")} else
{Threshold.TTS$TimeDiff[l + i] <- ShortGap}
                        if (Threshold.TTS$Temperature[l + i] > Btwn.threshold[d,
1] & Threshold.TTS$Temperature[l + i] < Btwn.threshold[d, 2] &
(Threshold.TTS$Temperature[l + i - 1] <= Btwn.threshold[d, 1] |
Threshold.TTS$Temperature[l + i - 1] >= Btwn.threshold[d, 2])) {a = a + 1}
                        if (Threshold.TTS$Temperature[l + i] > Btwn.threshold[d,
1] & Threshold.TTS$Temperature[l + i] < Btwn.threshold[d, 2]) {b = b +
as.numeric(Threshold.TTS$TimeDiff[l + i])}
                        if (Threshold.TTS$Temperature[l + i] > Btwn.threshold[d,
1] & Threshold.TTS$Temperature[l + i] < Btwn.threshold[d, 2]) {f = f +
(as.numeric(Threshold.TTS$TimeDiff[l + i]))} else {flist = c(flist, f); f = 0}
                        if (Threshold.TTS$Temperature[l + i] > Btwn.threshold[d,
1] & Threshold.TTS$Temperature[l + i] < Btwn.threshold[d, 2]) {if
(Btwn.threshold[d, 1] != -Inf) {e = e + ((Threshold.TTS$Temperature[l + i] -
Btwn.threshold[d, 1]) * Threshold.TTS$TimeDiff[l + i])} else {e = e +
(Btwn.threshold[d, 2] - Threshold.TTS$Temperature[l + i]) *
Threshold.TTS$TimeDiff[l + i]}}}}
        YearlyBtwnOutput[k, 7 + (4 * (d - 1))] = a
        YearlyBtwnOutput[k, 8 + (4 * (d - 1))] = b
        YearlyBtwnOutput[k, 9 + (4 * (d - 1))] = max(flist)
        YearlyBtwnOutput[k, 10 + (4 * (d - 1))] = e
}
i = i + j; j = 0; k = k + 1}}

# Run calculations just for the last year to complete the set of analyses
        j=length(Threshold.TTS$Temperature[Threshold.TTS$Year ==
Threshold.TTS$Year[i]])
        YearlyBtwnOutput[k, 1] = as.numeric(paste(Site[ColNumber], ".",
SegNumber, sep = ""))
        YearlyBtwnOutput[k, 2] = Threshold.TTS$Year[i]
        YearlyBtwnOutput[k, 3] =
length(Threshold.TTS$Temperature[Threshold.TTS$Year == Threshold.TTS$Year[i]])

        # loop through all records and sum up responses
        g1 = 0; g2 = 0; g3 = 0
        for (l in 0 : (j - 1)) {
                if (Threshold.TTS$TimeChange[l + i] > ShortGap)
{Threshold.TTS$TimeChange[l + i] <- ShortGap}
                g1 = g1 + (TemperatureResponse1(Threshold.TTS$Temperature[l +
i]) * Threshold.TTS$TimeDiff[l + i])
                g2 = g2 + (TemperatureResponse2(Threshold.TTS$Temperature[l +
i]) * Threshold.TTS$TimeDiff[l + i])
                g3 = g3 + (TemperatureResponse3(Threshold.TTS$Temperature[l +
i]) * Threshold.TTS$TimeDiff[l + i])}
        YearlyBtwnOutput[k, 4] = g1
```

```
        YearlyBtwnOutput[k, 5] = g2
        YearlyBtwnOutput[k, 6] = g3

        for (d in 1:(length(Btwn.threshold) / 2)) {
                a = 0; b = 0; e = 0; f = 0; g = 0
                flist <- as.vector(0)
        for (l in 0 : (j - 1)) {
                if (l == 0) {
                        if (difftime(Threshold.TTS$DateTime[l + i +
1],Threshold.TTS$DateTime[l + i], units = "hours") < ShortGap)
{Threshold.TTS$TimeDiff[l + i] <- difftime(Threshold.TTS$DateTime[l + i +
1],Threshold.TTS$DateTime[l + i], units = "hours")} else
{Threshold.TTS$TimeDiff[l + i] <- ShortGap}
                        if (Threshold.TTS$Temperature[l + i] > Btwn.threshold[d,
1] & Threshold.TTS$Temperature[l + i] < Btwn.threshold[d, 2]) {a = a + 1}
                        if (Threshold.TTS$Temperature[l + i] > Btwn.threshold[d,
1] & Threshold.TTS$Temperature[l + i] < Btwn.threshold[d, 2]) {b = b +
as.numeric(Threshold.TTS$TimeDiff[l + i])}
                        if (Threshold.TTS$Temperature[l + i] > Btwn.threshold[d,
1] & Threshold.TTS$Temperature[l + i] < Btwn.threshold[d, 2]) {f = f +
(as.numeric(Threshold.TTS$TimeDiff[l + i]))} else {flist = c(flist, f); f = 0}
                        if (Threshold.TTS$Temperature[l + i] > Btwn.threshold[d,
1] & Threshold.TTS$Temperature[l + i] < Btwn.threshold[d, 2]) {if
(Btwn.threshold[d, 1] != -Inf) {e = e + ((Threshold.TTS$Temperature[l + i] -
Btwn.threshold[d, 1]) * Threshold.TTS$TimeDiff[l + i])} else {e = e +
(Btwn.threshold[d, 2] - Threshold.TTS$Temperature[l + i]) *
Threshold.TTS$TimeDiff[l + i]}}}
                if (l == (j - 1)) {
                        if (difftime(Threshold.TTS$DateTime[l +
i],Threshold.TTS$DateTime[l + i - 1], units = "hours") < ShortGap)
{Threshold.TTS$TimeDiff[l + i] <- difftime(Threshold.TTS$DateTime[l +
i],Threshold.TTS$DateTime[l + i - 1], units = "hours")} else
{Threshold.TTS$TimeDiff[l + i] <- ShortGap}
                        if (Threshold.TTS$Temperature[l + i] > Btwn.threshold[d,
1] & Threshold.TTS$Temperature[l + i] < Btwn.threshold[d, 2] &
(Threshold.TTS$Temperature[l + i - 1] <= Btwn.threshold[d, 1] |
Threshold.TTS$Temperature[l + i - 1] >= Btwn.threshold[d, 2])) {a = a + 1}
                        if (Threshold.TTS$Temperature[l + i] > Btwn.threshold[d,
1] & Threshold.TTS$Temperature[l + i] < Btwn.threshold[d, 2]) {b = b +
as.numeric(Threshold.TTS$TimeDiff[l + i])}
                        if (Threshold.TTS$Temperature[l + i] > Btwn.threshold[d,
1] & Threshold.TTS$Temperature[l + i] < Btwn.threshold[d, 2]) {f = f +
(as.numeric(Threshold.TTS$TimeDiff[l + i])); flist=c(flist,f)} else {flist =
c(flist, f); f = 0}
                        if (Threshold.TTS$Temperature[l + i] > Btwn.threshold[d,
1] & Threshold.TTS$Temperature[l + i] < Btwn.threshold[d, 2]) {if
(Btwn.threshold[d, 1] != -Inf) {e = e + ((Threshold.TTS$Temperature[l + i] -
Btwn.threshold[d, 1]) * Threshold.TTS$TimeDiff[l + i])} else {e = e +
(Btwn.threshold[d, 2] - Threshold.TTS$Temperature[l + i]) *
Threshold.TTS$TimeDiff[l + i]}}}
                if (l != 0 & l != (j - 1)) {
                        if (difftime(Threshold.TTS$DateTime[l +
```

```
i],Threshold.TTS$DateTime[l + i - 1], units = "hours") < ShortGap)
{Threshold.TTS$TimeDiff[l + i] <- difftime(Threshold.TTS$DateTime[l +
i],Threshold.TTS$DateTime[l + i - 1], units = "hours")} else
{Threshold.TTS$TimeDiff[l + i] <- ShortGap}
                        if (Threshold.TTS$Temperature[l + i] > Btwn.threshold[d,
1] & Threshold.TTS$Temperature[l + i] < Btwn.threshold[d, 2] &
(Threshold.TTS$Temperature[l + i - 1] <= Btwn.threshold[d, 1] |
Threshold.TTS$Temperature[l + i - 1] >= Btwn.threshold[d, 2])) {a = a + 1}
                        if (Threshold.TTS$Temperature[l + i] > Btwn.threshold[d,
1] & Threshold.TTS$Temperature[l + i] < Btwn.threshold[d, 2]) {b = b +
as.numeric(Threshold.TTS$TimeDiff[l + i])}
                        if (Threshold.TTS$Temperature[l + i] > Btwn.threshold[d,
1] & Threshold.TTS$Temperature[l + i] < Btwn.threshold[d, 2]) {f = f +
(as.numeric(Threshold.TTS$TimeDiff[l + i]))} else {flist = c(flist, f); f = 0}
                        if (Threshold.TTS$Temperature[l + i] > Btwn.threshold[d,
1] & Threshold.TTS$Temperature[l + i] < Btwn.threshold[d, 2]) {if
(Btwn.threshold[d, 1] != -Inf) {e = e + ((Threshold.TTS$Temperature[l + i] -
Btwn.threshold[d, 1]) * Threshold.TTS$TimeDiff[l + i])} else {e = e +
(Btwn.threshold[d, 2] - Threshold.TTS$Temperature[l + i]) *
Threshold.TTS$TimeDiff[l + i]}}}}
        YearlyBtwnOutput[k, 7 + (4 * (d - 1))] = a
        YearlyBtwnOutput[k, 8 + (4 * (d - 1))] = b
        YearlyBtwnOutput[k, 9 + (4 * (d - 1))] = max(flist)
        YearlyBtwnOutput[k, 10 + (4 * (d - 1))] = e
}
if (file.exists(paste(OutputFileName, "_AnnualThresholds.csv", sep = "")))
{write.table(YearlyBtwnOutput, file = paste(OutputFileName,
"_AnnualThresholds.csv", sep = ""), append = TRUE, sep = ",", dec=".", quote =
FALSE, col.names = FALSE, row.names = FALSE)} else
{write.table(YearlyBtwnOutput, file = paste(OutputFileName,
"_AnnualThresholds.csv", sep = ""), append = FALSE, sep = ",", dec=".", quote =
FALSE, col.names = TRUE, row.names = FALSE)}
remove(YearlyBtwnOutput, a, b, e, f, g1, g2, g3)}



# Calculate the threshold related paramters per month and year
if (Do.MonthlyBtwnOutput == "y") {
i = 1; j = 0; k = 1
while (i + j <= length(Threshold.TTS$Temperature) ) {
        if (Threshold.TTS$Year[i] == Threshold.TTS$Year[i + j] &
Threshold.TTS$Month[i] == Threshold.TTS$Month[i + j]) {j = j + 1} else {
        MonthlyBtwnOutput[k, 1] = as.numeric(paste(Site[ColNumber], ".",
SegNumber, sep = ""))
        MonthlyBtwnOutput[k, 2] = Threshold.TTS$Year[i]
        MonthlyBtwnOutput[k, 3] = Threshold.TTS$Month[i]
        MonthlyBtwnOutput[k, 4] =
length(Threshold.TTS$Temperature[Threshold.TTS$Year == Threshold.TTS$Year[i] &
Threshold.TTS$Month == Threshold.TTS$Month[i]])

        # loop through all records and sum up responses
        g1 = 0; g2 = 0; g3 = 0
```

```
        for (l in 0 : (j - 1)) {
                if (Threshold.TTS$TimeChange[l + i] > ShortGap)
{Threshold.TTS$TimeChange[l + i] <- ShortGap}
                g1 = g1 + (TemperatureResponse1(Threshold.TTS$Temperature[l +
i]) * Threshold.TTS$TimeDiff[l + i])
                g2 = g2 + (TemperatureResponse2(Threshold.TTS$Temperature[l +
i]) * Threshold.TTS$TimeDiff[l + i])
                g3 = g3 + (TemperatureResponse3(Threshold.TTS$Temperature[l +
i]) * Threshold.TTS$TimeDiff[l + i])}
        MonthlyBtwnOutput[k, 5] = g1
        MonthlyBtwnOutput[k, 6] = g2
        MonthlyBtwnOutput[k, 7] = g3

        for (d in 1:(length(Btwn.threshold) / 2)) {
                a = 0; b = 0; e = 0; f = 0; g = 0
                flist <- as.vector(0)
        for (l in 0 : (j - 1)) {
                if (l == 0) {
                        if (difftime(Threshold.TTS$DateTime[l + i +
1],Threshold.TTS$DateTime[l + i], units = "hours") < ShortGap)
{Threshold.TTS$TimeDiff[l + i] <- difftime(Threshold.TTS$DateTime[l + i +
1],Threshold.TTS$DateTime[l + i], units = "hours")} else
{Threshold.TTS$TimeDiff[l + i] <- ShortGap}
                        if (Threshold.TTS$Temperature[l + i] > Btwn.threshold[d,
1] & Threshold.TTS$Temperature[l + i] < Btwn.threshold[d, 2]) {a = a + 1}
                        if (Threshold.TTS$Temperature[l + i] > Btwn.threshold[d,
1] & Threshold.TTS$Temperature[l + i] < Btwn.threshold[d, 2]) {b = b +
as.numeric(Threshold.TTS$TimeDiff[l + i])}
                        if (Threshold.TTS$Temperature[l + i] > Btwn.threshold[d,
1] & Threshold.TTS$Temperature[l + i] < Btwn.threshold[d, 2]) {f = f +
(as.numeric(Threshold.TTS$TimeDiff[l + i]))} else {flist = c(flist, f); f = 0}
                        if (Threshold.TTS$Temperature[l + i] > Btwn.threshold[d,
1] & Threshold.TTS$Temperature[l + i] < Btwn.threshold[d, 2]) {if
(Btwn.threshold[d, 1] != -Inf) {e = e + ((Threshold.TTS$Temperature[l + i] -
Btwn.threshold[d, 1]) * Threshold.TTS$TimeDiff[l + i])} else {e = e +
(Btwn.threshold[d, 2]-(Threshold.TTS$Temperature[l + i]) *
Threshold.TTS$TimeDiff[l + i])}}}
                if (l == (j - 1)) {
                        if (difftime(Threshold.TTS$DateTime[l +
i],Threshold.TTS$DateTime[l + i - 1], units = "hours") < ShortGap)
{Threshold.TTS$TimeDiff[l + i] <- difftime(Threshold.TTS$DateTime[l +
i],Threshold.TTS$DateTime[l + i - 1], units = "hours")} else
{Threshold.TTS$TimeDiff[l + i] <- ShortGap}
                        if (Threshold.TTS$Temperature[l + i] > Btwn.threshold[d,
1] & Threshold.TTS$Temperature[l + i] < Btwn.threshold[d, 2] &
(Threshold.TTS$Temperature[l + i - 1] <= Btwn.threshold[d, 1] |
Threshold.TTS$Temperature[l + i - 1] >= Btwn.threshold[d, 2])) {a = a + 1}
                        if (Threshold.TTS$Temperature[l + i] > Btwn.threshold[d,
1] & Threshold.TTS$Temperature[l + i] < Btwn.threshold[d, 2]) {b = b +
as.numeric(Threshold.TTS$TimeDiff[l + i])}
                        if (Threshold.TTS$Temperature[l + i] > Btwn.threshold[d,
1] & Threshold.TTS$Temperature[l + i] < Btwn.threshold[d, 2]) {f = f +
```

```
(as.numeric(Threshold.TTS$TimeDiff[l + i])); flist=c(flist,f)} else {flist =
c(flist, f); f = 0}
                        if (Threshold.TTS$Temperature[l + i] > Btwn.threshold[d,
1] & Threshold.TTS$Temperature[l + i] < Btwn.threshold[d, 2]) {if
(Btwn.threshold[d, 1] != -Inf) {e = e + ((Threshold.TTS$Temperature[l + i] -
Btwn.threshold[d, 1]) * Threshold.TTS$TimeDiff[l + i])} else {e = e +
(Btwn.threshold[d, 2]-(Threshold.TTS$Temperature[l + i]) *
Threshold.TTS$TimeDiff[l + i])}}}
                if (l != 0 & l != (j - 1)) {
                        if (difftime(Threshold.TTS$DateTime[l +
i],Threshold.TTS$DateTime[l + i - 1], units = "hours") < ShortGap)
{Threshold.TTS$TimeDiff[l + i] <- difftime(Threshold.TTS$DateTime[l +
i],Threshold.TTS$DateTime[l + i - 1], units = "hours")} else
{Threshold.TTS$TimeDiff[l + i] <- ShortGap}
                        if (Threshold.TTS$Temperature[l + i] > Btwn.threshold[d,
1] & Threshold.TTS$Temperature[l + i] < Btwn.threshold[d, 2] &
(Threshold.TTS$Temperature[l + i - 1] <= Btwn.threshold[d, 1] |
Threshold.TTS$Temperature[l + i - 1] >= Btwn.threshold[d, 2])) {a = a + 1}
                        if (Threshold.TTS$Temperature[l + i] > Btwn.threshold[d,
1] & Threshold.TTS$Temperature[l + i] < Btwn.threshold[d, 2]) {b = b +
as.numeric(Threshold.TTS$TimeDiff[l + i])}
                        if (Threshold.TTS$Temperature[l + i] > Btwn.threshold[d,
1] & Threshold.TTS$Temperature[l + i] < Btwn.threshold[d, 2]) {f = f +
(as.numeric(Threshold.TTS$TimeDiff[l + i]))} else {flist = c(flist, f); f = 0}
                        if (Threshold.TTS$Temperature[l + i] > Btwn.threshold[d,
1] & Threshold.TTS$Temperature[l + i] < Btwn.threshold[d, 2]) {if
(Btwn.threshold[d, 1] != -Inf) {e = e + ((Threshold.TTS$Temperature[l + i] -
Btwn.threshold[d, 1]) * Threshold.TTS$TimeDiff[l + i])} else {e = e +
(Btwn.threshold[d, 2]-(Threshold.TTS$Temperature[l + i]) *
Threshold.TTS$TimeDiff[l + i])}}}}
        MonthlyBtwnOutput[k, 8 + (4 * (d - 1))] = a
        MonthlyBtwnOutput[k, 9 + (4 * (d - 1))] = b
        MonthlyBtwnOutput[k, 10 + (4 * (d - 1))] = max(flist)
        MonthlyBtwnOutput[k, 11 + (4 * (d - 1))] = e
}
i = i + j; j = 0; k = k + 1}}

        j=length(Threshold.TTS$Temperature[Threshold.TTS$Year ==
Threshold.TTS$Year[i] & Threshold.TTS$Month == Threshold.TTS$Month[i]])
        MonthlyBtwnOutput[k, 1] = as.numeric(paste(Site[ColNumber], ".",
SegNumber, sep = ""))
        MonthlyBtwnOutput[k, 2] = Threshold.TTS$Year[i]
        MonthlyBtwnOutput[k, 3] = Threshold.TTS$Month[i]
        MonthlyBtwnOutput[k, 4] =
length(Threshold.TTS$Temperature[Threshold.TTS$Year == Threshold.TTS$Year[i] &
Threshold.TTS$Month == Threshold.TTS$Month[i]])

        # loop through all records and sum up responses
        g1 = 0; g2 = 0; g3 = 0
        for (l in 0 : (j - 1)) {
                if (Threshold.TTS$TimeChange[l + i] > ShortGap)
{Threshold.TTS$TimeChange[l + i] <- ShortGap}
```

```
                g1 = g1 + (TemperatureResponse1(Threshold.TTS$Temperature[l +
i]) * Threshold.TTS$TimeDiff[l + i])
                g2 = g2 + (TemperatureResponse2(Threshold.TTS$Temperature[l +
i]) * Threshold.TTS$TimeDiff[l + i])
                g3 = g3 + (TemperatureResponse3(Threshold.TTS$Temperature[l +
i]) * Threshold.TTS$TimeDiff[l + i])}
        MonthlyBtwnOutput[k, 5] = g1
        MonthlyBtwnOutput[k, 6] = g2
        MonthlyBtwnOutput[k, 7] = g3

        for (d in 1:(length(Btwn.threshold) / 2)) {
                a = 0; b = 0; e = 0; f = 0; g = 0
                flist <- as.vector(0)
        for (l in 0 : (j - 1)) {
                if (l == 0) {
                        if (difftime(Threshold.TTS$DateTime[l + i +
1],Threshold.TTS$DateTime[l + i], units = "hours") < ShortGap)
{Threshold.TTS$TimeDiff[l + i] <- difftime(Threshold.TTS$DateTime[l + i +
1],Threshold.TTS$DateTime[l + i], units = "hours")} else
{Threshold.TTS$TimeDiff[l + i] <- ShortGap}
                        if (Threshold.TTS$Temperature[l + i] > Btwn.threshold[d,
1] & Threshold.TTS$Temperature[l + i] < Btwn.threshold[d, 2]) {a = a + 1}
                        if (Threshold.TTS$Temperature[l + i] > Btwn.threshold[d,
1] & Threshold.TTS$Temperature[l + i] < Btwn.threshold[d, 2]) {b = b +
as.numeric(Threshold.TTS$TimeDiff[l + i])}
                        if (Threshold.TTS$Temperature[l + i] > Btwn.threshold[d,
1] & Threshold.TTS$Temperature[l + i] < Btwn.threshold[d, 2]) {f = f +
(as.numeric(Threshold.TTS$TimeDiff[l + i]))} else {flist = c(flist, f); f = 0}
                        if (Threshold.TTS$Temperature[l + i] > Btwn.threshold[d,
1] & Threshold.TTS$Temperature[l + i] < Btwn.threshold[d, 2]) {if
(Btwn.threshold[d, 1] != -Inf) {e = e + ((Threshold.TTS$Temperature[l + i] -
Btwn.threshold[d, 1]) * Threshold.TTS$TimeDiff[l + i])} else {e = e +
(Btwn.threshold[d, 2] - Threshold.TTS$Temperature[l + i]) *
Threshold.TTS$TimeDiff[l + i]}}}
                if (l == (j - 1)) {
                        if (difftime(Threshold.TTS$DateTime[l +
i],Threshold.TTS$DateTime[l + i - 1], units = "hours") < ShortGap)
{Threshold.TTS$TimeDiff[l + i] <- difftime(Threshold.TTS$DateTime[l +
i],Threshold.TTS$DateTime[l + i - 1], units = "hours")} else
{Threshold.TTS$TimeDiff[l + i] <- ShortGap}
                        if (Threshold.TTS$Temperature[l + i] > Btwn.threshold[d,
1] & Threshold.TTS$Temperature[l + i] < Btwn.threshold[d, 2] &
(Threshold.TTS$Temperature[l + i - 1] <= Btwn.threshold[d, 1] |
Threshold.TTS$Temperature[l + i - 1] >= Btwn.threshold[d, 2])) {a = a + 1}
                        if (Threshold.TTS$Temperature[l + i] > Btwn.threshold[d,
1] & Threshold.TTS$Temperature[l + i] < Btwn.threshold[d, 2]) {b = b +
as.numeric(Threshold.TTS$TimeDiff[l + i])}
                        if (Threshold.TTS$Temperature[l + i] > Btwn.threshold[d,
1] & Threshold.TTS$Temperature[l + i] < Btwn.threshold[d, 2]) {f = f +
(as.numeric(Threshold.TTS$TimeDiff[l + i])); flist=c(flist,f)} else {flist =
c(flist, f); f = 0}
                        if (Threshold.TTS$Temperature[l + i] > Btwn.threshold[d,
```

```
1] & Threshold.TTS$Temperature[l + i] < Btwn.threshold[d, 2]) {if
(Btwn.threshold[d, 1] != -Inf) {e = e + ((Threshold.TTS$Temperature[l + i] -
Btwn.threshold[d, 1]) * Threshold.TTS$TimeDiff[l + i])} else {e = e +
(Btwn.threshold[d, 2] - Threshold.TTS$Temperature[l + i]) *
Threshold.TTS$TimeDiff[l + i]}}}
                if (l != 0 & l != (j - 1)) {
                        if (difftime(Threshold.TTS$DateTime[l +
i],Threshold.TTS$DateTime[l + i - 1], units = "hours") < ShortGap)
{Threshold.TTS$TimeDiff[l + i] <- difftime(Threshold.TTS$DateTime[l +
i],Threshold.TTS$DateTime[l + i - 1], units = "hours")} else
{Threshold.TTS$TimeDiff[l + i] <- ShortGap}
                        if (Threshold.TTS$Temperature[l + i] > Btwn.threshold[d,
1] & Threshold.TTS$Temperature[l + i] < Btwn.threshold[d, 2] &
(Threshold.TTS$Temperature[l + i - 1] <= Btwn.threshold[d, 1] |
Threshold.TTS$Temperature[l + i - 1] >= Btwn.threshold[d, 2])) {a = a + 1}
                        if (Threshold.TTS$Temperature[l + i] > Btwn.threshold[d,
1] & Threshold.TTS$Temperature[l + i] < Btwn.threshold[d, 2]) {b = b +
as.numeric(Threshold.TTS$TimeDiff[l + i])}
                        if (Threshold.TTS$Temperature[l + i] > Btwn.threshold[d,
1] & Threshold.TTS$Temperature[l + i] < Btwn.threshold[d, 2]) {f = f +
(as.numeric(Threshold.TTS$TimeDiff[l + i]))} else {flist = c(flist, f); f = 0}
                        if (Threshold.TTS$Temperature[l + i] > Btwn.threshold[d,
1] & Threshold.TTS$Temperature[l + i] < Btwn.threshold[d, 2]) {if
(Btwn.threshold[d, 1] != -Inf) {e = e + ((Threshold.TTS$Temperature[l + i] -
Btwn.threshold[d, 1]) * Threshold.TTS$TimeDiff[l + i])} else {e = e +
(Btwn.threshold[d, 2] - Threshold.TTS$Temperature[l + i]) *
Threshold.TTS$TimeDiff[l + i]}}}}
        MonthlyBtwnOutput[k, 8 + (4 * (d - 1))] = a
        MonthlyBtwnOutput[k, 9 + (4 * (d - 1))] = b
        MonthlyBtwnOutput[k, 10 + (4 * (d - 1))] = max(flist)
        MonthlyBtwnOutput[k, 11 + (4 * (d - 1))] = e
}
if (file.exists(paste(OutputFileName, "_MonthlyThresholds.csv", sep = "")))
{write.table(MonthlyBtwnOutput, file = paste(OutputFileName,
"_MonthlyThresholds.csv", sep = ""), append = TRUE, sep = ",", dec=".", quote =
FALSE, col.names = FALSE, row.names = FALSE)} else
{write.table(MonthlyBtwnOutput, file = paste(OutputFileName,
"_MonthlyThresholds.csv", sep = ""), append = FALSE, sep = ",", dec=".", quote =
FALSE, col.names = TRUE, row.names = FALSE)}
remove(MonthlyBtwnOutput, a, b, e, f, g1, g2, g3)}
}


#####################################
# 10. Calculate developmental times #
#####################################

# For these calculations, the code loops through each point in the time series,
using each time as a starting point for calculations.
# For each starting point (day or hour), hour degrees are summed (i.e. the
temperature value greater than the specified LDT) until the cumulative total
meets or exceeds the specified SET (sum of effective temperatures) threshold. At
```

that point, adequate thermal energy has been experienced for the development of one generation from eggs.

```
if (Do.DevelopmentalTimes == "y") {

# Choose time series (i.e. longest duration and/or interpolation), as specified
earlier by the user in section 2.b of the code.
if (DevTimes.Use.TTS == "y") {DevTimes.TTS <- TTS}
if (DevTimes.Use.LongestDuration == "y") {DevTimes.TTS <- LongestDuration}
if (DevTimes.Use.Interpolated.TTS == "y") {DevTimes.TTS <- Interpolated.TTS}
if (DevTimes.Use.Interpolated.Sinus.TTS == "y") {DevTimes.TTS <-
Interpolated.Sinus.TTS}
if (DevTimes.Use.Interpolated.LongestDuration == "y") {DevTimes.TTS <-
Interpolated.LongestDuration.TTS}

DevelopmentTimes <- matrix(data = -2, nrow = length(DevTimes.TTS$Temperature),
ncol = length(LDT))
DevelopmentRecords <- matrix(data = -2, nrow = length(DevTimes.TTS$Temperature),
ncol = length(LDT))
SET.contrib <- matrix(data = 0, nrow = length(DevTimes.TTS$Temperature), ncol =
length(LDT))
Mortality.events <- matrix(data = NA, ncol = length(LDT), nrow =
length(DevTimes.TTS$Temperature))
Mortality.events.record <- list()
DevTimes.ColNames <- vector(length = length(length(LDT)))
DevTimes.ColNames.SET <- vector(length = length(length(LDT)))
for (NumberLDT in 1 : length(LDT)) {
        DevTimes.ColNames <- SET.labs}

for (NumberLDT in 1 : length(LDT)) {
        DevTimes.ColNames.SET[NumberLDT] <- paste("SET=", SET[NumberLDT], ";
LDT=", LDT[NumberLDT], sep = "")}

colnames(DevelopmentTimes) <- paste("DevDays_", DevTimes.ColNames, sep = "")
colnames(DevelopmentRecords) <- paste("DevRecs_", DevTimes.ColNames, sep = "")
colnames(SET.contrib) <- paste("SET.contrib_", DevTimes.ColNames, sep = "")
DevTimes.TTS$TimeChange[1] <- DevTimes.TTS$TimeChange[2]

DevTime.Temperature.Temporary <- as.vector(DevTimes.TTS$Temperature)

# Loop through time series records
for (NumberLDT in 1 : length(LDT)) {
break.loop <- FALSE
for (i in 1 : length(DevTime.Temperature.Temporary)) {
  SET.temp <- 0
        l <- 0                            # l = number of periods required until
developmental threshold (SET) met
        while (SET.temp < SET[NumberLDT]) {
                if ((DevTime.Temperature.Temporary[i + l] > LDT[NumberLDT]) &
(DevTime.Temperature.Temporary[i + l] < UDT[NumberLDT])) {
        SET.temp = SET.temp + ((DevTime.Temperature.Temporary[i + l] -
LDT[NumberLDT]) * DevTimes.TTS$TimeChange[i + l])}
```

```
                if (i + l >= length(DevTime.Temperature.Temporary)) {
        l = -2; break.loop <- TRUE; break}
                if ((DevTime.Temperature.Temporary[i + l] <= LLT[NumberLDT]) |
(DevTime.Temperature.Temporary[i + l] >= ULT[NumberLDT])) {
        l = -1; break}
                if (break.loop) {break}
                l = l + 1}
        DevelopmentRecords[i, NumberLDT] <- l
        if (break.loop) {break}
        if (l > 0) {DevelopmentTimes[i, NumberLDT] =
difftime(DevTimes.TTS$DateTime[i + l - 1], DevTimes.TTS$DateTime[i], units =
"days")} else {DevelopmentTimes[i, NumberLDT] = -1}
}

# Create a summary plot. In the fourth panel of the figure the mean
developmental period is only shown for the months in which hatching will lead to
successful development.
if (Do.DevTimes.Plot == "y") {
        tiff(paste(OutputFileName,
"_DevelopmentTimes_summary_Site_",Site[ColNumber], ".", SegNumber, "_",
DevTimes.ColNames[NumberLDT], "_", NumberLDT, ".tif", sep = ""), res = 150,
units = "cm", width = 18, height = 18)
        par(mfrow = c(2,2), mar = c(4, 4, 3, 2) + 0.1)
        if (length(DevelopmentTimes[DevelopmentTimes[, NumberLDT] > 0]) > 0) {
        plot(DevTimes.TTS$DateTime[DevelopmentRecords[, NumberLDT] >= -1],
DevTime.Temperature.Temporary[DevelopmentRecords[, NumberLDT] >= -1], type =
"l", xlab = "Date", ylab = "Temperature")
        abline(h = LDT[NumberLDT], lty = 3, col = "red")
        abline(h = UDT[NumberLDT], lty = 3, col = "red")
        abline(h = LLT[NumberLDT], col = "red")
        abline(h = ULT[NumberLDT], col = "red")
        boxplot(DevTime.Temperature.Temporary[DevelopmentRecords[, NumberLDT] >=
-1] ~ DevTimes.TTS$Month[DevelopmentRecords[, NumberLDT] >= -1], xlab = "Month",
ylab = "Mean temperature")
        ylim1 <- c(0, 1.1 * max(DevelopmentTimes[,
NumberLDT][is.na(DevelopmentTimes[, NumberLDT]) == FALSE]))
        if (ylim1[2] == -Inf) {
                plot(0 : 2, 0 : 2, type = "n", xlab = "Date", ylab =
"Development time")
                text(x = 1, y = 1.2, "Time series not long enough")
                text(x = 1, y = 0.9, "for any individuals to develop")
                mtext(SET.spp[NumberLDT], side = 3, line = 1)
        } else {
                plot(DevTimes.TTS$DateTime[DevelopmentTimes[,NumberLDT] > 0],
DevelopmentTimes[, NumberLDT][DevelopmentTimes[, NumberLDT] > 0], type = "l",
xlab = "Date", ylab = "Development time (days)", ylim = ylim1)
                abline(v = DevTimes.TTS$DateTime[DevelopmentRecords[, NumberLDT]
< 0], col = "red")
                abline(v = DevTimes.TTS$DateTime[DevelopmentRecords[, NumberLDT]
< (-1)], col = "white")
                mtext(SET.spp[NumberLDT], side = 3, line = 1)
                box("plot")}
```

```
        boxplot(DevelopmentTimes[, NumberLDT][DevelopmentTimes[, NumberLDT] > 0]
~ DevTimes.TTS$Month[DevelopmentTimes[, NumberLDT] > 0], xlab = "Month", ylab =
"Mean development time (days)")
        mtext(paste("SET = ", SET[NumberLDT], "LDT = ", LDT[NumberLDT]), side =
3, line = 1)
        dev.off()
} else {
        plot(DevTimes.TTS$DateTime, DevTimes.TTS$Temperature, type = "l", xlab =
"Date", ylab = "Temperature")
        abline(h = LDT[NumberLDT], lty = 3, col = "red")
        boxplot(DevTimes.TTS$Temperature ~ DevTimes.TTS$Month, xlab = "Month",
ylab = "Mean temperature")
        abline(h = LDT[NumberLDT], lty = 3, col = "red")
        plot(0 : 2, 0 : 2, type = "n", xlab = "Date", ylab = "Development time")
        mtext(SET.spp[NumberLDT], side = 3, line = 1)
        text(x = 1, y = 1.2, "Time series not long enough")
        text(x = 1, y = 0.9, "for any individuals to develop")
        dev.off()}}
}

# Output raw development times data to be available for plotting.
if (file.exists(paste(OutputFileName, "_DevelopmentTimes.csv", sep = ""))) {
    write.table(cbind(DevTimes.TTS, DevelopmentTimes), file =
paste(OutputFileName, "_DevelopmentTimes.csv", sep = ""), append = TRUE, sep =
",", dec = ".", quote = FALSE, col.names = FALSE, row.names = FALSE)
} else {
    write.table(cbind(DevTimes.TTS, DevelopmentTimes), file =
paste(OutputFileName, "_DevelopmentTimes.csv", sep = ""), append = FALSE, sep =
",", dec = ".", quote = FALSE, col.names = TRUE, row.names = FALSE)}

# Calculate SET contribution (i.e. what proportion of the required SET is
contributed by each record) and test for the occurrnce of extreme events capable
of causing mortality (i.e. exceeding ULT or LLT).
for (NumberLDT in 1 : length(LDT)) {
    for (i in 1 : length(DevTime.Temperature.Temporary)) {
        if ((DevTime.Temperature.Temporary[i] > LDT[NumberLDT]) &
(DevTime.Temperature.Temporary[i] < UDT[NumberLDT])) {
          SET.contrib[i, NumberLDT] = ((DevTime.Temperature.Temporary[i] -
LDT[NumberLDT]) * DevTimes.TTS$TimeChange[i]) / SET[NumberLDT]} else
{SET.contrib[i, NumberLDT] = 0}}
    Mortality.events[which((DevTime.Temperature.Temporary <= LLT[NumberLDT]) |
(DevTime.Temperature.Temporary >= ULT[NumberLDT])), NumberLDT] <- -1}

SiteRep <- rep(as.numeric(paste(Site[ColNumber], ".", SegNumber, sep = "")),
length(DevTimes.TTS[, 1]))
DevTimes.TTS <- cbind(SiteRep, DevTimes.TTS, DevelopmentTimes,
DevelopmentRecords, SET.contrib)


## Calculate mean development rate and period

# Option to alter how the start of the generation time period should match the
```

date-time values of the observed time series: 5 = match to closest day, 7 = match to closest 10th of day. This choice will make very little difference to results, except for species that develop over the course a few days (in which case use option "7" if temporal data were recorded at a suitably frequent interval).

```
time.match.accuracy = 5
match.end.accuracy = 2
```

# First create a vector containing code to identify all the different "seasons" (i.e. periods over which development should be assessed). In this set of analyses, it is assumed that parameters will be calculated over a year (although not necessarily starting on 1 Jan) - however, the option exists to specify a period shorter (or longer) than one year where the growing season / developmental period  does not span one year.

```
if (Do.calculate.generations.each.year == "y") {
  Seasons <- as.vector(rep(NA, length(DevTimes.TTS$DateTime)))
  Season.counter = 0
  gen.list <- vector("list", length(min(DevTimes.TTS$Year) :
max(DevTimes.TTS$Year)) * length(LDT))
  for (gen.year in min(DevTimes.TTS$Year) : max(DevTimes.TTS$Year)) {
		Generations.StartDate1 <- paste(Generations.StartDate, "/",
gen.year, " 00:00:00", sep = "")
		Generations.StartDate2 <- strptime(Generations.StartDate1,
format = "%d/%m/%Y %H:%M:%S", tz = "GMT")
		Generations.EndDate1 <- paste(Generations.EndDate, "/",
gen.year, " 23:59:59", sep = "")
		Generations.EndDate2 <- strptime(Generations.EndDate1, format ="
%d/%m/%Y", tz = "GMT")
		if (Generations.StartDate2 > Generations.EndDate2) {if
(gen.year%%4 == 0) {Generations.EndDate2 = Generations.EndDate2 + (60 * 60 * 24
* 365)} else {Generations.EndDate2 = Generations.EndDate2 + (60 * 60 * 24 *
366)}}
		if (as.numeric(min(julian(DevTimes.TTS$DateTime, origin =
as.POSIXct("1970-01-01 00:00:00", tz = "GMT")))) >
as.numeric(julian(Generations.StartDate2, origin = as.POSIXct("1970-01-01
00:00:00", tz = "GMT")))) {
	   Generations.StartDate.julian <-
match(as.numeric(strtrim(min(julian(DevTimes.TTS$DateTime[DevTimes.TTS$Year ==
gen.year], origin = as.POSIXct("1970-01-01 00:00:00", tz = "GMT"))),
time.match.accuracy)), as.numeric(strtrim(julian(DevTimes.TTS$DateTime, origin =
as.POSIXct("1970-01-01 00:00:00", tz = "GMT")), time.match.accuracy)))
	} else {
	   Generations.StartDate.julian <-
match(as.numeric(strtrim(julian(Generations.StartDate2, origin =
as.POSIXct("1970-01-01 00:00:00", tz = "GMT")), time.match.accuracy)),
as.numeric(strtrim(julian(DevTimes.TTS$DateTime, origin = as.POSIXct("1970-01-01
00:00:00", tz = "GMT")), time.match.accuracy)))}
	if (as.numeric(max(julian(DevTimes.TTS$DateTime, origin =
as.POSIXct("1970-01-01 00:00:00", tz = "GMT")))) <
as.numeric(julian(Generations.EndDate2, origin = as.POSIXct("1970-01-01
00:00:00", tz = "GMT")))) {
```

}



```
          Generations.EndDate.julian <- 
match(as.numeric(strtrim(max(julian(DevTimes.TTS$DateTime[DevTimes.TTS$Year == 
gen.year], origin = as.POSIXct("1970-01-01 00:00:00", tz = "GMT"))), 
time.match.accuracy + match.end.accuracy)), 
strtrim(as.numeric(julian(DevTimes.TTS$DateTime, origin = as.POSIXct("1970-01-01 
00:00:00", tz = "GMT"))), time.match.accuracy + match.end.accuracy))
      } else {
          Generations.EndDate.julian <- 
match(as.numeric(strtrim(julian(Generations.EndDate2, origin = 
as.POSIXct("1970-01-01 00:00:00", tz = "GMT")), time.match.accuracy + 
match.end.accuracy)), as.numeric(strtrim(julian(DevTimes.TTS$DateTime, origin = 
as.POSIXct("1970-01-01 00:00:00", tz = "GMT")), time.match.accuracy + 
match.end.accuracy)))}
                Season.counter = Season.counter + 1
                Seasons[Generations.StartDate.julian : 
Generations.EndDate.julian] <- Season.counter
  }
}

# Next create a vector containing code to identify just one period (if required)
if (Do.calculate.generations.one.year == "y") {
        OneSeason <- as.vector(rep(NA, length(DevTimes.TTS$DateTime)))
  Generations.StartDate5 <- strptime(Generations.StartDate4, format = "%d/%m/%Y 
%H:%M:%S", tz = "GMT")
        Generations.EndDate5 <- strptime(Generations.EndDate4, format = 
"%d/%m/%Y %H:%M:%S", tz = "GMT")
        if (as.numeric(min(julian(DevTimes.TTS$DateTime, origin = 
as.POSIXct("1970-01-01", tz = "GMT")))) > 
as.numeric(julian(Generations.StartDate5, origin = as.POSIXct("1970-01-01", tz = 
"GMT")))) {
      Generations.StartDate.julian <- 1
  } else {
        Generations.StartDate.julian <- 
match(as.numeric(strtrim(julian(Generations.StartDate5, origin = 
as.POSIXct("1970-01-01", tz = "GMT")), time.match.accuracy)), 
strtrim(as.numeric(julian(DevTimes.TTS$DateTime, origin = 
as.POSIXct("1970-01-01", tz = "GMT"))), time.match.accuracy))}
  if (max(julian(DevTimes.TTS$DateTime, origin = as.POSIXct("1970-01-01", tz = 
"GMT"))) < julian(Generations.EndDate5, origin = as.POSIXct("1970-01-01", tz = 
"GMT"))) {
      Generations.EndDate.julian <- length(DevTimes.TTS$DateTime)
  } else {
        Generations.EndDate.julian <- 
match(as.numeric(strtrim(julian(Generations.EndDate5, origin = 
as.POSIXct("1970-01-01", tz = "GMT")), time.match.accuracy + 
match.end.accuracy)), strtrim(as.numeric(julian(DevTimes.TTS$DateTime, origin = 
as.POSIXct("1970-01-01", tz = "GMT"))), time.match.accuracy + 
match.end.accuracy))}
        OneSeason[Generations.StartDate.julian : Generations.EndDate.julian] <- 
1
}
```

```
# Next, create matrices to hold results
Dev_colnames <- "n"
Dev_colnames2 <- c("MeanDevRate", "MeanDevPeriod", "MinDevPeriod", 
"Generations")
for (i in 1 : length(LDT)) {Dev_colnames <- c(Dev_colnames, paste(Dev_colnames2, 
DevTimes.ColNames[i], sep = "_"))}

DatasetDevelopmentRate <- matrix(NA, ncol = 2 + (4 * length(LDT)), nrow = 1)
YearlyDevelopmentRate <- matrix(NA, ncol = 3  + (4 * length(LDT)), nrow = 
length(unique(TTS$Year)))
colnames(DatasetDevelopmentRate) <- c("Site", Dev_colnames)
colnames(YearlyDevelopmentRate) <- c("Site", "Year", Dev_colnames)

if (Do.calculate.generations.each.year == "y") {
  SeasonalDevelopmentRate <- matrix(NA, ncol = 4  + (4 * length(LDT)), nrow = 
length(unique(Seasons[-c(which(is.na(Seasons)))])))
  colnames(SeasonalDevelopmentRate) <- c("Site", "StartDate", "EndDate", 
Dev_colnames)}
if (Do.calculate.generations.one.year == "y") {
  OneSeasonDevelopmentRate <- matrix(NA, ncol = 4  + (4 * length(LDT)), nrow = 
1)
  colnames(OneSeasonDevelopmentRate) <- c("Site", "StartDate", "EndDate", 
Dev_colnames)}

# If any temperatures exceed ULT or LLT, the create vector to record those 
events
Mortality.events.matrix <- matrix(NA, ncol = length(LDT), nrow = 
length(DevTimes.TTS$TimeChange))
for (i in 1 : length(LDT)) {
    Mortality.events.record[[i]] <- which(Mortality.events[, i] == -1)
    if(length(Mortality.events.record[[i]]) == 0) {
        Mortality.events.record[[i]] <- c(1, length(DevTimes.TTS$TimeChange))
    } else {
        if(Mortality.events.record[[i]][1] != 1) Mortality.events.record[[i]] <- 
c(1, Mortality.events.record[[i]])
        if(Mortality.events.record[[i]][length(Mortality.events.record[[i]])] != 
length(DevTimes.TTS$TimeChange))  Mortality.events.record[[i]] <- 
c(Mortality.events.record[[i]], length(DevTimes.TTS$TimeChange))
    }
    for (j in 1 : (length(Mortality.events.record[[i]]) - 1)) {
        if (j == 1) Mortality.events.matrix[j : (Mortality.events.record[[i]][2] 
- 1), i] <- j
        if (j == (length(Mortality.events.record[[i]]) - 1)) 
Mortality.events.matrix[(Mortality.events.record[[i]][j] + 1) : 
Mortality.events.record[[i]][j + 1], i] <- j
        if (j != 1 & j != (length(Mortality.events.record[[i]]) - 1)) 
Mortality.events.matrix[(Mortality.events.record[[i]][j] + 1) : 
(Mortality.events.record[[i]][j + 1] - 1), i] <- j
        Mortality.events.matrix[which(Mortality.events[, i] == -1), i] <- -1}}

# Calculate parameters for the whole dataset
DatasetDevelopmentRate[1, 1] <- as.numeric(paste(Site[ColNumber], ".", 
```

```
SegNumber, sep = ""))
DatasetDevelopmentRate[1, 2] <- length(DevTimes.TTS[, 1])
for (i in 1 : length(LDT)) {
  DatasetDevelopmentRate[1, 3 + (4 * (i - 1))] <- mean(24 * DevTimes.TTS[, 12 +
(2 * length(LDT)) + i] / DevTimes.TTS$TimeChange)
  DatasetDevelopmentRate[1, 4 + (4 * (i - 1))] <- 1 / DatasetDevelopmentRate[1,
3 + (4 * (i - 1))]
  DatasetDevelopmentRate[1, 5 + (4 * (i - 1))] <-
min(DevTimes.TTS[which(DevTimes.TTS[, 12 + i] > 0), 12 + i], na.rm = TRUE)
  if (any(Mortality.events.matrix[, i] == -1)) {
      summed.generations <- 0
      for (k in 1 : (max(Mortality.events.matrix[, i], na.rm = TRUE))) {
          if(any(Mortality.events.matrix[, i] == k)) {
              summed.generations <- summed.generations +
floor(sum(DevTimes.TTS[Mortality.events.matrix[, i] == k, 12 + (2 * length(LDT))
+ i]))}}
      DatasetDevelopmentRate[1, 6 + (4 * (i - 1))] <- summed.generations
  } else {
      DatasetDevelopmentRate[1, 6 + (4 * (i - 1))] <- floor(sum(DevTimes.TTS[,
12 + (2 * length(LDT)) + i]))}}


# Repeat calculations per year
YearlyDevelopmentRate[, 1] <- rep(as.numeric(paste(Site[ColNumber], ".",
SegNumber, sep = "")), length(unique(TTS$Year)))
for (j in 1 : length(unique(TTS$Year))) {
  for (i in 1 : length(LDT)) {
    YearlyDevelopmentRate[j, 2] <- unique(TTS$Year)[j]
    YearlyDevelopmentRate[j, 3] <- length(which(DevTimes.TTS$Year ==
unique(TTS$Year)[j]))
    YearlyDevelopmentRate[j, 4 + (4 * (i - 1))] <- mean(24 *
DevTimes.TTS[DevTimes.TTS$Year == unique(TTS$Year)[j], 12 + (2 * length(LDT)) +
i] / DevTimes.TTS$TimeChange[DevTimes.TTS$Year == unique(TTS$Year)[j]])
    YearlyDevelopmentRate[j, 5 + (4 * (i - 1))] <- 1 / YearlyDevelopmentRate[j,
4 + (4 * (i - 1))]
    YearlyDevelopmentRate[j, 6 + (4 * (i - 1))] <-
min(DevTimes.TTS[DevTimes.TTS[, 12 + i] > 0 & DevTimes.TTS$Year ==
unique(TTS$Year)[j], 12 + i], na.rm = TRUE)
    if (any(Mortality.events.matrix[DevTimes.TTS$Year == unique(TTS$Year)[j], i]
== -1)) {
        summed.generations <- 0
        for (k in 1 : (max(Mortality.events.matrix[, i], na.rm = TRUE))) {
            if(any(Mortality.events.matrix[DevTimes.TTS$Year ==
unique(TTS$Year)[j], i] == k)) {
                summed.generations <- summed.generations +
floor(sum(DevTimes.TTS[Mortality.events.matrix[, i] == k & DevTimes.TTS$Year ==
unique(TTS$Year)[j], 12 + (2 * length(LDT)) + i]))}}
        YearlyDevelopmentRate[j, 7 + (4 * (i - 1))] <- summed.generations
    } else {
        YearlyDevelopmentRate[j, 7 + (4 * (i - 1))] <-
floor(sum(DevTimes.TTS[DevTimes.TTS$Year == unique(TTS$Year)[j], 12 + (2 *
length(LDT)) + i]))}}}
```

```
# Repeat calculations per season
if (Do.calculate.generations.each.year == "y") {
  SeasonalDevelopmentRate[, 1] <- rep(as.numeric(paste(Site[ColNumber], ".",
SegNumber, sep = "")), length(unique(Seasons[-c(which(is.na(Seasons)))])))
  for (j in unique(Seasons[-c(which(is.na(Seasons)))])) {
    for (i in 1 : length(LDT)) {
      SeasonalDevelopmentRate[j, 2] <-
as.character(format(min(DevTimes.TTS$DateTime[which(Seasons == j)]), format =
c("%m/%d/%y")))
      SeasonalDevelopmentRate[j, 3] <-
as.character(format(max(DevTimes.TTS$DateTime[which(Seasons == j)]), format =
c("%m/%d/%y")))
      SeasonalDevelopmentRate[j, 4] <- length(which(Seasons == j))
      SeasonalDevelopmentRate[j, 5 + (4 * (i - 1))] <- mean(24 *
DevTimes.TTS[which(Seasons == j), 12 + (2 * length(LDT)) + i] /
DevTimes.TTS$TimeChange[which(Seasons == j)])
      SeasonalDevelopmentRate[j, 6 + (4 * (i - 1))] <- 1 /
as.numeric(SeasonalDevelopmentRate[j, 5 + (4 * (i - 1))])
      SeasonalDevelopmentRate[j, 7 + (4 * (i - 1))] <-
min(DevTimes.TTS[which((DevTimes.TTS[, 12 + i] > 0) & Seasons == j &
!is.na(Seasons)) , 12 + i])
      if (any(Mortality.events.matrix[Seasons == j] == -1, na.rm = TRUE)) {
          summed.generations <- 0
          for (k in 1 : (max(Mortality.events.matrix[, i]))) {
              if(any(Mortality.events.matrix[Seasons == j, i] == k, na.rm =
TRUE)) {
                  summed.generations <- summed.generations +
floor(sum(DevTimes.TTS[Mortality.events.matrix[, i] == k & Seasons == j &
!is.na(Seasons), 12 + (2 * length(LDT)) + i]))}}
          SeasonalDevelopmentRate[j, 8 + (4 * (i - 1))] <- summed.generations
      } else {
          SeasonalDevelopmentRate[j, 8 + (4 * (i - 1))] <-
floor(sum(DevTimes.TTS[which(Seasons == j), 12 + (2 * length(LDT)) + i]))
      }}}}

# Repeat calculations for one specific season
if (Do.calculate.generations.one.year == "y") {
  OneSeasonDevelopmentRate[1, 1] <- as.numeric(paste(Site[ColNumber], ".",
SegNumber, sep = ""))
  for (i in 1 : length(LDT)) {
    OneSeasonDevelopmentRate[1, 2] <-
as.character(format(min(DevTimes.TTS$DateTime[which(OneSeason == 1)]), format =
c("%m/%d/%y")))
    OneSeasonDevelopmentRate[1, 3] <-
as.character(format(max(DevTimes.TTS$DateTime[which(OneSeason == 1)]), format =
c("%m/%d/%y")))
    OneSeasonDevelopmentRate[1, 4] <- length(which(OneSeason == 1))
    OneSeasonDevelopmentRate[1, 5 + (4 * (i - 1))] <- mean(24 *
DevTimes.TTS[which(OneSeason == 1), 12 + (2 * length(LDT)) + i] /
DevTimes.TTS$TimeChange[which(OneSeason == 1)])
    OneSeasonDevelopmentRate[1, 6 + (4 * (i - 1))] <- 1 /
```

```
as.numeric(SeasonalDevelopmentRate[1, 5 + (4 * (i - 1))])
    OneSeasonDevelopmentRate[1, 7 + (4 * (i - 1))] <-
min(DevTimes.TTS[which((DevTimes.TTS[, 12 + i] > 0) & OneSeason == 1 &
!is.na(OneSeason)) , 12 + i])
    if (any(Mortality.events.matrix[OneSeason == 1, i] == -1, na.rm = TRUE)) {
        summed.generations <- 0
        for (k in 1 : (max(Mortality.events.matrix[, i]))) {
            if(any(Mortality.events.matrix[OneSeason == j, i] == k, na.rm =
TRUE)) {
                summed.generations <- summed.generations +
floor(sum(DevTimes.TTS[Mortality.events.matrix[, i] == k & OneSeason == j &
!is.na(OneSeason), 12 + (2 * length(LDT)) + i]))}}
        OneSeasonDevelopmentRate[1, 8 + (4 * (i - 1))] <- summed.generations
    } else {
        OneSeasonDevelopmentRate[1, 8 + (4 * (i - 1))] <-
floor(sum(DevTimes.TTS[which(OneSeason == 1), 12 + (2 * length(LDT)) + i]))}}}

# Creat output files containing development rates and times summaries
if (file.exists(paste(OutputFileName, "_DatasetDevelopment.csv", sep = ""))) {
    write.table(DatasetDevelopmentRate, file = paste(OutputFileName,
"_DatasetDevelopment.csv", sep = ""), append = TRUE, sep = ",", dec = ".", quote
= FALSE, col.names = FALSE, row.names = FALSE)
} else {
    write.table(DatasetDevelopmentRate, file = paste(OutputFileName,
"_DatasetDevelopment.csv", sep = ""), append = FALSE, sep = ",", dec = ".",
quote = FALSE, col.names = TRUE, row.names = FALSE)}

if (file.exists(paste(OutputFileName, "_YearlyDevelopment.csv", sep = ""))) {
    write.table(YearlyDevelopmentRate, file = paste(OutputFileName,
"_YearlyDevelopment.csv", sep = ""), append = TRUE, sep = ",", dec = ".", quote
= FALSE, col.names = FALSE, row.names = FALSE)
} else {
    write.table(YearlyDevelopmentRate, file = paste(OutputFileName,
"_YearlyDevelopment.csv", sep = ""), append = FALSE, sep = ",", dec = ".", quote
= FALSE, col.names = TRUE, row.names = FALSE)}

if (Do.calculate.generations.each.year == "y") {
    if (file.exists(paste(OutputFileName, "_SeasonalDevelopment.csv", sep =
""))) {
        write.table(SeasonalDevelopmentRate, file = paste(OutputFileName,
"_SeasonalDevelopment.csv", sep = ""), append = TRUE, sep = ",", dec = ".",
quote = FALSE, col.names = FALSE, row.names = FALSE)
    } else {
        write.table(SeasonalDevelopmentRate, file = paste(OutputFileName,
"_SeasonalDevelopment.csv", sep = ""), append = FALSE, sep = ",", dec = ".",
quote = FALSE, col.names = TRUE, row.names = FALSE)}}

if (Do.calculate.generations.one.year == "y") {
    if (file.exists(paste(OutputFileName, "_OneSeasonDevelopment.csv", sep =
""))) {
        write.table(OneSeasonDevelopmentRate, file = paste(OutputFileName,
"_OneSeasonDevelopment.csv", sep = ""), append = TRUE, sep = ",", dec = ".",
```

```
quote = FALSE, col.names = FALSE, row.names = FALSE)
    } else {
        write.table(OneSeasonDevelopmentRate, file = paste(OutputFileName,
"_OneSeasonDevelopment.csv", sep = ""), append = FALSE, sep = ",", dec = ".",
quote = FALSE, col.names = TRUE, row.names = FALSE)}}

} # closes loop to run (overall) development times module
} # close loop through segments
} # close loop through time series


##################
# CODE ENDS HERE #
##################
```
